# Single-cell analysis of gastric pre-cancerous and cancer lesions reveals cell lineage diversity and intratumoral heterogeneity

**DOI:** 10.1038/s41698-022-00251-1

**Published:** 2022-01-27

**Authors:** Jihyun Kim, Charny Park, Kwang H. Kim, Eun Hye Kim, Hyunki Kim, Jong Kyu Woo, Je Kyung Seong, Ki Taek Nam, Yong Chan Lee, Soo Young Cho

**Affiliations:** 1 National Cancer Center, 323 Ilsan-ro, Goyang-si, Gyeonggi-do 10408 Republic of Korea; 2 Severance Biomedical Science Institute, Brain Korea 21 PLUS Project for Medical Science, Yonsei University College of Medicine, Seoul, 03722 Republic of Korea; 3 Institute of Gastroenterology, Yonsei University College of Medicine, Seoul, Republic of Korea; 4 Department of Internal Medicine, Severance Hospital, Seoul, Republic of Korea; 5 Department of Pathology, Yonsei University College of Medicine, Seoul, Republic of Korea; 6 Korea Mouse Phenotyping Center (KMPC), Seoul National University, Seoul, Republic of Korea; 7 Laboratory of Developmental Biology and Genomics, Research Institute of Veterinary Science, BK21 Program Plus for Advanced Veterinary Science, College of Veterinary Medicine, Seoul National University, Seoul, Republic of Korea; 8 Interdisciplinary Program for Bioinformatics, Program for Cancer Biology and BIO-MAX institute, Seoul National University, Seoul, 08826 Republic of Korea; 9 Department of Molecular and Life Science, Hanyang University, Ansan, 15588 Republic of Korea

**Keywords:** Cancer genomics, Gastric cancer

## Abstract

Single-cell transcriptomic profiles analysis has proposed new insights for understanding the behavior of human gastric cancer (GC). GC offers a unique model of intratumoral heterogeneity. However, the specific classes of cells involved in carcinogenetic passage, and the tumor microenvironment of stromal cells was poorly understood. We characterized the heterogeneous cell population of precancerous lesions and gastric cancer at the single-cell resolution by RNA sequencing. We identified 10 gastric cell subtypes and showed the intestinal and diffuse-type cancer were characterized by different cell population. We found that the intestinal and diffuse-type cancer cells have the differential metaplastic cell lineages: intestinal-type cancer cells differentiated along the intestinal metaplasia lineage while diffuse-type cancer cells resemble de novo pathway. We observed an enriched *CCND1* mutation in premalignant disease state and discovered cancer-associated fibroblast cells harboring pro-stemness properties. In particular, tumor cells could be categorized into previously proposed molecular subtypes and harbored specific subtype of malignant cell with high expression level of epithelial-myofibroblast transition which was correlated with poor clinical prognosis. In addition to intratumoral heterogeneity, the analysis revealed different cellular lineages were responsible for potential carcinogenetic pathways. Single-cell transcriptomes analysis of gastric pre-cancerous lesions and cancer may provide insights for understanding GC cell behavior, suggesting potential targets for the diagnosis and treatment of GC.

## Introduction

Gastric cancer (GC) is the fourth most commonly occurring human cancer and the second most common cause of cancer-related deaths worldwide, despite the global decrease in its incidence^1^. In general, GC incidence rapidly increases in the elderly, with a divergent peak in the young ages^2,3^. According to the Lauren classification, GC is divided into the intestinal and diffuse types (IGC and DGC, respectively), and mixed-type GC is very rare^1^. A carcinogenetic pathway for IGC driven by multiple events has been proposed (Correa’s hypothesis) with chronic superficial gastritis progressing into chronic atrophic gastritis (CAG), intestinal metaplasia (IM), dysplasia, and ultimately carcinoma^1^. However, atrophic changes have not been closely correlated with DGC^4^, and the mechanism underlying DGC development is poorly understood.

Recent studies incorporated in The Cancer Genome Atlas (TCGA) and Asian Cancer Research Group (ACRG) revealed the usefulness of next-generation sequencing for the molecular classification of GC, and identified its prognostic significance in independent cohorts^5,6^. Epigenomic and genomic studies on the premalignant state of GC revealed distinctive patterns of gene expression and DNA methylation, which can be used to investigate carcinogenesis risk and progression markers for patients with IM^7–9^. Systematic analysis of the tumor microenvironment (TME) infiltrate patterns in 1524 patients with GC revealed that TME phenotypes are correlated with genomic characteristics^10^. Single-cell RNA sequencing (scRNA-seq) has been used to characterize the heterogeneity of the GC cell population and TME^11,12^. A inter- and intra-heterogeneity for TME provide the transcriptional diversity with macrophages and cytotoxic T cells^13^. Also, the intra-genomic heterogeneity in GC provides the diversity of GC cell lineages in GC patients and 12-gene signature appears to be fundamental to GC carcinogenesis as it is not only highly prognostic in GC cohort but performed just as robustly in several large scale localized GC cohorts^14^. However, all of these high-throughput methods used to analyze tumor plasticity have the limitations reproducing histopathology stages for the Correa’s hypothesis, extending prior to GC molecular subtypes in single cell resolution, and investigating inter-/intra-heterogeneity for IGC and DGC.

**Fig. 1:**
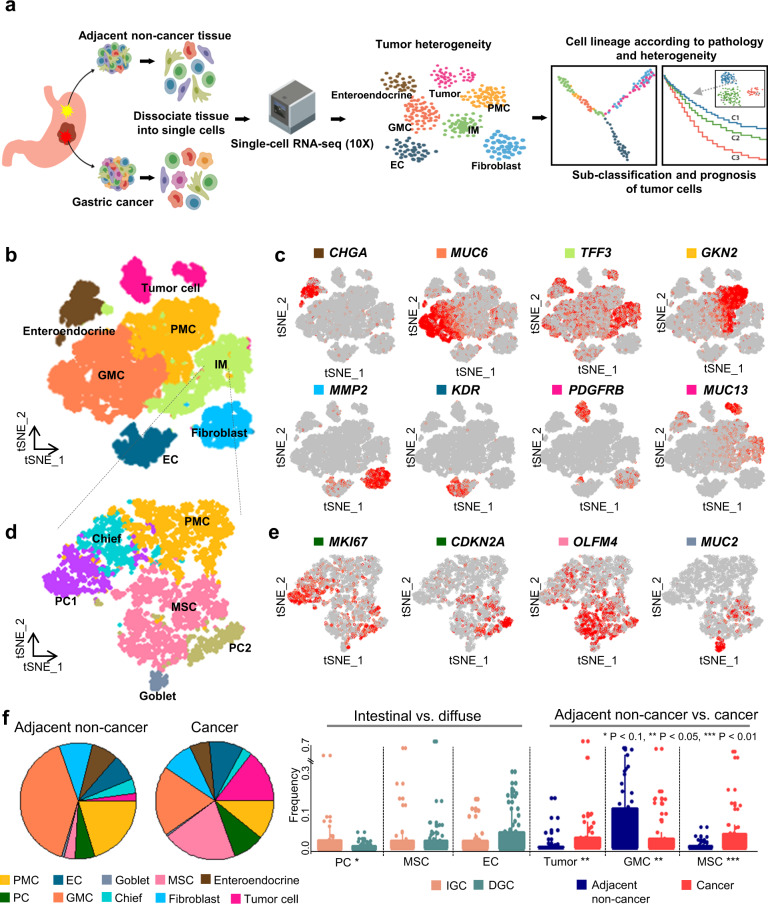
Single-cell profiling of adjacent normal and gastric cancer tissues. **a** Overview of single-cell RNA-seq analysis of the adjacent non-cancer (*n* = 24) and gastric cancer (GC) tissue (*n* = 24) from 24 patients. **b** t-Stochastic neighbor embedding (t-SNE) map of filtered 13,022 single cells in the adjacent non-cancer and cancer tissues. Colors represent cell types based on the expression of known marker genes: endothelial cell (EC), enteroendocrine, fibroblast, gland mucous cell (GMC), intestinal metaplasia (IM), tumor cell, pit mucous cell (PMC). **c** t-SNE plot showing the expression of marker genes for seven cell types. **d** t-SNE plot showing sub-clustering of IM cells: chief cells, goblet cells, metaplastic stem-like cells (MSCs), PMC, proliferative cell 1 (PC1), and proliferative cell 2 (PC2). **e** t-SNE plot showing the expression of marker genes for three cell types. *PGC* was highly expressed in chief cell (Supplementary Fig. 5b). **f** Pie charts represent the distribution of ten cell types in the adjacent normal tissue and cancer lesion (left). Bar plots show the frequency of specific cell types in different tissue comparisons (right). Points on the bar plots represent individual samples; *P* values were calculated by the *t* test.

Accordingly, the present study aimed to characterize the cellular heterogeneity in GC by single cell transcriptome analysis. To this end, we profiled the cancer cell landscape for adjacent precancerous and GC lesions. We also evaluated the tumor plasticity by analyzing cell populations of lineages categorized according to the Lauren classification. Finally, we classified the masked tumor cell signatures by single cell RNA sequencing (scRNA-seq) and bulk sequencing to identify the molecular markers of cell transition from the premalignant to malignant states in gastric carcinogenesis (Fig. 1a).

## Results

### Tumor plasticity of GC cell populations

From 24 gastric cancer (GC) patients, paired non-cancerous and cancer tissues were obtained (Methods). Among the 24 adjacent non-cancerous sites, 16 were classified as a premalignant lesion (CAG or IM) and eight showed benign superficial gastritis (Supplementary Table 1). GC patients showed signs of Epstein-Barr virus (EBV, 4.2%) or *Helicobacter pylori* infection (79.2%), but microsatellite instability (MSI) did not observed.

After quality control and filtering, we identified 30,888 cells from 24 GC patients. As previous studies already proposed the immunology driven GC prognosis with scRNA-seq^11–14^. A high proportion of immune cells was observed in patients with DGC (Supplementary Fig. 1a). We identified 7831 immune cells (46%) from DGC patients and 1296 immune cells (25.4%) from IGC patients. Diffuse-type samples had displayed high immune scores in previous bulk RNA-seq studies^5,6,10^; similarly, DGC samples from TCGA-STAD and ACRG datasets used in this study were shown to display high immune scores (Supplementary Fig. 1b). We identified six immune cell types, including B cells, dendritic cell (DC), monocytes, macrophages, NK cells, and T cells (Supplementary Fig. 2). The proportions of DCs, monocytes, and T cells increased in cancer tissues, and the amount B cells increased in non-cancerous tissues. We performed sub-clustering analysis to determine the type of T cells present and found that the population of CD8 + T central memory (Tcm) cells decreased and that of CD4 + Tcm cells increased in tumor tissue. It has been reported that CD8 + Tcm to confer superior antitumor immunity compared with effector memory T cells^15^, and that the proportion of CD4 + Tcm increases in the blood of GC patients^16^.

We excluded immune cells (CD45-positive) for our analysis and focused more on tumor and non-immune stromal cells (Methods). Further clustering of tumor cells and stromal fibroblasts disclosed unique subclasses that might be relevant to GC’s malignant features. To elucidate the cellular heterogeneity of GC cells, we select 13,022 cells annotated stromal (fibroblast, endothelial and endocrine cells), epithelial and tumor cells excluding immune cells from distinct lesions (Methods). Of these, 7095 cells (54.5%) originated from matched adjacent non-cancerous lesions and 5927 cells (45.5%) originated from GC lesions. Clustering analysis identified 10 distinct sub-clusters. The cell types were characterized as endothelial cell (EC; expressing *PLVAP*, *KDR*, *PTPRB*), enteroendocrine cell (expressing *CHGA*, *GAST, PROX1*), fibroblast (expressing *MMP2*, *PDGFRA*, *MYL9*, *FN1*, *CAV1*), gland mucous cell (GMC; expressing *MUC6* and *TFF2*), IM (intestinal metaplasia; expressing *TFF3*, *CDX1*, *CDX2*) [chief (expressing *PGC*), goblet (expressing *MUC2*, *ITLN1*, *HES6*), metaplastic-stem cell (MSC; expressing *OLFM4*, *REG1A*, *CLDN3*), and proliferative cell (PC; expressing *CDKN2A*, *MKI67*, *RBP4*)], pit mucous cell (PMC; expressing *GKN1*, *GKN2*, *MUC5AC*), and tumor cell (expressing *EPCAM*, *CDH17*, *COL3A1*, *PDGFRB*), based on the DEG from each cluster to known marker genes of various cell types (Fig. 1b–e, Methods). Cells from each cluster were unbiased distributed among different patients (Supplementary Fig. 3). We performed proportion tests for the number of patients in each cluster and found that the sample frequency for each cell cluster was not significantly high or low. The CNVs were estimated to distinguish tumor cell cluster (Supplementary Methods), and we found high CNV signals in tumor cell cluster compare to the other cell types (Supplementary Fig. 4a). We confirmed common CNV signals from TCGA STAD dataset. A total of 12 genes were identified as common CNVs from the GISTIC2 results for the TCGA STAD data set^5^. We found that *CD44, CDK6, GATA4, GATA6, KLF5*, and *KRAS*, which were amplified in TCGA STAD cohort, were amplified in cancer cell clusters. *ARID1A* and *SMAD4*, which annotated deletions in the TCGA STAD cohort, were inferred to be deleted in the cancer cell cluster (Supplementary Fig. 4b). We then, defined the IM cluster (Fig. 1b, c and Supplementary Fig. 5a) based on *CDX1* and *CDX2* expression, and then further clustered them into sub-IM cell types. Among 4115 cells, six clusters were identified and classified into five cell types (Fig. 1d, e) namely chief, goblet, MSC, PMC, and PC. The major cell types of the IM cluster were MSC (34.4%), PMC (30.4%) and PC (21.6%, PC1: 13.5% and PC2:8.1%).

We next investigated the cell type distribution according to lesion type (adjacent non-cancer vs. GC) and the Lauren classification (IGC vs. DGC) (Fig. 1f, Supplementary Fig. 1c, and Supplementary Table S9). The PC cluster in IGC was bigger than that in DGC (*P* = 0.06) and the EC cluster in IGC was smaller than that in DGC (*P* = 0.12). However, the frequencies and correlation coefficients of other cell types except for PC, tumor cells, GMC, and MSC did not show any significant differences by the lesion type or Lauren classification (Supplementary Fig. 5d, e). In adjacent non-cancer lesions, GMCs represented the largest proportion of cells, which was significantly reduced in GC lesions (*P* = 0.04, *t* Test; Fig. 1e); PMCs showed a similar pattern (*P* = 0.08). The proportions of MSCs and tumor cells significantly increased in GC lesions compared to adjacent non-cancer lesions (*P* = 0.003 and 0.02, respectively). Furthermore, IM cells formed two distinct clusters representing cells from the adjacent non-cancer and GC lesions. MSCs were enriched in GC lesions (Supplementary Fig. 5c). Notably, 2.1% of cells from the adjacent non-cancer lesions were tumor cells. We found that tumor cells in the adjacent non-cancer lesions were detected in five patients with IGC (56%) and in 11 patients with DGC (73%). Some non-malignant cells (GMC, PMC, chief, and PC cells) were consistently observed in both adjacent non-cancer and GC lesions. The presence of tumor cells in adjacent non-cancer lesions was strongly correlated with MSCs and goblet cells, but these relationships were not as pronounced in GC lesions (Supplementary Fig. 5d). PC was divided into two types: PC1 and PC2. The PC1 cluster showed high expression in *TFF1* and *TFF2*, where most cells were obtained from non-cancerous tissues. The PC2 cluster showed high expression in *TFF3* and *REG4*, where cells were obtained from the cancer tissue of intestinal patients. Zhang et al. identified two PC clusters: gastric proliferative cells and intestinal proliferative cell^11^. *TFF1* and *TFF2* were highly expressed in gastric proliferative cells, and *TFF3* and *REG4* were highly expressed in the intestinal proliferative cell cluster. These observations may suggest that the premalignant state of the adjacent non-cancerous mucosa comprises of IM and rare tumor cells, and our data have high reproducibility and provide reliable results. It could be suggested that GC represent a cellular population harboring spectrum of cell lines representing non-malignant, premalignant, and malignant states.

### Tracing GC cell lineages associated with the Lauren classification

**Fig. 2:**
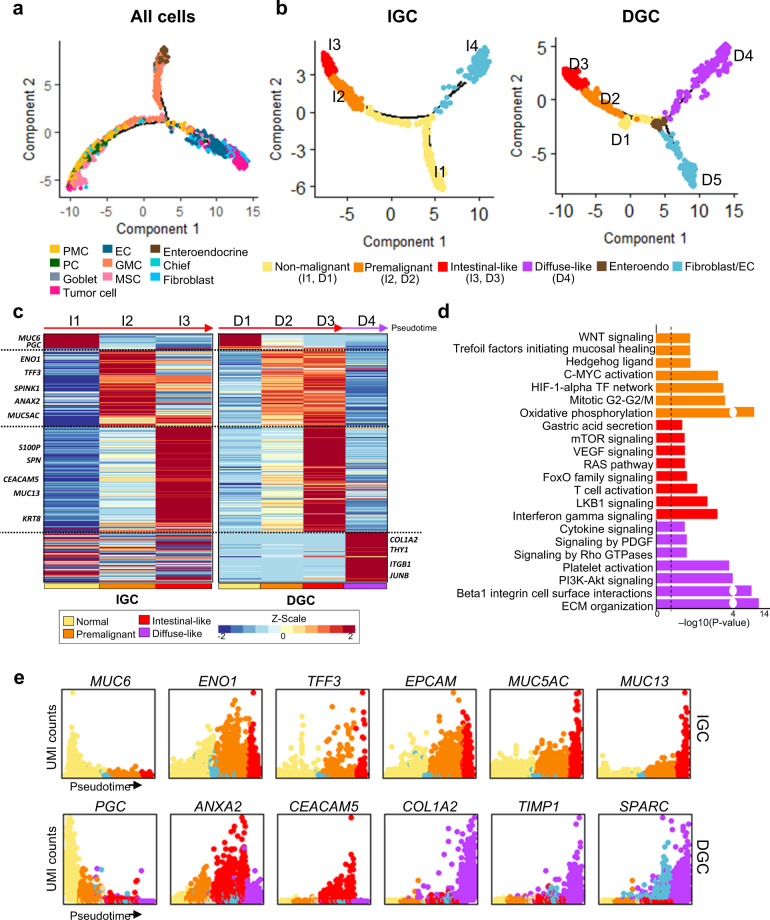
Tumor cell types determined by pathological classification and functional features. **a** Trajectory plot of a total of 13,022 cells. In the trajectory trees, colors represent ten cell types. **b** Trajectory plots for intestinal gastric cancer (IGC) and diffuse gastric cancer (DGC). Colors represent the malignant process (yellow: non-malignant, orange: premalignant, red: intestinal-like, and purple: diffuse-like) and other cell types (e.g., fibroblasts/ECs and enteroendocrine). The pseudotime increased from the non-malignant (I1 and D1) to the malignant state (I3, D3, and D4), except for fibroblasts and ECs. **c** Heatmap of differentially expressed genes (DEGs) derived from the malignant process-related states in IGC (I1–I3) and DGC (D1–D3 or D1– D4). Rows of the heatmap show dynamic expression changes of DEGs along the pseudotime, and known marker genes represent both sides in the heatmap. **d** Bar plots of significant pathways with DEGs enriched in specific cell states (threshold *P* < 0.05, black dotted line). *P* values were calculated by a gene enrichment test. Colors represent the malignant process (orange: premalignant, red: intestinal-like, and purple: diffuse-like). **e** Expression patterns of known marker genes related to the malignant process. The colors correspond to the malignant process states (orange: premalignant, red: intestinal-like, and purple: diffuse-like). The pseudotime is plotted along the *x* axis, and UMI gene counts are shown on the *Y* axis.

We next established a pseudotemporal trajectory to trace cell lineages (Supplementary Methods). As above description, GC and adjacent non-cancer lesions were collected from IGC and DGC patients. To investigate inter-heterogeneity of two Lauren types in GC, we pooled all selected cells (non-immune cell) from 24 samples and also analyzed lineage/state compositions for each IGC and DGC. The GC cell trajectory was reconstructed from a dataset of 13,022 using Monocle^17^ (Fig. 2a and Supplementary Fig. 6). The pseudotime increased from benign to malignant cell type. Non-malignant cells, such as enteroendocrine cells and GMCs, were showed earlier pseudotime in the cell lineage, and tumor cells were showed later pseudotime of the trajectory (Fig. 2a and Supplementary Fig. 6a). The expression levels of the known IGC markers *MUC13* and *CDH17*^18^ and the known DGC markers *COL1A2* and *SPARC*^19^ increased from GMCs to tumor cells in the tree (Supplementary Fig. 6b).

To reconstruct the IGC specific pseudotemporal trajectory, we selected 3686 cells from IGC patients (1340 cells from GC lesions and 2346 cells from adjacent non-cancer lesion; Fig. 2b). To identify the underlying characteristics of cancer progression according to cell lineage, the cells were divided into three progression states (I1, I2, and I3) based on the pseudotime, excluding fibroblasts and ECs. The non-malignant cells were located at the earlier pseudotime (I1) of the trajectory (Supplementary Fig. 7a). The expression levels of *ENO1* and *TFF3*, known IM markers^20^, were high in the I2 state and the expression levels of *CEACAM* and *MUC13*, known IGC markers^18^, were high in the I3 state (Fig. 2c, e). Fibroblasts, ECs, and some tumor cells were located at I4 (Supplementary Fig. 7a). We traced the neoplastic progression of cell lineage changes from IM (MSC and goblet cell) to the intestinal tumor cell (Fig. 2b). The expression levels of many IGC marker genes continually increased from the I1 to I3 state. Visualization of IGC cells in a trajectory tree revealed that the intermediate time-point IM cells were closer to tumor cells at the later time points than to GMC. Based on these observations, we propose that the IGC cell lineage shows a neoplastic progression pattern from IM to tumor, and that IM might be a precursor state in accordance with Correa’s hypothesis.

Next, we established the DGC trajectory using 8946 cells from DGC patients (4336 from GC lesions and 4610 from adjacent non-cancer lesion). Cells were arranged along four branches (Fig. 2b; right panel) and were divided into four progression states (D1, D2, D3, and D4) based on the pseudotime. The GMCs showed earlier pseudotime (D1) (Supplementary Fig. 7b), and fibroblasts and ECs were located at D5. The tumor cells were showed later pseudotime at trajectory tree (D3 and D4). DGC markers, such as *COL1A2*, were expressed in D4 (Fig. 2c, e)^19^, and the extracellular matrix–related pathway genes were significantly enriched (*P* = 1.11e–16). IGC marker genes, including *CEACAM5*, *KRT8*, and *MUC13*^18^, were highly expressed in D3 (Fig. 2c). Several IM-related pathways, including WNT signaling, Trefoil factors initiating mucosal healing, and HEDGEHOG ligand^21^, were enriched in the premalignant state I2 and D2 (*P* < 9.03e-03; Fig. 2d); mTOR signaling, RAS pathway, and VEGF signaling, functioning in IGC, were highly enriched in the I3 and D3 states (*P* < 0.01; Fig. 2d)^22^. To determine if the D1–D3 cells followed the intestinal cell lineage, we identified DEGs within each cascade. Genes involved in cancer progression overlapped irrespective of the pathological classification, excluding D4 state (Fig. 2c). Further, 84 DEGs (84.0%) in D3 cells overlapped with DEGs of the I3 cells (Supplementary Table 2); 72.6% of DEGs in D2 grouped in I2 and 99.6% of DEGs in D3 also grouped in I3. We calculated diffuse tumor dispersion and tumor cellularity for nine diffuse samples with IHC, and observed that average tumor cellularity was 58% (max: 100% and min: 30%) and average diffuse tumor dispersion was 81% (max: 100% and min: 70%) in DGC patients. These observations suggest that some of DGC tumors in this study are of mixed-type, though dispersion of intestinal tumor cell in DGC is small. We found that the cell lineage compositions of IGC are following the Correa’s hypothesis but DGC would have different carcinogenetic mechanism.

### Tumor cell subpopulations and epithelial–myofibroblast transition in DGC

**Fig. 3:**
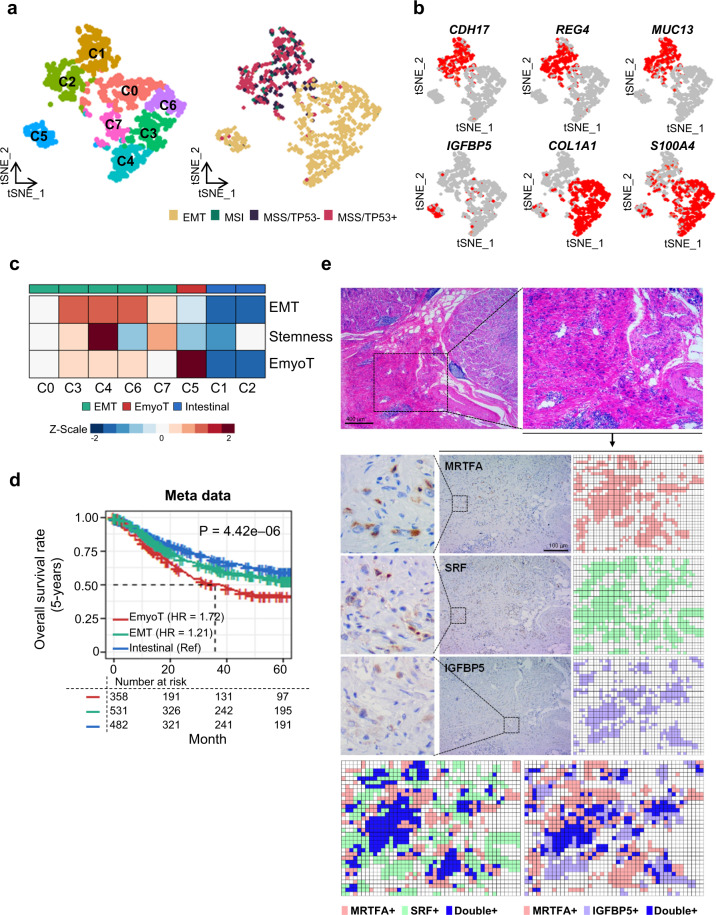
Tumor cell subgroups have distinct clinical outcomes. **a** t-Stochastic Neighbor Embedding (t-SNE) plot for the 1003 tumor cells. The left panel is the t-SNE plot for eight sub-clusters of tumor cells. On the right, all tumor cells were classified into four GC subtypes in ACRG. **b** t-SNE plot showing expression patterns of marker genes. **c** Functional characterization for eight sub-clusters. Average expression of cancer-associated signatures, including EMT, EmyoT and stemness within eight sub-clusters; the signature pattern shows three molecular subtypes (intestinal: blue, EMT: green, and EmyoT: red). **d** Five-year survival rates of three molecular subtypes based on bulk RNA metadata (*n* = 1378). Hazard ratios and *p* values were calculated by Cox regression; the intestinal group was used as the reference group. **e** Hematoxylin and eosin (H&E) and immunohistochemistry staining for SRF, IGFBP5, and MRTFA. Digital images constructed of staining results of each marker; overlapped location in digital images are represented at the bottom.

To understand the characteristics for the tumor cells, and extending prior GC molecular subtyping, we performed re-clustering of all defined tumor cells (Supplementary Fig. 8a), demonstrating eight sub-cell population (Fig. 3a). IGC markers (*CDH17*, *REG4*, and *MUC13*) were highly expressed in the C1 and C2, and DGC markers (*COL1A1* and S100A4) were highly expressed the C0 and C3–C7, respectively (Fig. 3b and Supplementary Table 3)^18,19^. Nearly all tumor cells were categorized into the prior GC molecular subtypes, called ACRG subtypes using ACRG signatures^6^ (MSS/TP53^+^, MSS/TP53^–^, MSI, and EMT; Supplementary Methods) from deconvolution method, as anticipated (Fig. 3a, right panel and Supplementary Fig. 8b). Cells in C1 and C2, which showed high expression of IGC marker genes, had a high signature score for MSS/TP53^+^ and MSS/TP53^–^, the major molecular phenotypes of IGC^6^ (Supplementary Fig. 8b). Cells in C0, C3, C4, C6, and C7, which showed high expression of DGC marker genes, had a pronounced EMT signature, the major molecular phenotype of DGC^23^. Furthermore, 30 MSI cells were identified, which were scattered and enriched in C1 and C2 (*P* = 4.18e–09; Fisher’s exact test). We inferred that the MSI cell type observed low frequency in our samples, in accordance with the pathological test. These results suggest that four major molecular subtypes can be reproducible at single cell level.

Molecular characterization was identified stem cell (SC)-related signatures in the tumor cells population. We analyzed SCs-related signatures in tumor cells, including target gene expression of the embryonic stem (ES) cell-like transcriptional factors involving NANOG homeobox, octamer-binding transcription factor 4 (OCT4), SRY-related HMG-box2 (SOX2), and MYC, to obtain genetic evidence of the presence of SCs population. Those genes sets targeted by four ES cell regulators were activated both in human ES cells and tumors^24^. The target genes of NANOG, OCT4, SOX2, and NOS were mainly expressed in C3, C4, and C7 (Fig. 3c and Supplementary Fig. 8c). The SCs-related and EMT signatures were up-regulated in C3, C4, and C7 (Fig. 3c). Recent reports indicate that the emergence of cancer SCs occurs in part as a result of EMT, and that gastric EMT activation could endow gastric epithelial cells with a cancer cell stemness property^25,26^. Therefore, SCs-related signature may represent in GC, which may increase the heterogeneity of GC and support the existence of SC signature at single cell resolution.

A group of cells (C5) that did not express EMT signatures (Fig. 3c and Supplementary Fig. 8d), but highly expressed myofibroblast-associated genes such as *TAGLN* and *EGR1* (Supplementary Fig. 8e) was identified in the tumor cell clusters analysis^27–29^. These C5 cells were enriched in markers of the epithelial–myofibroblast transition (EmyoT), in which normal epithelial cells transdifferentiate into myofibroblastic cells in cancerous transformation (Supplementary Table 4 and Fig. 3c)^29^.

Analysis of combinded GC cohort data indicated that the EmyoT (C5) type was significantly correlated with poor overall survival (Supplementary Methods; Fig. 3d). Survival analysis revealed that the EmyoT type was associated with the worst prognosis (*P* = 4.42e–06; HR = 1.72 and 95% CI 1.39–2.12, compared to intestinal type), followed by the EMT (HR = 1.21 and 95% CI 0.99–1.49 by log rank test), and intestinal type of GC in the combined GC cohort data. The statistical analysis of the independent extensive GC cohort meta-analysis data also confirmed our findings that EmyoT-type GC is associated with poor clinical outcome (Supplementary Fig. 9). The EMT type was previously reported to be correlated with poor prognosis^30^, which was inconsistent with the current data. This disagreement is likely due to the difficulty of defining EmyoT type cells in bulk diffuse-type tumor samples. This demonstrates that deconvolution analysis of the identified gene sets is strongly predictive of GC prognosis. The EmyoT subpopulation was also identified in samples from GC patients by immunohistochemistry (IHC; Methods). Nine of ten EmyoT type from diffuse-type tumors harbored MRTFA^+^ cells, an EmyoT marker^29^ (Supplementary Table 5). We then used IHC to analyze proteins encoded by differentially expressed genes in C5, including SRF and IGFBP5. Morphologically, SRF and IGFBP5 were present in the diffuse-type tumor cells, with some overlap with MRTFA^+^ cells (Fig. 3e). These observations indicated that EmyoT cells might represent one of the transdifferentiation processes in DGC.

### *CCND1* mutation profile in the IGC cell lineage

**Fig. 4:**
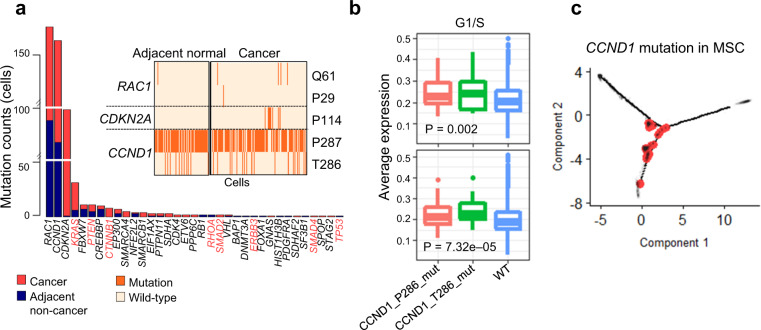
Association between *CCND1* mutation and the intestinal tumor cell lineage. **a** Mutation counts for 35 pan-cancer hotspot genes. Genes denoted in red along the *x* axis harbor mutations according to a TCGA gastric cancer study^5^. Inset plot represents the cells with mutations in *RAC1*, *CDKN2A*, and *CCND1* in the adjacent non-cancer tissue and cancer samples. **b** G1/S gene scores for cells with *CCND1* hotspot mutations in premalignant cells (upper) and tumor cells (bottom). *P* values were calculated by ANOVA. **c** Cells with mutations in *CCND1*, especially MSCs, mapped on a trajectory tree of IM and tumor cells. Red dots indicate mutated cells.

We identified 574 variants of 35 oncogenes as hotspot mutations in GC cells (Methods; Fig. 4a)^31^, which were enriched in the cell cycle, MAPK signaling, and other signaling pathways (Supplementary Tables 6 and 7). Cell cycle-related genes, including *CCND1*, *CDK4*, *CDKN2A*, and *RB1*, were frequently mutated in MSCs, PCs, and tumor cells (Supplementary Fig. 10a). *CCND1* was frequently mutated at two gain-of-functional sites (T286 and P287; Fig. 4a). These mutations drive cellular transformation, nuclear export, and proteasome-mediated degradation^32–34^. Further, *CCND1*_*mut*_ activates the G1/S pathway and regulation of G1/S pathway genes in GC carcinogenesis^35–37^, and the expression levels of these genes were increased in *CCND1*_*mut*_ cells (Fig. 4b; Supplementary Fig. 10b). The trajectory of premalignant and malignant cells reconstructed from 5048 cells, including IM cells, revealed three branches (Supplementary Fig. 10c). The malignant cells divided between two branches, and premalignant cells (MSCs and PCs) were arranged at the trajectory center. The *CCND1*_*mut*_ cells were traced from MSCs to malignant cells (Fig. 4c). The CNV signal for *CCND1* was not identified across cell types (Supplementary Fig. 4b). These were suggested that *CCND1*_*mut*_ could be potentially a risk factor of IGC and might play as an oncogenic driver in MSCs.

To validate the role of *CCND1*_*mut*_ in the premalignant state, we performed scRNA-seq to analyze samples from 5 control individuals who were diagnosed as having IM, but no evidence of GC development within 5 years of clinical follow-up (Supplementary Table 1). We identified eight cell clusters including immune cells such as B and T cell clusters in this group, with the IM cluster further divided into goblet, chief, and MSC clusters (Supplementary Fig. 10d). Interestingly, *CCND1*_*mut*_ cells (T286 and P287) were not identified among MSCs in this control group (Supplementary Table 6). Collectively, we propose that the cells in the control group is close to the cancer-free state and *CCND1*_*mut*_ in MSCs could be a molecular signature of premalignant state transition in IGC.

### Cancer-associated fibroblast subtypes and their roles in GC

**Fig. 5:**
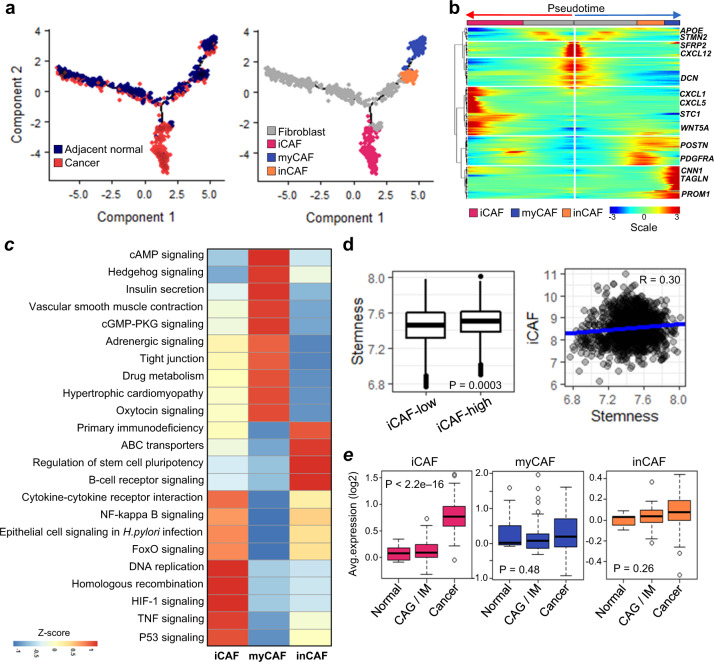
Cancer-associated fibroblast (CAF) heterogeneity and pro-stemness in gastric cancer. **a** Trajectory trees of CAF reconstructed using annotated fibroblasts and endothelial cells. Colors correspond to the malignancy (left) and CAF types (right). **b** Heatmap plot showing expression of significantly variable genes (*P* < 1.0e–05; likelihood ratio test) and known CAF markers. Collected cells (columns) are sorted by pseudotime, and the genes (rows) are clustered by hierarchical clustering. **c** Hallmark pathways of the CAF subtypes determined by enrichment analysis. Significance was determined by the R package limma (adjusted *P* < 0.05). **d** Box plot of the stemness score in bulk RNA metadata (left), and correlation between iCAFs and the stemness scores (right). **e** Boxplots of CAF marker gene expression patterns plotted using bulk RNA data (GSE2669), according to the gastric diseases progression cascade (i.e., normal, premalignant, Intestinal Metaplasia (IM) or chronic atrophic gastritis (CAG), and cancer). *P* values were calculated by ANOVA.

In GC, cancer-associated fibroblasts (CAFs) are one of the critical components of the TME that promote or impede tumorigenesis^38^. To better understand their roles in GC, we selected annotated fibroblasts from IGC and DGC clusters (Supplementary Fig. 5a) and reconstructed a pseudotemporal trajectory to trace CAF differentiation. The CAF trajectory was split into three branches, representing four states (Fig. 5a). Fibroblast cell marker genes such as *SFRP2* and *CXCL12* were expressed in the gray region of the trajectory (Fig. 5a; right panel), and CAF markers were specifically expressed at the two branch ends (Fig. 5b). We identified three CAF types based on the states of gene expression profiles: inflammatory (iCAF), myofibroblastic (myCAF), and intermediate CAFs (inCAF) (Fig. 5a). Genes encoding cytokines (*IL6*, *IL11*, and *IL24*), chemokines (*CXCL1*, *CXCL2*, *CXCL5*, and *CXCL6*)^39^, and matrix metalloproteinases (*MMP1*, *MMP3*, and *MMP10*)^40^ were uniquely upregulated in iCAFs (Fig. 5b; Supplementary Fig. 11a). Furthermore, myCAFs were identified by known marker genes such as *TPM1*, *TPM2*, *MYL9*, *TAGLN*, and *POSTN*^29^ (Fig. 5b and Supplementary Fig. 11a). Finally, inCAFs were distinctly divided between iCAFs and myCAFs, with high expression of *PDGFRA*, *POSTN*, *ID1*, and *ID3* (Fig. 5b and Supplementary Fig. 11a); *PDGFRA* is a commonly used iCAF marker in different cancer types^41^ and *ID1* is activated in myCAF^42^.

iCAFs were more prevalent in DGC than in IGC (43.1% vs. 20.3%, *P* = 0.0001; Fisher’s exact test), while the reverse was observed for myCAFs (36.5% vs. 24.1%, *P* = 0.02; Fisher’s exact test). However, the difference of the inCAF prevalence between IGC and DGC was not significant. In addition, iCAFs mainly comprised cells from GC lesions, but distribution of other cells with myCAFs and inCAFs did not differ in GC or adjacent non-cancer lesions. iCAFs are reported to be closely associated with GC invasion and promoting stemness of tumor cells^29,43^. *IL6*, *CXCL1*, and *CXCL2* are biomarkers of pro-stemness CAFs^43^, and these markers were highly expressed in iCAFs (Supplementary Fig. 11a). Pro-stemness-associated pathways, including NF-kappa B signaling, TNF signaling, and cytokine–cytokine receptor interaction pathways, were enriched in iCAFs^44^ (Fig. 5c). Next, we observed an association between iCAFs and stemness in the GC cohort data based on DEGs with CAF subtypes (Supplementary Table 8), and stemness scores in the iCAF-high group were higher than those in the iCAF-low group (Fig. 5d and Supplementary Fig. 11b). Furthermore, iCAF scores were positively correlated with the stemness scores (R = 0.3; Fig. 5d), whereas myCAF and inCAF were negatively correlated with stemness (Supplementary Fig. 11d). These observations indicate that the stemness in GC could be promoted by an increasing iCAF signature, and especially, the iCAF expression is strongly associated with stemness in DGC (Supplementary Fig. 11c). The intratumoral heterogeneity of CAFs may provide evidences that CAFs are involved in complex tumor structures and paracrine interactions in the TME.

Finally, independent microarray data for 124 normal, IM, CAG, and GC samples was analyzed (GSE2669) to verify the existence of three different CAF subtypes in GC. We were able to identify three iCAF, myCAF, and inCAF signatures in these samples, as well (Supplementary Table 8). In 57 GC patients, all CAF signatures were elevated in GC lesions. Specifically, the iCAF signal was pronounced in GC (Fig. 5e; *P* < 2.2e–16) during cancer progression (normal, CAG, IM, and GC). The myCAF and inCAF signals were not significantly different (Fig. 5e) in cancer but the inCAF signal increased with tumor progression from the premalignant state onward.

## Discussion

Overall, the present study confirms several important observations from previous in vivo, in vitro, and large-cohort genomics studies, offering a comprehensive catalog of gastric cells in the adjacent non-cancer and GC lesions at single-cell resolution to describe GC cellular heterogeneity and carcinogenetic pathways.

Identification of the cell subtypes and reconstruction of Correa’s hypothetical pathways are yielded several findings. First, GC cells are more complex and heterogeneous than previously reported. GC presents ten cell types comprising several subpopulations with highly homogenous non-malignant cells. The adjacent non-cancerous site also contains a small number of tumor cells, which was consistent with previous study showing that patients diagnosed with IM harbor some tumor cells at the single-cell level^11^. Although the Lauren classification is reproducible, at single-cell resolution, DGC harbors an appreciable proportion of intestinal-type tumor cells. These findings might be explained the lack of noticeable variation between IGC and DGC in bulk genomics studies previously reported.

IGC and DGC are exhibited by different cell lineage composition. Tumor cells are characterized by a variety of neoplastic progression pathway and de novo carcinogenetic pathway, as demonstrated by the trajectory and sub-clustering analysis based on gene expression profiles. Each cell lineage had a differential gene expression profile, phenotype, and functional characteristics. In the intestinal-type cell lineage, tumor cells progress from IM, with a gradual increase in cellular heterogeneity in the malignant stage. In the diffuse-type cell lineage, SCs-related signatures interact with intratumoral CAFs and can evolve into different cell populations to survive. DGC heterogeneity could be induced by SCs-related signature and results in progression to GC. We propose that GC heterogeneity may be associated with these independent cellular lineage characteristics, which can inform lineage-specific tailored therapy for GC patients.

Most of the tumor cells from our GC samples could be categorized into previously proposed GC molecular subtypes with expected clinicopathological behavior. In addition, we identified a unique population of cancer cells, the EmyoT type, characterized by a distinct gene expression profile with significant clinical implications. The EmyoT tumor cell type was associated with diffuse marker gene expression, a pronounced EmyoT signature, and weak EMT signature, and correlated with a poor clinical prognosis. Furthermore, tumor cell signatures robustly correlated with survival in various cohorts, suggesting the clinical adoption of these subtype-specific genes as biomarkers for treatment modality and prognosis prediction.

Moreover, our results suggest that *CCND1*_*mut*_ and iCAFs may play a role in neoplastic lineage changes in GC. In the intestinal cell lineage, *CCND1*_*mut*_ in MSCs may have an oncogenic role from the premalignant state onward, associated with increased cell cycle damage. In both IGC and DGC, CAFs emerge in the premalignant state, and their numbers increase in the malignant state. In addition, iCAFs exhibit pro-stemness properties in DGC and might participate in de novo carcinogenesis in DGC. These findings could be used to identify new markers to identify high-risk groups and to monitor GC progression from the premalignant state.

However, we have limitation for biological validation, including cell biological assays and in vivo analysis in knockout mouse model. This is another significant project for a new investigation of gastric carcinogenesis and an understanding of human GC carcinogenesis. We believe that our results will be provide new insight to cancer biologist and provide inspiration for GC carcinogenesis. The GC cell atlas generated in this study provides a new reference point for the investigation of gastric carcinogenesis, and will contribute to the understanding of human GC carcinogenesis, including organoids and humanized GC mouse models.

## Methods

### Samples and sample pre-processing for single-cell RNA extraction

This study was approved by the Institutional Review Board of Severance Hospital (IRB 4-2017-1131) and written informed consent was obtained from each participant. Freshly biopsied gastric mucosal specimens were obtained from two sites (cancer site and non-cancerous adjacent site) by conventional upper gastrointestinal endoscopy using SwingJaw biopsy forceps (Olympus Medical System, Tokyo, Japan, 2.45 mm). Human stomach tissues were cut into approximately 1-mm^3^ pieces, suspended in 5 ml of dispase (5 U/ml) in Hanks’ balanced salt solution (STEMCELL, cat# 07913) with 5 mg collagenase type IV (Sigma, cat# C5138), and incubated at 37 °C for 2 h with gentle shaking. The samples were then passed through a 100-μm cell strainer (Falcon), the cells were re-suspended in phosphate-buffered saline (PBS) containing 1 mM ethylenediaminetetraacetic acid (EDTA), and centrifuged at 720 × *g* for 5 min. To remove red blood cells, cell pellets were re-suspended in PBS with 1 mM EDTA, and centrifuged through a discontinuous 50% Percoll (2.5 ml Percoll mixed with 2.5 ml PBS; Sigma, cat# P4937) gradient at 470 × *g* for 20 min, with minimum acceleration and no deceleration. The material between the PBS and 50% Percoll layers was collected and re-suspended in PBS for scRNA-seq. After cell isolation, all of the protocols such as cell barcoding and library construction were performed according to the manufacturer’s instructions for the 10× chromium single cell 3′ v2 kit and sequenced with the Illumina HiSeq 2500 platform.

### Single-cell quality control and data pre-processing

ScRNA-seq data was analyzed using the 10× Genomics software package Cell Ranger version 2.1.1. The data were mapped to the hg19 reference genome (v1.2.0) supplied by 10× Genomics. A gene count matrix was generated from unique molecular identifiers (UMIs) by Cell Ranger. The following thresholds were used to identify low-quality cells: (1) standard deviation of all genes per cell lower than 1; (2) zero UMI count for 90% of all genes; (3) 10% or more of the expression originates from the mitochondrial or hemoglobin genes; or (4) UMI values lower than 100 or larger than 20,000. The data matrix was normalized for sequencing depth by dividing by the total number of UMIs for each cell and then transformed to a log scale using the R package Seurat^45^. After normalization, the cells were split into immune and non-immune cells based on the average *PTPRC* (CD45) expression level. Principal components analysis was performed to select variable genes to reduce dimension complexity. Highly variable genes were identified using the FindVariableGenes function with parameters for x.low.cutoff = 0.0125, x. high.cutoff = 6, and y.cutoff = 0.5. These variable genes were used as inputs for PCA using the RunPCA function with parameters for seed.use = 12345 and pcs.compute = 30. The first 20 principal components and a resolution of 0.8 were used for clustering using FindClusters. The principal components were then used to generate a two-dimensional representation using t-distributed stochastic neighbor embedding (tSNE). Cluster analyses were performed using the RunTSNE function in the Seurat package with parameter dims.use = 1:5 and seed.use = 12345. To find differentially expressed genes (DEGs) for each cluster, the likelihood-ratio test for cell clusters was implemented using the FindAllMarkers function (parameters: genes detected in at least 25% cells, and differential expression threshold of 0.25 log fold change using Wilcoxon rank sum test with *P* < 0.05 following Bonferroni correction), with selected genes showing at least 2-fold up-regulation and false discovery rate (FDR) < 0.01 compared to those in the remaining clusters (Supplementary Tables 2 and 3). We compared the marker genes for each cluster to literature-based markers of cell lineages to assign a cell lineage per cluster^6,11^. The tumor cell was identified marker gene expression and CNV signal (Supplementary Methods).

Trajectory analysis was performed to track the cell transition status (Supplementary Methods). Cell data was reprocessed to remove low-UMI count genes or low-quality cells and re-normalized for library size using the R package Monocle^17^. After quality control, dimensionality reduction and trajectory construction were then performed. Cells were placed onto a pseudotime trajectory using the orderCells function. A secondary cluster analysis of selected cell population were repeated same process (detection of variable genes, scaling with UMI regression, PCA, clustering, and tSNE).

### CG meta-cohort construction for validation

The GC meta-cohort was constructed by 1378 bulk-seq dataset from GEO (GSE13861, GSE66229, GSE26899, GSE26901, GSE28541, GSE29272, and GSE84437) and TCGA STAD dataset. The GC meta-cohort was normalized and eliminated batch effect (Supplementary Methods). To determine the cell type proportions in bulk gene expression profiles, the MuSic deconvolution method was used^46^ with the tumor subtype-specific gene signatures (Supplementary Table S3).

### Visualization of EmyoT in situ

To determine the multi-protein–positive zone in diffuse-type tumor lesions, IHC staining from consecutive sections were performed. Images (10×) of each EmyoT marker (SRF, IGFBP5, MRTFA) from the same tumor area in representative tumors were further analyzed. Images were aligned and divide into 1530 tiles (45 × 34) to mimic the approximate size of a tumor cell and each tile was annotated in each color to the stained position in the section. The images of the EmyoT markers were overlapped, and double-positive tiles (MRTFA + IGFBP + or MRTFA + SRF + ) were annotated in blue.

### Single-cell mutation calling and calculation of mutation allele frequency

To detect single cell variants, we used the VarTrix tool (https://github.com/10xgenomics/vartrix). Variant allele frequencies (VAF) in known somatic hotspots of oncogenes were calculated using the following equation: VAF = mutation allele read count/(mutation allele read count + reference allele read count). After calling the mutations in samples, mutations with zero read counts compared with the reference was filtered out, including immune cells. To extract molecular cancer drivers, 4463 somatic hotspots in 394 oncogenic driver genes and pathways identified previously in a pan-cancer study^31^ were considered. To evaluate the expression of genes involved in the cell cycle, such as the G1/S phase pathway genes, the appropriate gene lists were obtained from the MsigDB database^47^.

### Reporting summary

Further information on research design is available in the Nature Research Reporting Summary linked to this article.

## Supplementary information

supplemental material

REPORTING SUMMARY

## Data Availability

All single-cell transcriptome data sets in this study have been deposited in the Gene expression Omnibus (GEO) database (GSE150290).

## Code availability

The code that supports the findings of this study are available from the corresponding authors on reasonable request.
