# Supplementary material for: Single-cell analysis of gastric pre-cancerous and cancer lesions reveals cell lineage diversity and intratumoral heterogeneity
Source: NPJ Precis Oncol. 2022 Jan 27;6:9. doi: 10.1038/s41698-022-00251-1 (PMC8795238; doi:10.1038/s41698-022-00251-1)
Supplement: Supplementary file 1 — supplemental material [file 41698_2022_251_MOESM1_ESM.pdf]

Supplemental Material

1. **Supplementary Methods**
2. **Supplementary Figures**
3. **Supplementary Tables**

1. Supplementary Methods

Hematoxylin-eosin and immunohistochemical (IHC) staining.

The human stomach tissue samples were fixed with 10% buffered paraformaldehyde and then embedded in paraffin. The tissues were sectioned at a thickness of 5 μm, deparaffinized with xylene three times for 20 min each, 100% EtOH three times for 10 min each, 90% EtOH twice for 10 min each, and 75% EtOH for 10 min, and stained with hematoxylin and eosin. Stained tissue slides were dehydrated and mounted with Shandon Synthetic Mount (Thermo, Cat# 6769007). For immunohistochemistry, paraffin-embedded samples were sectioned at 3 μm, deparaffinized, and rehydrated in PBS. Antigens were then retrieved for 15 min at high pressure in Target Retrieval Solution (Dako, Cat# S1699). Subsequently, the specimens were chilled on ice for 1 h, washed with PBS three times for 5 min each, and blocked with 3% H_2O_2 in PBS for 30 min to quench the endogenous peroxidase. The slides were washed again with PBS, blocked for 2 h at room temperature with Serum-Free Protein Block (Dako, Cat# X0909), probed at 4°C overnight with the primary antibodies at 1/1000 dilution (anti-SRF, Cell signaling Tech, Cat# D71A9; anti-IGFBP5, R&D systems, Cat# AF875; anti-MRTFA, Abcam, Cat# ab49311), stained for 30 min with horseradish peroxidase (HRP)-conjugated anti-rabbit IgG (Dako, Cat# K4003) or Goat-IgG VECTASTAIN ABC-HRP Kit (Vector Cat# PK-4005), and developed with Liquid DAB+ Substrate Chromogen System (Dako, Cat# K3468). Finally, the specimens were counterstained with Mayer's hematoxylin (Dako, Cat# S3309) and mounted with Shandon Synthetic Mount (Thermo Cat# 6769007).

Microsatellite instability in gastric cancer tissues was assessed immunohistochemically [1]. IHC was performed using a Ventana XT automated stainer (Ventana Corporation) with antibodies to MLH1

(ready to use, clone M1, Roche, Indianapolis, IN, USA), MSH2 (ready to use, clone G219-1129, Roche), MSH6 (1:100, clone 44, Cell Marque, Rocklin, CA, USA), and PMS2 (1:40, clone MRQ28, Cell Marque). Sections were deparaffinized using EZ Prep solution (Ventana Corporation). CC1 standard (pH 8.4 buffer containing Tris/borate/EDTA) was used for antigen retrieval and blocked with inhibitor D (3% H2O2) for 4 min at 37°C. Slides were incubated with primary antibody for 40 min at 37°C followed by a universal secondary antibody for 20 min at 37°C. Slides were incubated in streptavidin-horseradish peroxidase (SA-HRP) D for 16 min at 37°C and then the substrate, 3,3'-diaminobenzidine tetrahydrochloride (DAB) H2O2, was added for 8 min followed by hematoxylin and bluing reagent counterstaining at 37°C. A loss of MMR protein expression (MMR-deficiency) was designated when none of the neoplastic epithelial cells showed nuclear staining, whereas normal expression was defined as the presence of nuclear staining of tumor cells, irrespective of the proportion or intensity. Infiltrating lymphocytes, stromal cells and adjacent non-neoplastic epithelium served as internal positive controls.

EBV-encoded RNA in situ hybridization (EBER ISH) was performed with a Ventana BenchMark in situ hybridization system (ISH iView kit, Ventana, Tucson, AZ, USA)[1]. Paraffin-embedded tissue sections were deparaffinzed with EZ Prep buffer (Ventana), and then digested with protease I for 4 min. Probes were applied and then denaturation was performed at 85 °C (10 min), followed by hybridization at 37 °C (1 h). The probes labeled with fluorescein contained a cocktail of oligonucleotides dissolved in a formamide-based diluent. After hybridization, tissues were washed 3 times with 2× saline sodium citrate buffer at 57 °C. Incubation with antifluorescein monoclonal antibody was performed for 20 min and then an Alkaline Blue detection kit (Ventana) was used according to the manufacturer's protocol. The slides were counterstained with Nuclear Fast Red for 10 min.[2]

Estimation of copy number variations

To infer copy number variation (CNV) with each cell using single-cell RNA sequencing data, we applied the R package inferCNV [3]. The monocyte cells from immune cell type were used as reference cells. CNV scores were re-standardized and performed min-max normalization ranged as -1 to 1. To estimate CNV signals with both amplifications and deletions, the scores of each cell was calculated as

quadratic sum [4].

Trajectory analysis and cell classification

Trajectory analysis was performed to track the cell transition status. Cell data were reprocessed to remove low-UMI count genes or low-quality cells and re-normalized for library size using the R package Monocle [5]. After quality control, dimensionality reduction and trajectory construction were then performed. Cells were placed onto a pseudotime trajectory using the orderCells function. The pseudotime trajectory was inferred from the root cells comprising the annotated non-malignant cells (e.g., PMC and GMC). A secondary cluster analysis of selected cell population were repeated same process (detection of variable genes, scaling with UMI regression, PCA, clustering, and tSNE).

To analyze cells ordered along the DGC trajectory, DGC cells were classified using IGC DEGs to verify whether the biological function of DGC cells is the same as that of IGC cells. The data were trained on state-annotated IGC cells (I1–I3) based on the DEGs of the intestinal cell lineage and DGC data were tested using the RandomForest algorithm. DGC cells at the specific states were then predicted. Similar analyses were applied to tumor cell classification. For tumor cell analysis, tumor cells were classified into ACRG subtypes and known marker genes were used to predict tumor cell subtypes. All classification analyses were performed using the R package e1071.

DEG and pathway analysis

To identify DEGs in the intestinal cell lineage, linear and non-linear regression with second- and third-degree polynomial model analysis was performed for the non-malignant to tumor cells, based on the trajectory states. All regression models were fitted using the R function lm. The applied FDR correction was calculated using the R function p.adjust. In addition, the *t*-test was used to analyze differences between the states, excluding annotated cell states from trajectory analysis, such as fibroblasts, ECs, and enteroendocrine cells. Finally, DEG lists identified by the two statistical methods were merged. Pathway analysis of DEGs of each state (non-malignant, premalignant, and malignant) was performed using the Cytoscape plug-in ReactomeFI [6]. Pre-defined gene expression signatures related to cancer

biology, such as EMT, MSI, cytokine, TP53 [7], and EmyoT signatures, were obtained from the literature [8]. The gene signature lists are provided in Supplementary Table 4.

Deconvolution of bulk gene expression data

After identifying markers related to the different subtypes of tumor cells, such as EMT, EmyoT, and intestinal cells, their validity was checked by analyzing independent bulk-seq datasets. For the analysis, 1,378 bulk-seq datasets were obtained from the publicly available GEO database (GSE13861, GSE66229, GSE26899, GSE26901, GSE28541, GSE29272, and GSE84437; https://www.ncbi.nlm.nih.gov/geo/) and The Cancer Genome Atlas (TCGA) STAD dataset. Quantile normalization was first applied to each dataset, and voom transformation was used for RNA-seq data, such as that from TCGA [9]. To eliminate the dataset batch effect, combined gene expression profiling was performed using ComBat [10]. To determine the cell type proportions in bulk gene expression profiles, the MuSic deconvolution method was used [11] with the tumor subtype-specific gene signatures (Supplementary Table 3). The tumor subtypes were then assigned by hierarchical clustering using the subtype probabilities calculated from the bulk dataset. Survival analysis of the assigned cancer subtypes was performed by Cox regression using the R package survival.

Statistical analysis. All statistical analyses in this study were performed through R version 3.4.1 and inferCNV was conducted in R version 3.6.1; gene expression deconvolution analysis was conducted in R version 3.5.1.

References

1. Park, J.H., *et al.* Epstein-Barr virus positivity, not mismatch repair-deficiency, is a favorable risk factor for lymph node metastasis in submucosa-invasive early gastric cancer. *Gastric Cancer* **19**, 1041-1051 (2016).
2. Bailey, M.H., *et al.* Comprehensive Characterization of Cancer Driver Genes and Mutations. *Cell* **174**, 1034-1035 (2018).
3. Patel, A.P., *et al.* Single-cell RNA-seq highlights intratumoral heterogeneity in primary glioblastoma. *Science* **344**, 1396-1401 (2014).
4. Peng, J., *et al.* Author Correction: Single-cell RNA-seq highlights intra-tumoral heterogeneity and malignant progression in pancreatic ductal adenocarcinoma. *Cell Res* **29**, 777 (2019).
5. Qiu, X., *et al.* Single-cell mRNA quantification and differential analysis with Census. *Nat Methods* **14**, 309-315 (2017).
6. Wu, G., Dawson, E., Duong, A., Haw, R. & Stein, L. ReactomeFIViz: a Cytoscape app for pathway and network-based data analysis. *F1000Res* **3**, 146 (2014).
7. Cristescu, R., *et al.* Molecular analysis of gastric cancer identifies subtypes associated with distinct clinical outcomes. *Nat Med* **21**, 449-456 (2015).
8. Lamouille, S., Xu, J. & Derynck, R. Molecular mechanisms of epithelial-mesenchymal transition. *Nat Rev Mol Cell Biol* **15**, 178-196 (2014).
9. Law, C.W., Chen, Y., Shi, W. & Smyth, G.K. voom: Precision weights unlock linear model analysis tools for RNA-seq read counts. *Genome Biol* **15**, R29 (2014).
10. Chen, C., *et al.* Removing batch effects in analysis of expression microarray data: an evaluation of six batch adjustment methods. *PLoS One* **6**, e17238 (2011).
11. Wang, X., Park, J., Susztak, K., Zhang, N.R. & Li, M. Bulk tissue cell type deconvolution with multi-subject single-cell expression reference. *Nat Commun* **10**, 380 (2019).

Supplementary Figures

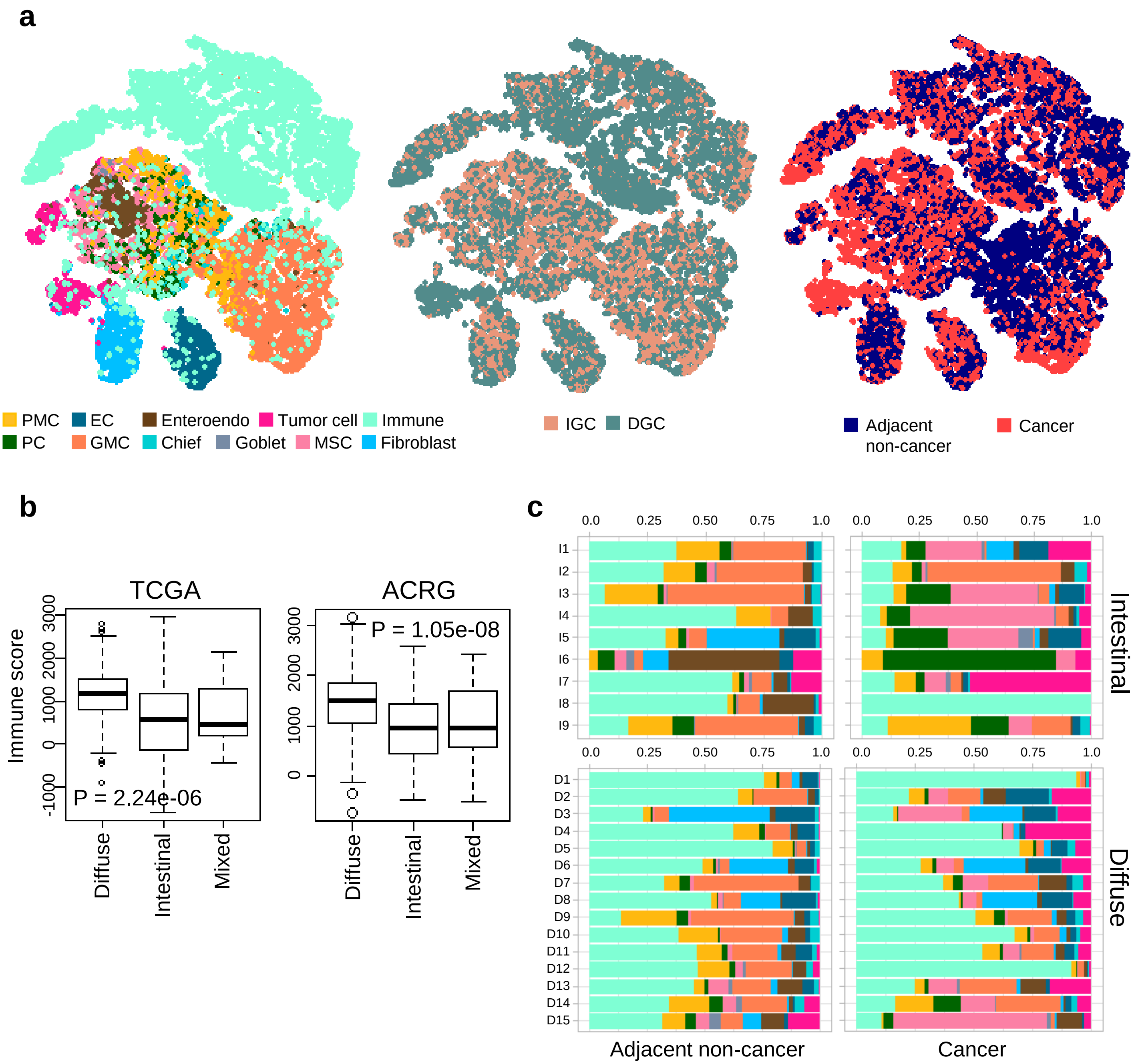

Supplementary Fig. 1. Heterogeneity of gastric cells from adjacent non-cancer and gastric cancer lesions. (GC) tissues. **a** Distribution of all cell clusters with cell types. Each color in the t-stochastic neighbor embedding (t-SNE) plot represents cell types (left), Lauren types (middle) and distinct lesions (right). **b** Immune scores with Lauren types from independent bulk RNA-sequencing using ESTIMATE. P-values were calculated by anova test. **c** Fractions of 11 cell types with malignancy and Lauren classification. Bar plots represent each patient with Lauren classification and malignancy separately.

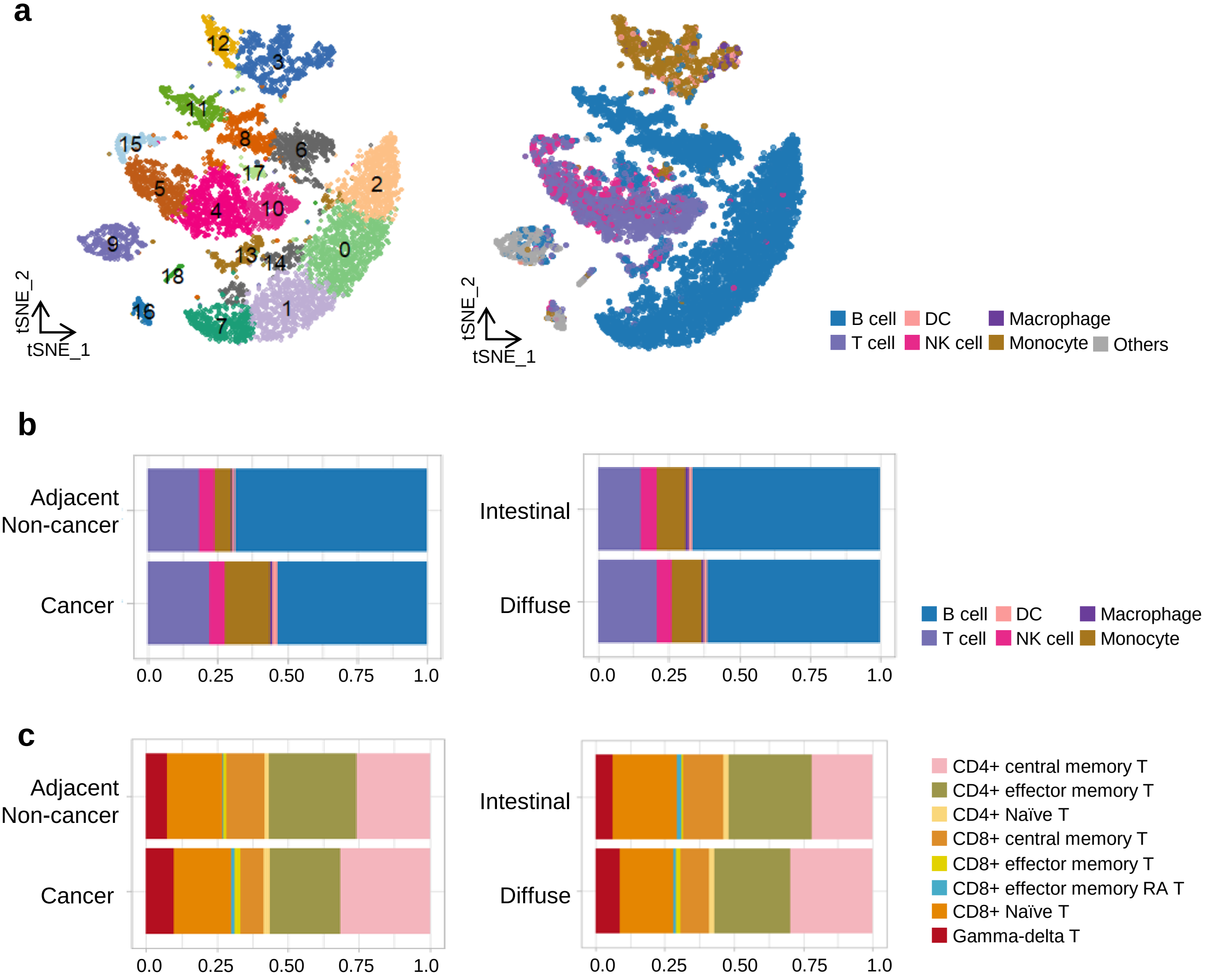

Supplementary Fig. 2. Immune cell sub-clustering and distribution. a Distribution of immune clusters. Each color in the t-stochastic neighbor embedding (t-SNE) plot represents a each cluster (left) and immune cell types (right). **b** Fractions of main 6 immune cell types with malignancy and Lauren classification. **c** Fractions of T immune sub-cell types with malignancy and Lauren classification.

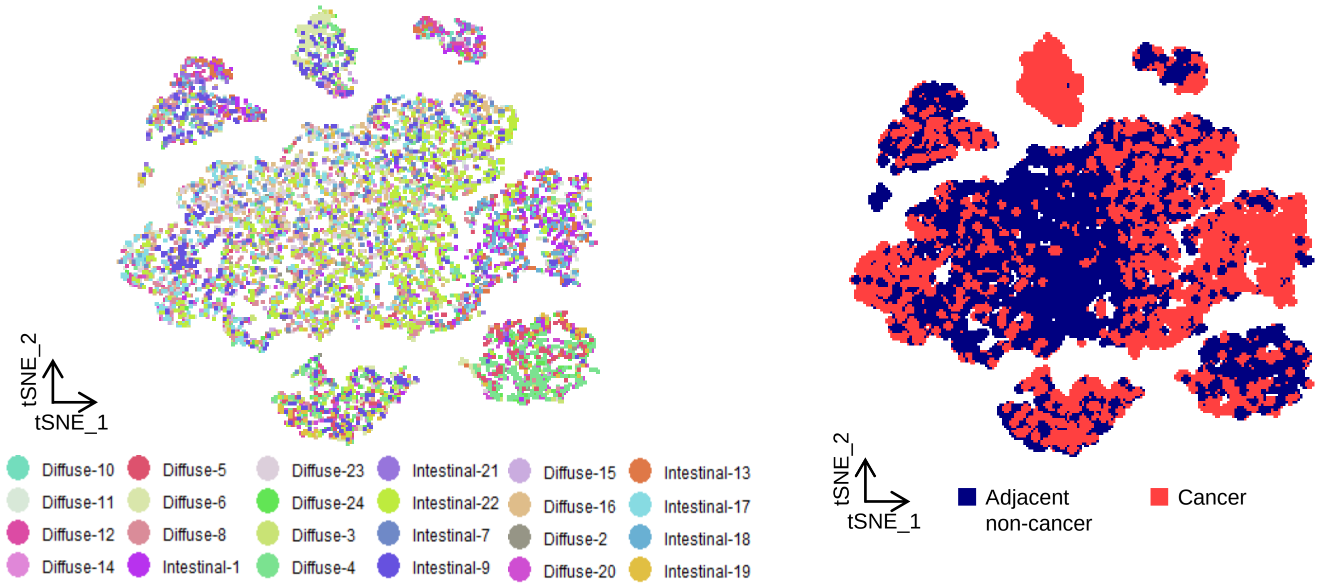

Supplementary Fig. 3. Heterogeneity of non-immune gastric cells from adjacent non-cancer and gastric cancer lesions. Distribution of cells for each patient in all clusters. Each color in the t-stochastic neighbor embedding (t-SNE) plot represents a patient (left) and distinct lesions (right).

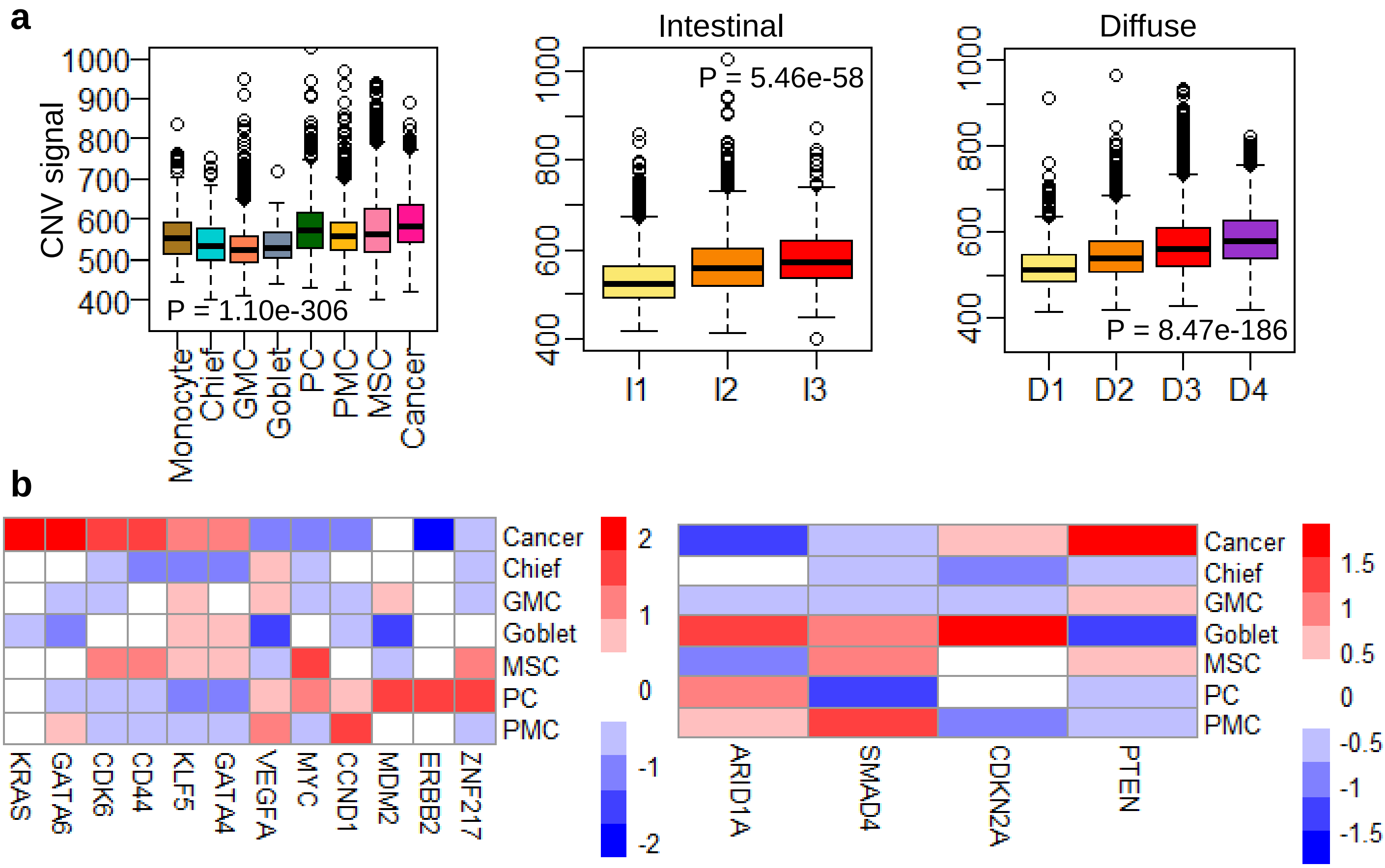

Supplementary Fig. 4. Copy number variations with gastric cell types. a CNV signals with epithelial cell types (left) and states from trajectory (middle and right panels). **b** CNV signals with annotated genes related amplification (left) and deletion (right) from TCGA STAD study, respectively.

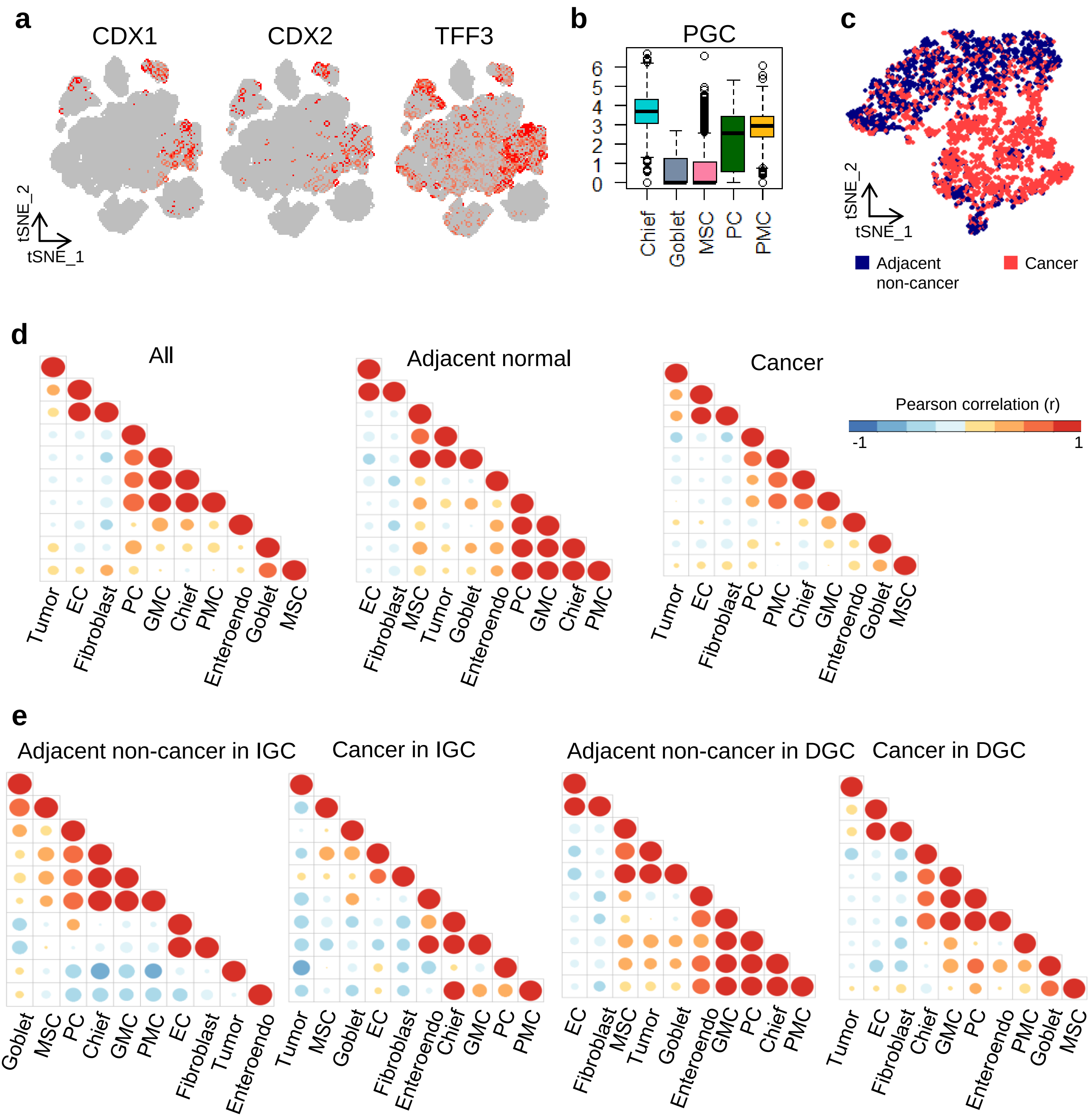

Supplementary Fig. 5. Cell type patterns for IM cluster and correlation with Lauren type. a t-SNE plot showing the expression of marker genes (*CDX1* and *CDX2*) of IM cell type. **b** Boxplot of *PGC* gene expression with IM cell types. **c** t-SNE plot representing distinct lesions in the IM sub-cluster. **d** Correlation matrix with 10 cell types according to malignancy. Colors represent correlation coefficient values calculated by the Pearson correlation test using the R software. **e** According to Lauren type with malignancy, separately.

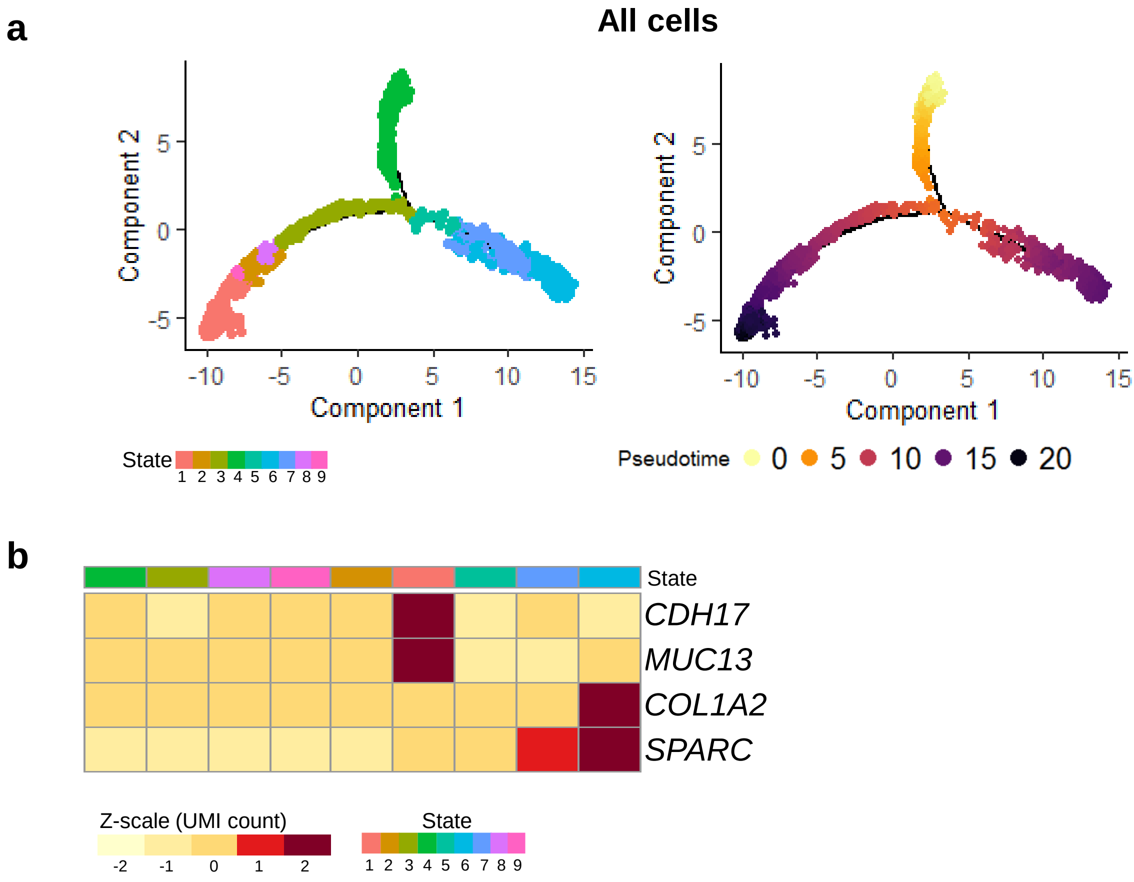

Supplementary Fig. 6. Characteristics of the pseudotemporal trajectory. a Pseudotemporal trajectory of all cells from adjacent non-cancer lesions and cancer lesions. Color of each cell represents annotated state (left) and pseudotime (right), determined using Monocle. **b** Expression patterns of known markers with states of the trajectory. CDH17 and MUC13 are markers of intestinal gastric cancer. COL1A2 and SPARC are known markers of diffuse gastric cancer.

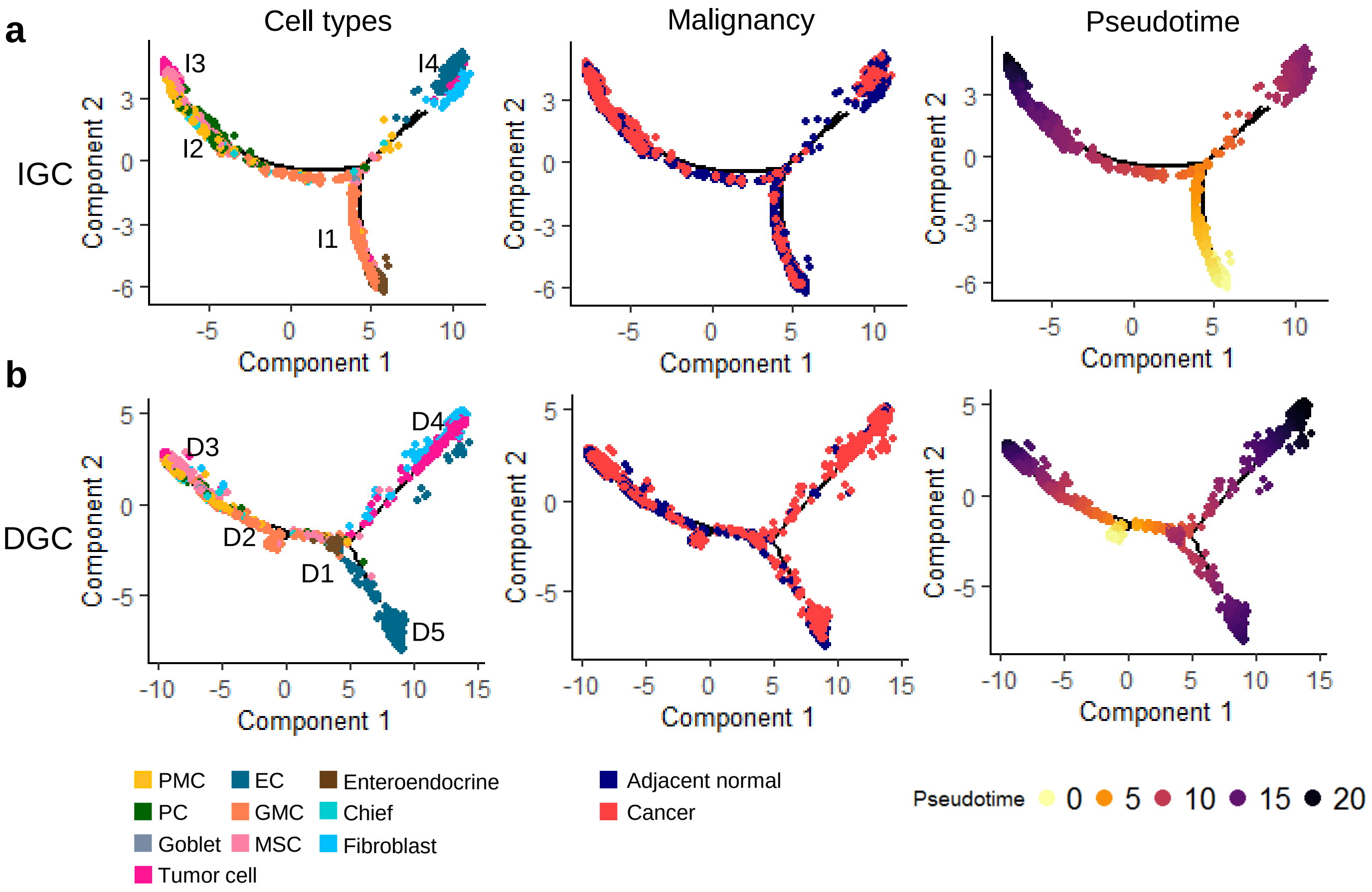

Supplementary Fig. 7. Pseudotemporal trajectory with Lauren type. a-b) Pseudotemporal trajectory with Lauren classification. Colors of cells represent cell type, malignancy, and pseudotime in respective plots.

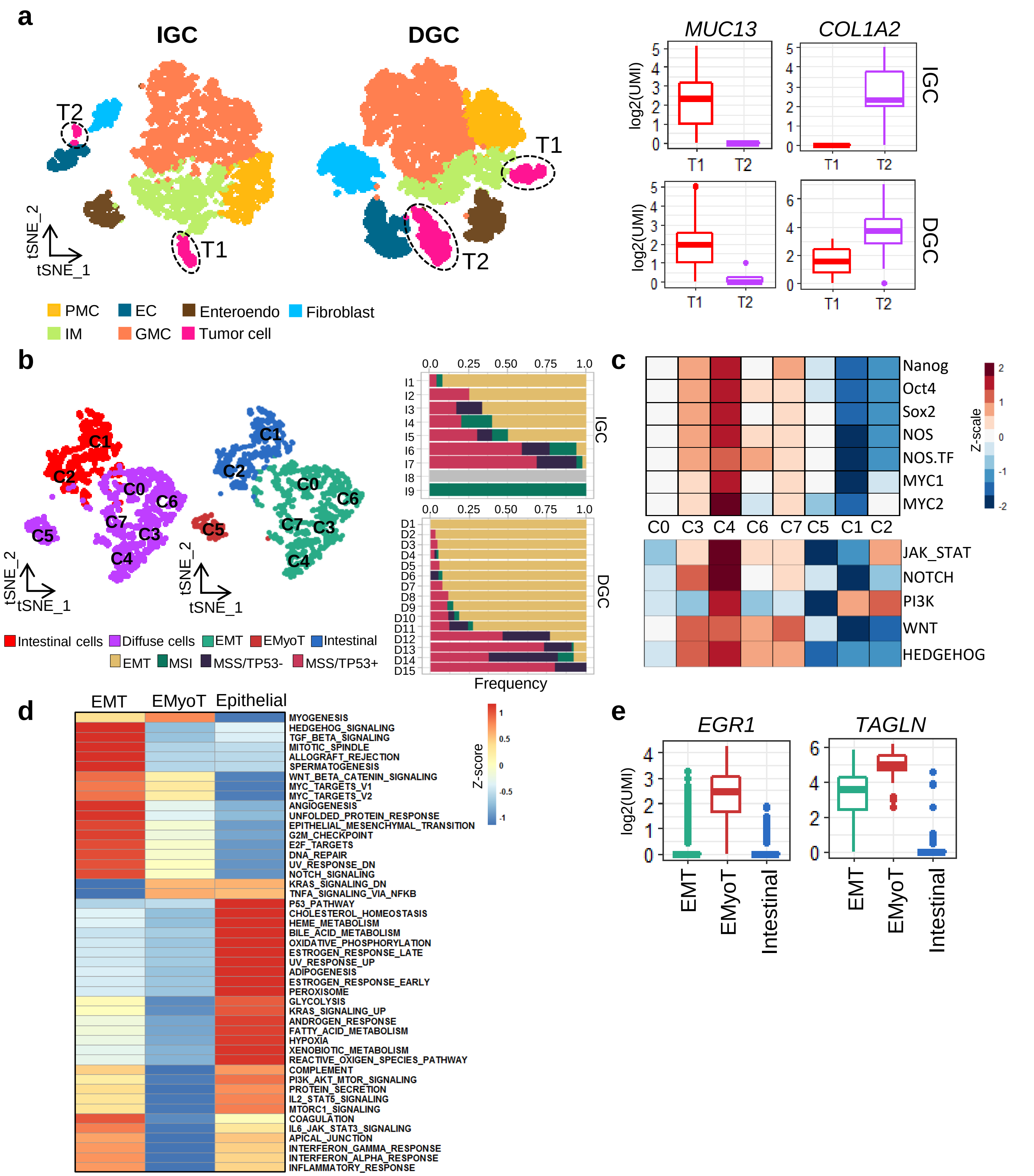

Supplementary Fig. 8. Defined tumor cell clusters and biological functional pathways with tumor sub-clusters. a t-Stochastic neighbor embedding (tSNE) plots with Lauren type, respectively (left). Box plots of known gastric cancer markers with separate sub-clusters of tumor cells (T1 and T2). **b** tSNE map of 1,003 tumor cells. Each cell represents a cell type mapped to two major cell types and three molecular types (left). Proportions of tumor cells with ACRG subtypes per patient and corresponding Lauren types (right). **c** Expression patterns of known signatures and pathways with eight tumor cell clusters. **d** Gene set enrichment analysis with cancer sub-clusters using hallmark pathways. **e)** Box plots of EMyoT markers (EGR1 and TAGLN) with cancer sub-clusters.

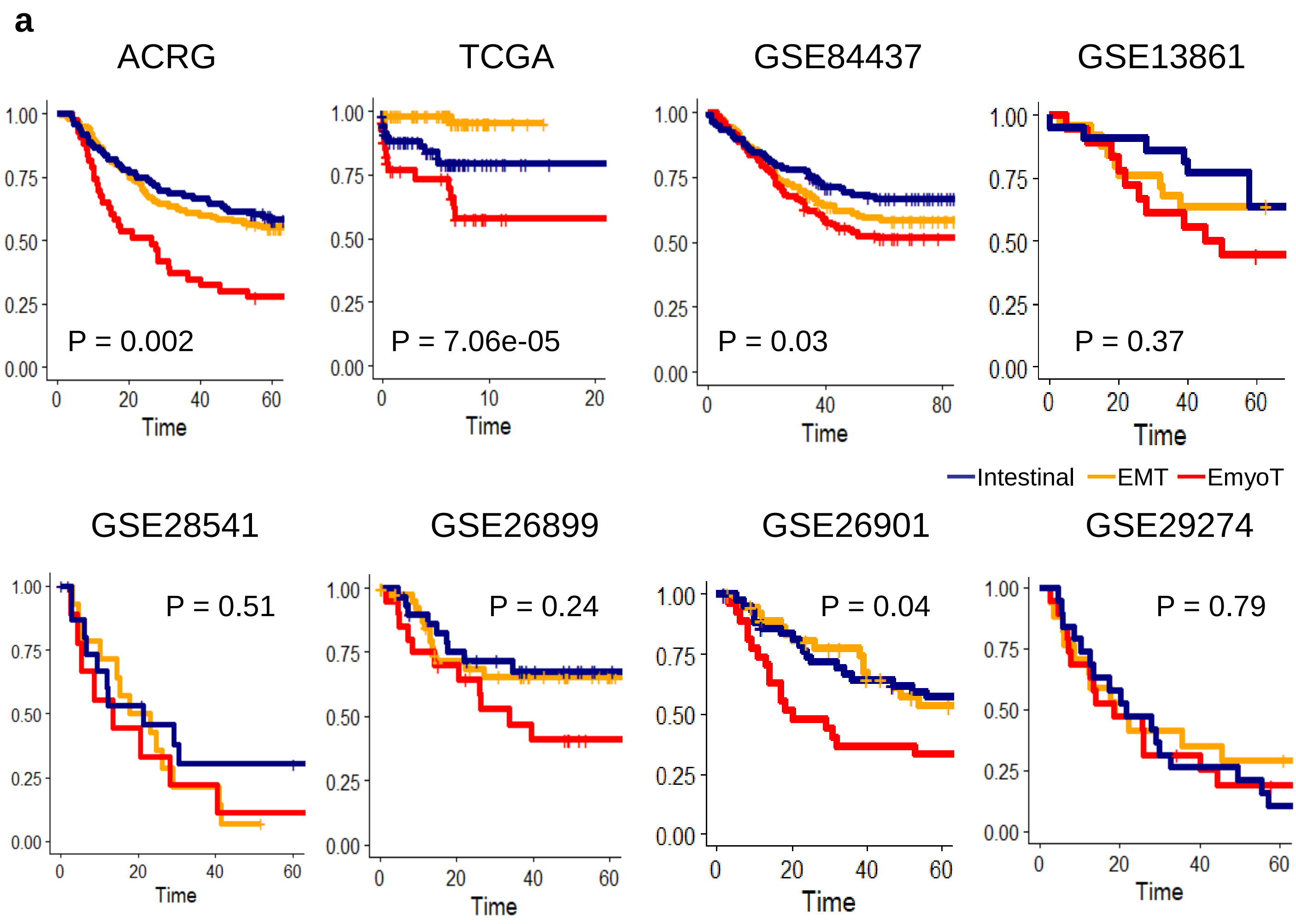

Supplementary Fig. 9. Survival analysis with cancer cells each independent cohort.

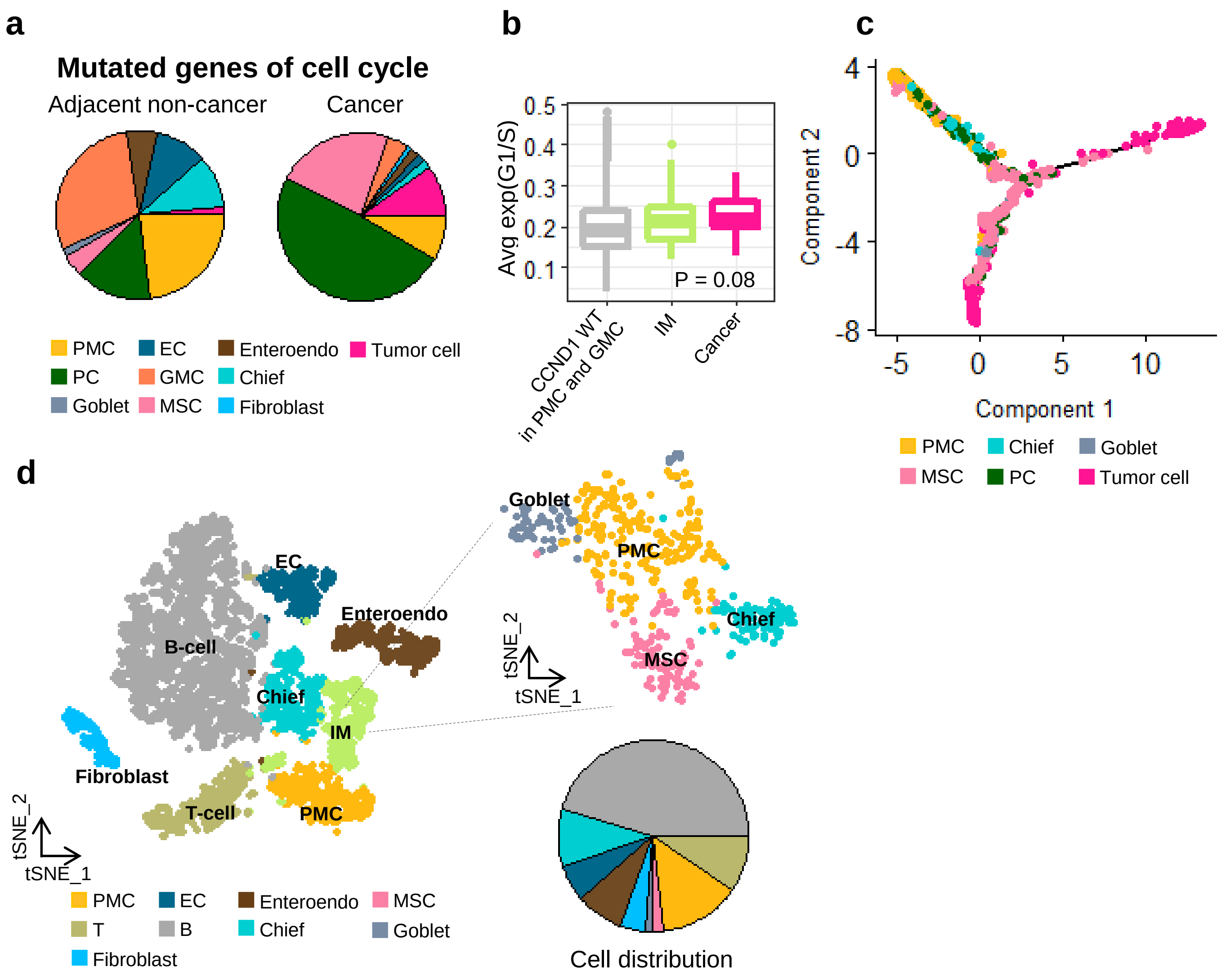

Supplementary Fig. 10. Cell cycle gene mutations and characteristics of hotspot mutations in the control. a Pie plots of the cell types with mutations of 8 cell cycle genes including CCND1 in adjacent non-cancer and cancer tissue, respectively. **b** Average gene expression levels of the G1/S phase pathway according to CCND1 mutation and cell type. P-values were calculated by analysis of variance (ANOVA) test. **c** Trajectory tree of premalignant (IM cells) and malignant cells. Each color represents a different cell type. **d** t-Stochastic neighbor embedding (tSNE) map of 4,813 cells in the control group diagnosed as having IM, but without GC for more than 5 years. Pie plot of the cell types in the control group.

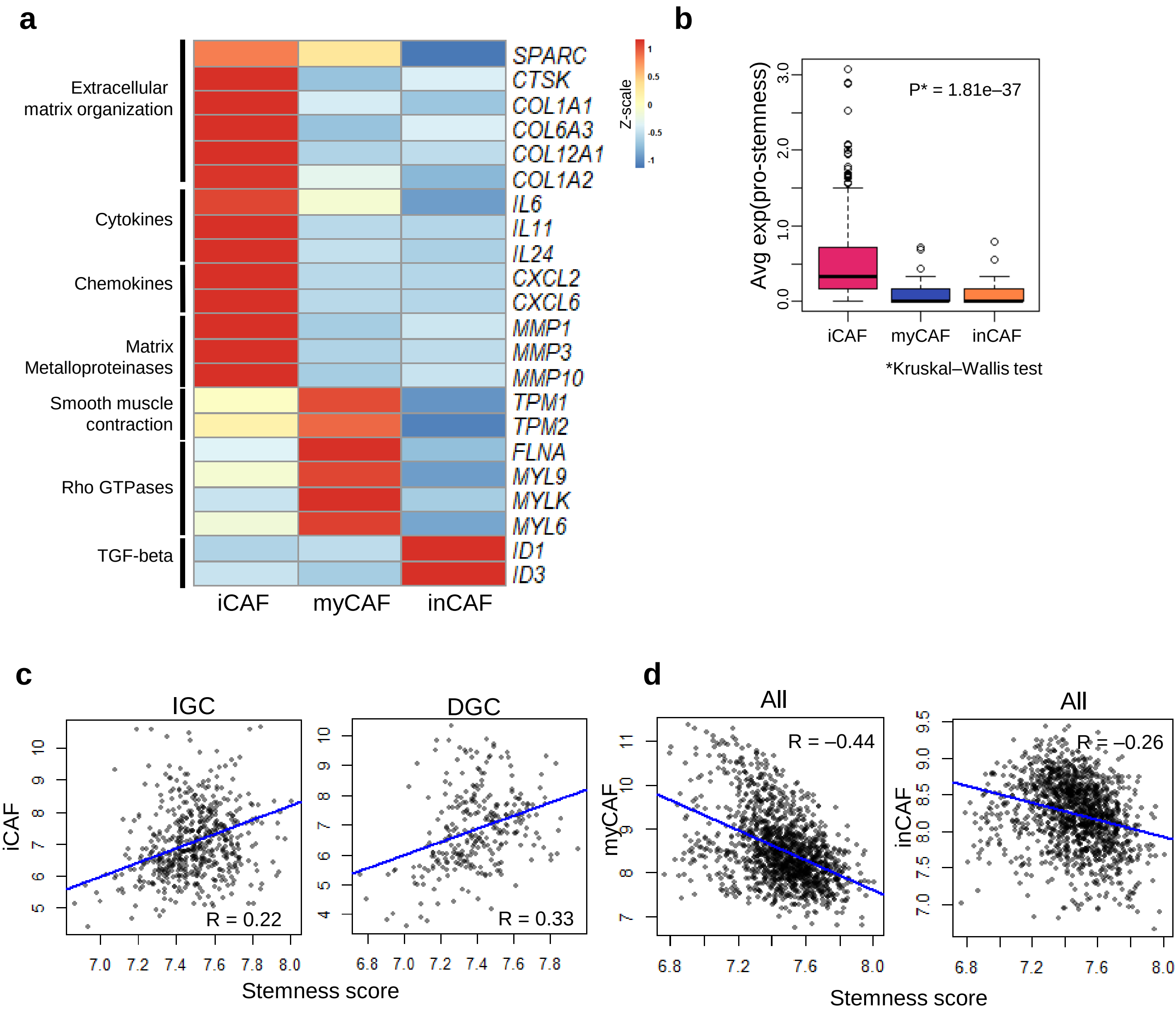

Supplementary Fig. 11. Differentially expressed genes in CAF subtypes and the relationship between CAF and stemness. a Heatmap of differentially expressed genes and pathways according to CAF subtypes. **b** Boxplot of pro-stemness-related genes for CAF subtypes. **c** Scatter plots between iCAF and stemness scores with Lauren type, respectively. R values were calculated by the glm test. **d** Patterns of stemness score and genes determined using myCAF and inCAF, respectively.

Supplementary Table 1. Clinical information

	Gender	Age	Diagnosis	Lauren	Atrophy	Cancer location*	*H.pylori*	MSI*	EBV*	#Cells (Adjacent noncancer)	#Cells (Cancer)
Patient01	M	75	EGC	Intestinal	N	LB, AW	+	MSS		80	91
Patient02	M	72	AGC	Diffuse	N	LB, GC	+	MSS	-	553	679
Patient03	F	66	EGC	Diffuse	Y	Antrum, GC, PW	-	MSS		260	409
Patient04	F	56	EGC	Diffuse	Y	Antrum, PW	+	MSS		236	244
Patient05	M	45	EGC	Diffuse	N	MB, LC	+	MSS		427	208
Patient06	M	58	EGC	Diffuse	N	UB, PW	Equivocal	MSS		1111	383
Patient07	M	76	EGC	Intestinal	Y	Angle, PW	+	MSS		275	111
Patient08	F	56	EGC	Diffuse	N	LB, LC	+	MSS		202	389
Patient09	M	55	EGC	Intestinal	Y	MB, LC	+	MSS		580	216
Patient10	M	54	EGC	Diffuse	N	LB, LC	+	MSS		154	208
Patient11	F	55	AGC	Diffuse	Y	MB, LC	+	MSS	-	170	162
Patient12	F	62	AGC	Diffuse	N	LB, LC	+	MSS	-	419	544
Patient13	M	70	EGC	Intestinal	N	Angle-antum, AW	+	MSS		317	155
Patient14	F	45	AGC	Diffuse	N	Antrum, PW	+	MSS	-	148	274
Patient15	M	66	AGC	Diffuse	N	MB, AW	-	MSS	-	442	269
Patient16	M	57	EGC	Diffuse	N	Angle, LC	Equivocal	MSS		84	331
Patient17	M	51	EGC	Intestinal	N	Antrum, AW	+	MSS		82	118
Patient18	M	58	EGC	Intestinal	N	Antrum, GC	+	MSS		401	203
Patient19	M	80	EGC	Intestinal	N	Angle, LC	+	MSS		83	163
Patient20	F	41	AGC	Diffuse	N	UB, LC	+	MSS		173	39
Patient21	M	63	AGC	Intestinal	Y	Antrum, LC	-	MSS		110	-
Patient22	M	46	EGC	Intestinal	Y	Antrum, PW	+	MSS		463	363

Patient23	M	76	AGC	Diffuse	Y	Angle, LC Antrum, AW	+	MSS		162	43
Patient24	F	44	AGC	Diffuse	N	LB, GC	+	MSS	+	163	325
Patient25	F	72	CAG with IM	-		-	+	-	-	253	
Patient26	F	59	CSG	-		-	+	-	-	479	
Patient27	F	61	CSG	-		-	+	-	-	2032	
Patient28	F	48	CSG	-		-	+	-	-	163	
Patient29	F	43	CAG with IM	-		-	+	-	-	1886	

*Abbreviation

EGC Early gastric cancer

AGC Advanced gastric cancer

CAG Chronic atrophic gastritis

IM Intestinal metaplasia

CSG Chronic superficial gastritis

LB Lower body

AW Anterior wall

GC Great Curvature

PW Post wall

MB Midbody

UB Upper body

LC Less curvature

MSS Microsatellite stability

MSI Microsatellite instability

\+ positive

\- negative

blank not tested

EBV	Epstein-Barr virus

Supplementary Table 2. DEG lists with cancer progression

DEGs	State	DEGs	State	DEGs	State	DEGs	State	DEGs	State	DEGs	State
C16orf89	I1	*FAM3D*	D1	*UBA52*	I2	*TAGLN2*	I2	*GPX2*	I2	*TUBB4B*	I2
C6orf58	I1	*PTMA*	I2	*UQCRH*	I2	*TXN*	I2	*H3F3A*	I2	*UBB*	I2
CLU	I1	*SNRPG*	I2	*ATP5B*	I2	*UQCRQ*	I2	*H3F3B*	I2	*UQCR10*	I2
CST3	I1	*TMSB10*	I2	*CDKN2A*	I2	*ATP5E*	I2	*HINT1*	I2	*UQCRB*	I2
LIPF	I1	*ATP5G3*	I2	*IFITM3*	I2	*ATP5G1*	I2	*HIST1H4C*	I2	*YBX1*	I2
MUC6	I1	*CYCS*	I2	*LY6E*	I2	*ATP5I*	I2	*HNRNPA2B1*	I2	*AKR1B10*	I2
PGC	I1	*EIF4A1*	I2	*MT1G*	I2	*ATP5J2*	I2	*HSP90AB1*	I2	*ANXA10*	I2
REG3A	I1	*ENO1*	I2	*MT1H*	I2	*ATP5L*	I2	*HSPA8*	I2	*CLIC1*	I2
LTF	I1	*GAPDH*	I2	*MT2A*	I2	*BTF3*	I2	*LDHA*	I2	*CLTB*	I2
LYZ	I1	*H2AFZ*	I2	*NACA*	I2	*C14orf2*	I2	*MT1E*	I2	*CTSE*	I2
PRR4	I1	*HMGB1*	I2	*PTGES3*	I2	*COX7B*	I2	*NPM1*	I2	*MUC5AC*	I2
REG1A	I1	*HMGN2*	I2	*TFF3*	I2	*COX7C*	I2	*OAZ1*	I2	*NQO1*	I2
ZG16B	I1	*HSP90AA1*	I2	*UBE2C*	I2	*COX8A*	I2	*PKM*	I2	*OCIAD2*	I2
BPIFB1	I1	*HSPE1*	I2	*ALDOA*	I2	*DBI*	I2	*PPIB*	I2	*PSME2*	I2
MSMB	I1	*PFN1*	I2	*CALM2*	I2	*EEF1A1*	I2	*SERF2*	I2	*TFF1*	I2
OLFM4	I1	*PPIA*	I2	*COX4I1*	I2	*EEF1B2*	I2	*SLIRP*	I2	*TMSB4X*	I2
GAST	D1	*PRDX1*	I2	*COX6B1*	I2	*EEF1D*	I2	*SPINK1*	I2	*AGR2*	I2
LCN2	D1	*PSMA7*	I2	*DYNLL1*	I2	*EIF1*	I2	*TMA7*	I2	*CA2*	I2
PRSS1	D1	*RAN*	I2	*GSTP1*	I2	*FABP5*	I2	*TPT1*	I2	*CYSTM1*	I2
PRSS3	D1	*TPI1*	I2	*NDUFA4*	I2	*FAU*	I2	*TSPO*	I2	*SEC61G*	I2
TFF2	D1	*TUBA1B*	I2	*RPL36AL*	I2	*GNB2L1*	I2	*TUBB*	I2	*ALDOB*	D2
ANPEP	D2	*KRT18*	D2	*S100P*	D2	*CES2*	I3	*MAL2*	I3	*TSPAN1*	I3
ANXA1	D2	*KRT19*	D2	*SDCBP2*	D2	*CFL1*	I3	*MALAT1*	I3	*TXNDC17*	I3
ANXA2	D2	*KRT20*	D2	*SH3BGRL3*	D2	*CLDN18*	I3	*MGST3*	I3	*VAMP8*	I3

APOA1	D2	*KRT7*	D2	*TM4SF1*	D2	*CLDN3*	I3	*MUC13*	I3	*GLRX*	I3
APOA4	D2	*KRT8*	D2	*TM4SF20*	D2	*CLDN4*	I3	*MYL12A*	I3	*PEPD*	I3
APOC3	D2	*LGALS3*	D2	*TM4SF4*	D2	*COX5B*	I3	*MYL12B*	I3	*PLS1*	I3
C15orf48	D2	*MTTP*	D2	*TMPRSS15*	D2	*COX6C*	I3	*MYL6*	I3	*DGAT1*	I3
CEACAM5	D2	*NEAT1*	D2	*TSPAN8*	D2	*COX7A2*	I3	*PHLDA2*	I3	*DHRS11*	I3
CEACAM6	D2	*PCK1*	D2	*LGALS4*	D2	*CSTB*	I3	*POLD4*	I3	*FUOM*	I3
CLDN7	D2	*PHGR1*	D2	*AGPAT2*	I3	*EDF1*	I3	*POMP*	I3	*KHK*	I3
CRIP1	D2	*PI3*	D2	*AGR3*	I3	*EPCAM*	I3	*PRR13*	I3	*TM4SF5*	I3
DPCR1	D2	*PLAC8*	D2	*ATP5J*	I3	*FTH1*	I3	*RARRES3*	I3	*CRYL1*	D3
FABP1	D2	*PRAP1*	D2	*B2M*	I3	*FTL*	I3	*RHOC*	I3	*CYBRD1*	D3
FABP2	D2	*PSCA*	D2	*C19orf33*	I3	*HLA-A*	I3	*SEPP1*	I3	*FAM3C*	D3
FXYD3	D2	*RBP2*	D2	*C19orf77*	I3	*HLA-B*	I3	*SERPINB6*	I3	*HIGD1A*	D3
GKN1	D2	*REG4*	D2	*CALM1*	I3	*HLA-C*	I3	*SFN*	I3	*HSD17B2*	D3
GKN2	D2	*S100A10*	D2	*CBR1*	I3	*HPGD*	I3	*SLPI*	I3	*MALL*	D3
HN1	D2	*S100A11*	D2	*CD55*	I3	*HRASLS2*	I3	*SMIM22*	I3	*MISP*	D3
IFI27	D2	*S100A14*	D2	*CD59*	I3	*HSPB1*	I3	*SRI*	I3	*OAT*	D3
IL32	D2	*S100A16*	D2	*CD63*	I3	*ISG15*	I3	*TACSTD2*	I3		
KLK10	D2	*S100A6*	D2	*CD9*	I3	*ISG20*	I3	*TMEM54*	I3		
EZR	D3	*CHN1*	D4	*GADD45B*	D4	*PTRF*	D4	*CALD1*	D4		
PERP	D3	*CNN3*	D4	*GPX3*	D4	*RERGL*	D4	*CAV1*	D4		
RAC1	D3	*COL15A1*	D4	*IFITM2*	D4	*RGS16*	D4	*CD36*	D4		
AKR1C3	D3	*COL18A1*	D4	*IGFBP7*	D4	*RGS5*	D4	*EMP3*	D4		
ARPC1B	D3	*COL1A1*	D4	*ITGB1*	D4	*S100A4*	D4	*FN1*	D4		
CALR	D3	*COL1A2*	D4	*JUNB*	D4	*SDC2*	D4	*FRZB*	D4		
COX6A1	D3	*COL3A1*	D4	*LGALS1*	D4	*SELM*	D4	*PPP1R14A*	D4		
GSTA1	D3	*COL4A1*	D4	*LMNA*	D4	*SERPINH1*	D4	*PRKCDBP*	D4		

HSP90B1	D3	*COL4A2*	D4	*LUM*	D4	*SOD3*	D4	*PRSS23*	D4		
MMP7	D3	*COL5A2*	D4	*MFGE8*	D4	*SPARC*	D4	*TUBA1A*	D4		
NDUFA1	D3	*COL6A1*	D4	*MGP*	D4	*SPARCL1*	D4	*VIM*	D4		
SPRR1B	D3	*COL6A2*	D4	*MYH11*	D4	*SPON2*	D4	*ARHGDIB*	D4		
TCEB2	D3	*CRIP2*	D4	*MYL9*	D4	*TAGLN*	D4	*BGN*	D4		
UQCR11	D3	*CSRP2*	D4	*MYLK*	D4	*TFPI*	D4				
USMG5	D3	*CTSC*	D4	*NDUFA4L2*	D4	*THY1*	D4				
YWHAZ	D3	*DCN*	D4	*PI15*	D4	*TIMP1*	D4				
PCK2	D3	*DSTN*	D4	*PLN*	D4	*TPM1*	D4				
SULT1A1	D3	*EID1*	D4	*POSTN*	D4	*TPM2*	D4				

Supplementary Table 3. DEG lists with cancer sub-clusters

cluster	gene	cluster	gene	cluster	gene	cluster	gene	cluster	gene	cluster	gene
Intestinal	*PHGR1*	Intestinal	*APOA1*	Intestinal	*SEPP1*	Intestinal	*REEP6*	Intestinal	*ALPI*	Intestinal	*OAT*
Intestinal	*RBP2*	Intestinal	*APOA4*	Intestinal	*SULT1A2*	Intestinal	*CLDN4*	Intestinal	*AOC1*	Intestinal	*MDK*
Intestinal	*C19orf77*	Intestinal	*KHK*	Intestinal	*FXYD3*	Intestinal	*VIL1*	Intestinal	*GLRX*	Intestinal	*HEBP1*
Intestinal	*ANPEP*	Intestinal	*C11orf86*	Intestinal	*EPCAM*	Intestinal	*VAMP8*	Intestinal	*TM4SF4*	Intestinal	*C8G*
Intestinal	*ALDOB*	Intestinal	*HSD17B2*	Intestinal	*TSPAN8*	Intestinal	*CRYL1*	Intestinal	*HPGD*	Intestinal	*SAT2*
Intestinal	*PRAP1*	Intestinal	*TM4SF20*	Intestinal	*CYB5A*	Intestinal	*SCP2*	Intestinal	*CDHR2*	Intestinal	*CCL25*
Intestinal	*FABP2*	Intestinal	*PCK1*	Intestinal	*FBP1*	Intestinal	*MISP*	Intestinal	*PLS1*	Intestinal	*HLA-DRB1*
Intestinal	*MTTP*	Intestinal	*GSTA1*	Intestinal	*PPP1R14D*	Intestinal	*CHP2*	Intestinal	*PLAC8*	Intestinal	*CTSE*
Intestinal	*FABP1*	Intestinal	*DGAT1*	Intestinal	*LGALS3*	Intestinal	*DHRS11*	Intestinal	*SERPINA1*	Intestinal	*DPEP1*
Intestinal	*CDHR5*	Intestinal	*MYO1A*	Intestinal	*AGPAT2*	Intestinal	*FUOM*	Intestinal	*MPST*	Intestinal	*HLA-DRA*
Intestinal	*CBR1*	Intestinal	*LGALS4*	Intestinal	*C19orf33*	Intestinal	*CIDEC*	Intestinal	*CYP3A4*	Intestinal	*ENPP7*
Intestinal	*CREB3L3*	Intestinal	*SMIM22*	Intestinal	*ESPN*	Intestinal	*ELF3*	Intestinal	*MYO15B*	Intestinal	*CD74*
Intestinal	*APOB*	Intestinal	*PRR13*	Intestinal	*SULT1A1*	Intestinal	*SFN*	Intestinal	*ABCG2*	Intestinal	*ATP1B1*
Intestinal	*PIGR*	Intestinal	*MALL*	Intestinal	*CLDN7*	Intestinal	*ALDH1A1*	Intestinal	*AADAC*	Intestinal	*CDKN2B-AS1*
Intestinal	*TM4SF5*	Intestinal	*CLDN3*	Intestinal	*PRSS3*	Intestinal	*MUC13*	Intestinal	*CDH17*	Intestinal	*UGT2B17*
Intestinal	*CES2*	Intestinal	*AKR1B10*	Intestinal	*TMPRSS15*	Intestinal	*CLDN15*	Intestinal	*C2orf88*	Intestinal	*REG1A*
Intestinal	*KRT8*	Intestinal	*KRT20*	Intestinal	*FAM3C*	Intestinal	*SI*	Intestinal	*HRASLS2*	Intestinal	*S100G*
Intestinal	*PCK2*	Intestinal	*CIDEB*	Intestinal	*S100A10*	Intestinal	*FAM3B*	Intestinal	*AGR3*	Intestinal	*REG1B*
Intestinal	*AKR7A3*	Intestinal	*APOC3*	Intestinal	*PEBP1*	Intestinal	*CYP3A5*	Intestinal	*PEPD*	Intestinal	*AGR2*
Intestinal	*S100P*	Intestinal	*ANXA10*	Intestinal	*CTSE*	Intestinal	*OLFM4*	EmyoT	*JUN*	EmyoT	*EIF4A2*
Intestinal	*TFF1*	Intestinal	*S100A10*	Intestinal	*KRT7*	Intestinal	*FCGBP*	EmyoT	*PLN*	EmyoT	*CKB*
Intestinal	*TSPAN1*	Intestinal	*S100A14*	Intestinal	*PI3*	Intestinal	*SPINK4*	EmyoT	*GADD45B*	EmyoT	*IGFBP5*
Intestinal	*TSPAN8*	Intestinal	*PRSS3*	Intestinal	*LYZ*	Intestinal	*MUC2*	EmyoT	*EGR1*	EmyoT	*FRZB*

Intestinal	*REG4*	Intestinal	*CLDN4*	Intestinal	*TACSTD2*	Intestinal	*MT1G*	EmyoT	*BCAM*	EmyoT	*MLTK*
Intestinal	*S100A6*	Intestinal	*CLDN18*	Intestinal	*TFF2*	Intestinal	*ITLN1*	EmyoT	*FOS*	EmyoT	*RHOB*
Intestinal	*CEACAM5*	Intestinal	*SDCBP2*	Intestinal	*CEACAM6*	Intestinal	*REG1A*	EmyoT	*MYL9*	EmyoT	*RCAN2*
Intestinal	*FXYD3*	Intestinal	*SMIM22*	Intestinal	*SERPINB1*	Intestinal	*ZG16*	EmyoT	*SORBS2*	EmyoT	*FLNA*
Intestinal	*KRT18*	Intestinal	*MAL2*	Intestinal	*TXN*	Intestinal	*MMP1*	EmyoT	*TSC22D1*	EmyoT	*C11orf96*
Intestinal	*GPRC5A*	Intestinal	*C19orf33*	Intestinal	*VSIG2*	EMT	*FN1*	EmyoT	*TAGLN*	EmyoT	*CNN1*
Intestinal	*SLPI*	Intestinal	*PLAC8*	Intestinal	*LMO7*	EMT	*HIST1H4C*	EmyoT	*LBH*	EmyoT	*KLF2*
Intestinal	*KRT19*	Intestinal	*CYSTM1*	Intestinal	*TM4SF20*	EMT	*TUBA1B*	EmyoT	*NET1*	EmyoT	*SOCS3*
Intestinal	*SH3BGRL3*	Intestinal	*OCIAD2*	Intestinal	*MUC13*	EMT	*H2AFZ*	EmyoT	*TPM2*	EmyoT	*BTG2*
Intestinal	*KRT8*	Intestinal	*AGR3*	Intestinal	*SPINK1*	EMT	*STMN1*	EmyoT	*ZFP36*	EmyoT	*MFAP4*
Intestinal	*TFF3*	Intestinal	*LGALS3*	Intestinal	*TMEM54*	EMT	*UBE2C*	EmyoT	*MAP3K7CL*	EmyoT	*GBP2*
Intestinal	*EPCAM*	Intestinal	*SFN*	Intestinal	*LCN2*	EmyoT	*RERGL*	EmyoT	*SNCG*	EmyoT	*ARID5A*
Intestinal	*CD55*	Intestinal	*MUC1*	Intestinal	*PSCA*	EmyoT	*MYH11*	EmyoT	*PPP1R15A*	EmyoT	*MFGE8*
Intestinal	*LGALS4*	Intestinal	*PERP*	Intestinal	*MUC5AC*	EmyoT	*DSTN*	EmyoT	*CIRBP*	EmyoT	*SOD3*
Intestinal	*TM4SF1*	Intestinal	*GPX2*	Intestinal	*C15orf48*	EmyoT	*IER2*	EmyoT	*SPARCL1*	EmyoT	*CSRP1*
Intestinal	*S100A16*	Intestinal	*HN1*	Intestinal	*PDZK1IP1*	EmyoT	*JUNB*	EmyoT	*CSRP2*	EmyoT	*FHL1*
EmyoT	*MYLK*	EmyoT	*C10orf10*	EmyoT	*LIPF*	EMT	*COL3A1*	EMT	*FN1*	EMT	*RGS5*
EmyoT	*NUPR1*	EmyoT	*DES*	EMT	*COL4A1*	EMT	*COL1A1*	EMT	*IL8*	EMT	*IGJ*
EmyoT	*SERPINI1*	EmyoT	*CYR61*	EMT	*COL1A2*	EMT	*S100A4*	EMT	*COL15A1*	EMT	*FABP4*

Supplementary Table 4. Known gene sigatures with previous studies

EMyoT	EMT	Cytokine	P53	MSI	ES exp	Nanog targets	Oct4 targets	Sox2 targets	EBV
SRF	*ADAM23*	*CCL18*	*MDM2*	*ADAM10*	*ACTA1*	*ABCB7*	*ADD3*	*ABCB7*	*HLA-A*
MKL	*ADAMTS1*	*CCL19*	*CDKN1A*	*AK2*	*ACTC1*	*ACADM*	*AUH*	*ACO2*	*HLA-B*
CNN1	*AFF3*	*CCL2*		*AMFR*	*ACTN3*	*ACAT2*	*BMP7*	*ACOX1*	*HLA-C*
TAGLN	*AK5*	*CCL21*		*ANP32E*	*ADD2*	*ACO2*	*ZFP36L1*	*ADAR*	*HLA-DMB*
SMAD3	*AKAP12*	*CCL3*		*ARL6IP1*	*PARP1*	*ADAR*	*KLF5*	*ADD3*	*HLA-DPA1*
MRTF	*ALPK2*	*CCL4*		*ARNTL2*	*ALPL*	*ADD3*	*BUB1B*	*ADFP*	*HLA-DPB1*
ZEB1	*ANGPTL2*	*CCL5*		*ASPHD2*	*AMD1*	*ADFP*	*CA2*	*ADRBK2*	*HLA-DQA1*
	ANKRD1	*CCL8*		*ATP1B1*	*BIRC5*	*ADRBK2*	*CA4*	*AP2A1*	*HLA-DQB1*
	ANTXR1	*CXCL10*		*ATP5A1*	*ATP1A2*	*AP2A1*	*CACNA2D1*	*ALCAM*	*HLA-DRA*
	ANXA6	*CXCL11*		*ATP6V1B2*	*BMPR1A*	*AP1G1*	*CAPZA2*	*ALPL*	*HLA-DRB1*
	AOX1	*CXCL13*		*B3GNT4*	*BUB1*	*AK3L1*	*ENTPD1*	*APEX1*	*HLA-DRB5*
	AP1S2	*CXCL9*		*BNIP3L*	*BUB1B*	*ALCAM*	*CDH1*	*APP*	*HLA-F*
	ARMCX1			*C12orf23*	*C1QBP*	*ANGPT1*	*COL12A1*	*AQP2*	
	ATP8B2			*C12orf57*	*CASP3*	*ANXA1*	*CPT1A*	*ARF3*	
	ATP8B3			*C18orf21*	*CBS*	*APLP2*	*CTGF*	*ARF4*	
	AXL			*C18orf24*	*CCNA2*	*APOA2*	*DPYSL2*	*ARL4D*	
	BDNF			*C18orf25*	*CCNB1*	*APOB*	*DPYSL3*	*ARHGAP1*	
	BICC1			*C18orf55*	*CD24*	*AQP2*	*DTNA*	*ASNA1*	
	BNC2			*C19orf51*	*CDC2*	*ARF3*	*DUSP6*	*ATF3*	
	BVES			*C1orf149*	*CDC6*	*ARF4*	*EIF2S1*	*ATF4*	
	C10orf38			*C4orf27*	*CDC20*	*ARHGAP1*	*ELAVL2*	*ALDH7A1*	
	C10orf56			*C9orf30*	*CDC25A*	*ASAH1*	*EPHA1*	*ATP5F1*	
	C16orf45			*CAMK2N2*	*CTSC*	*ATF4*	*FGF2*	*AUH*	
	C1S			*CCDC109B*	*CHEK1*	*ATP5F1*	*FGFR1*	*BCAT1*	

C9orf19	*CCDC5*	*CRABP1*	*ATP6V1A*	*FGFR2*	*BCKDHA*
CAP2	*CCL7*	*CRABP2*	*B2M*	*FOXO1*	*CCND1*
CCL2	*CCL8*	*CRMP1*	*BCAT1*	*FUS*	*BCL9*
CDH11	*CD68*	*CSE1L*	*BCKDHA*	*GAP43*	*BMP2*
CDH2	*CDC42SE1*	*CXADR*	*BCL9*	*GAS1*	*BMP7*
CDH4	*CDCA2*	*CYP26A1*	*BMP2*	*GATA6*	*ZFP36L1*
CHN1	*CDK2*	*COCH*	*BMP7*	*GJA1*	*KLF5*
CLDN11	*CNOT7*	*DHFR*	*POLR3D*	*GPC3*	*BTG1*
CLIP3	*COPG*	*DIAPH2*	*BNIP1*	*GLDC*	*BUB1B*
CMTM3	*COPS3*	*DLG3*	*ZFP36L1*	*GNG10*	*CA2*
COL12A1	*CSNK1G1*	*DNA2L*	*KLF5*	*GPS1*	*CA4*
COL1A2	*CYB5D1*	*DNMT3A*	*BUB1B*	*GRID2*	*CACNA1A*
COL3A1	*DCAKD*	*DNMT3B*	*CA2*	*HAS2*	*CACNA2D1*
COL4A1	*DCUN1D1*	*DSG2*	*CA4*	*HHEX*	*CALM2*
COL5A1	*DOCK5*	*ECT2*	*CACNA1A*	*HMGB2*	*CALR*
COL5A2	*DUSP4*	*SLC29A1*	*CACNA2D1*	*HOXB5*	*CAPZA2*
COL6A1	*EXOSC9*	*EPHA1*	*CALB1*	*TNC*	*CASP9*
CPA4	*FAM151B*	*EPRS*	*CALD1*	*ID2*	*CDC2*
CTGF	*FAM18B*	*ERBB2*	*CALM2*	*IFI16*	*CDH2*
CYBRD1	*FECH*	*ETV1*	*CALR*	*JARID2*	*CDH3*
DAB2	*FNTB*	*ETV4*	*CAPZA2*	*JUND*	*CDK6*
DFNA5	*FUT8*	*FABP5*	*CASP9*	*JUP*	*CETN3*
DIO2	*GNLY*	*FEN1*	*CAV1*	*KCNN2*	*CFL1*
DKK3	*GPR126*	*GPC4*	*RUNX1T1*	*KDR*	*FOXN3*
DLC1	*GTF2A2*	*FGF2*	*SERPINH1*	*LAMA4*	*CLN3*
DOCK10	*HBS1L*	*FGF13*	*ENTPD1*	*MAN2C1*	*COL12A1*
DPYSL3	*HNRNPL*	*FGFR1*	*CDC2*	*MCC*	*COPB1*

EDIL3	*HPSE*	*FKBP5*	*CDH1*	*MEIS2*	*CPT1A*
ELOVL2	*INSR*	*FOXO1*	*CDH2*	*MAP3K3*	*CSNK1E*
EML1	*IQGAP3*	*GABRA5*	*CDK6*	*MMP2*	*VCAN*
EMP3	*KCNRG*	*GABRB3*	*CETN3*	*MTM1*	*CTGF*
EPB41L5	*KCTD9*	*GAD1*	*CFL1*	*NCBP1*	*CTH*
EPDR1	*KDELR3*	*GART*	*CHD2*	*NEFM*	*DDX5*
EVI2A	*KDSR*	*GJA1*	*FOXN3*	*NEFL*	*DNM2*
F2R	*KIR2DL4*	*GLDC*	*CKS2*	*ROR1*	*DPAGT1*
FAM101B	*KLRD1*	*GPM6B*	*CLIC1*	*ORC1L*	*DPYSL2*
FAT4	*KPNA1*	*GPR19*	*CLN3*	*PAK1*	*DPYSL3*
FBN1	*KRT7*	*MSH6*	*CNN2*	*PDCL*	*DTNA*
FGF2	*LRRC16A*	*HAS3*	*CNN3*	*PCTK2*	*DUSP6*
FGF5	*LYG1*	*HELLS*	*COL4A5*	*ENPP2*	*DVL2*
FGFR1	*MAP3K6*	*HMGB3*	*COL4A6*	*PFTK1*	*E2F3*
FHL1	*MBP*	*HMGA1*	*COL7A1*	*EXOSC9*	*EEF2*
FLRT2	*ME2*	*HMMR*	*COL12A1*	*POU5F1*	*EGR3*
FSTL1	*MFAP1*	*HNRPAB*	*COX6A1*	*PPP2R1B*	*EIF4G2*
GFPT2	*MIB1*	*HSPA4*	*CPT1A*	*PPP2R3A*	*ELAVL2*
GLIPR1	*MSH4*	*HSPA8*	*CRYZ*	*PRPS1*	*ENSA*
GLT25D2	*MT1X*	*HSPD1*	*CS*	*PSMA3*	*EPHA1*
GNB4	*MT2A*	*ILF3*	*MAPK14*	*PTPN2*	*ERBB2*
GNG11	*MTA2*	*INDO*	*CSNK1E*	*RAB5A*	*FANCC*
GPC6	*NAP1L4*	*ITPR3*	*VCAN*	*RAD51C*	*FANCF*
GPR176	*NARS*	*JARID2*	*CSTF1*	*RASGRF2*	*FARSA*
GREM1	*NUCB2*	*KAL1*	*CSTF3*	*REST*	*FGF2*
HAS2	*NUTF2*	*KCNS3*	*CTGF*	*RFNG*	*FGFR1*
HEG1	*PAFAH1B2*	*KIF5C*	*CTSL2*	*RPL32*	*FGFR2*

HS3ST3A1	*PBK*	*KLKB1*	*CXADR*	*RPS3A*	*FOXO1*
HTRA1	*PGGT1B*	*KPNA2*	*CYLD*	*RPS18*	*FTL*
IGFBP7	*PHF5A*	*KRT8*	*CYP1B1*	*VPS52*	*FUS*
IL13RA2	*PIAS2*	*LCK*	*DCX*	*SALL1*	*FZD2*
JAM3	*PLAA*	*LGALS8*	*DDX5*	*SET*	*GJA1*
KIRREL	*POLDIP3*	*TACSTD1*	*DMXL1*	*SFRP1*	*GNAI1*
LAMA4	*PPIB*	*M6PR*	*DHCR7*	*SFRP2*	*GNG10*
LEPREL1	*PPM1A*	*MAN2A1*	*DNM2*	*SFRS4*	*GRK6*
LGALS1	*PPP1R8*	*MARS*	*DPAGT1*	*SKIL*	*GRID2*
LHFP	*PPP4R1*	*MAT2A*	*DPYSL2*	*SNRPN*	*GSK3A*
LIX1L	*PRPF39*	*MCM2*	*DPYSL3*	*SOX2*	*GSK3B*
LOX	*PSIP1*	*MCM3*	*DTNA*	*STAT3*	*H2AFX*
MAP1B	*PTPN2*	*MCM4*	*DUSP6*	*TAF12*	*H3F3B*
MMP2	*RAB27B*	*MCM5*	*DVL2*	*TAL1*	*HAS2*
MRAS	*RAC3*	*MCM6*	*E2F3*	*TALDO1*	*HDAC2*
MSRB3	*RAP2B*	*MCM7*	*EEF2*	*TCF4*	*HELLS*
NAP1L3	*RNF19B*	*MFGE8*	*EIF4B*	*TCF12*	*HHEX*
NAV3	*RNF215*	*MGST1*	*EIF4G2*	*TCF20*	*HMOX1*
NDN	*RPL22L1*	*MICB*	*ENSA*	*TDGF1*	*HNRNPA1*
NEGR1	*RPRD1A*	*MRE11A*	*EPHA1*	*NR2F2*	*HNRNPA2B1*
NEXN	*RPS29*	*MSH2*	*ERBB2*	*LEFTY2*	*HNRNPC*
NID1	*RTTN*	*NUDT1*	*ERCC1*	*THBS2*	*HNRPK*
NRG1	*SAP30*	*MTHFD1*	*ERH*	*TLE3*	*HNRNPL*
NUDT11	*SEC22B*	*NASP*	*EXTL2*	*TOP2A*	*HSP90AB1*
PAPPA	*SERPINB8*	*NFYB*	*FANCA*	*TRPS1*	*TNC*
PDE7B	*SETD5*	*NODAL*	*ACSL4*	*UBE2D3*	*ID1*
PLAGL1	*SFRS6*	*NPM1*	*FANCF*	*WEE1*	*IDH3G*

PMP22	*SFXN1*	*NTHL1*	*FBLN1*	*ZIC1*	*IFI16*
PNMA2	*SGPP1*	*NTS*	*FARSA*	*ZIC2*	*IGFBP2*
POPDC3	*SLC25A37*	*OAZ2*	*FAT*	*ZIC3*	*CYR61*
POSTN	*SLC2A5*	*ORC1L*	*FDPS*	*USP7*	*IK*
PRKD1	*SLC35A1*	*PAK1*	*GPC4*	*MYST3*	*ILF2*
PRR16	*SMAD2*	*PCDH1*	*FGF2*	*MLLT10*	*ILF3*
PTGIS	*SMAD4*	*PDCD2*	*FGFR1*	*CDC7*	*INHBA*
PTRF	*SMCHD1*	*PDK1*	*FGFR2*	*EOMES*	*JARID2*
PTX3	*SMURF2*	*PFAS*	*FHIT*	*HIST2H2BE*	*JUP*
RBM24	*SOCS6*	*PIK3CB*	*FKBP1B*	*PARG*	*KCNN2*
RBMS3	*SSR1*	*PLCB3*	*FOXO1*	*CDC14B*	*KDR*
RBPMS2	*STT3A*	*PMAIP1*	*FOXO3*	*KLF7*	*KIF11*
RECK	*STYX*	*EXOSC9*	*FUS*	*PPAP2A*	*KPNA3*
RFTN1	*TFAP2A*	*PNN*	*FZD2*	*B3GALT4*	*LAMA4*
SERPINE1	*THEX1*	*PODXL*	*GALK2*	*TRIM24*	*LASP1*
SIRPA	*THRAP3*	*POLE2*	*GANC*	*HESX1*	*ABLIM1*
SLC2A3	*TIPIN*	*POU5F1*	*GART*	*ALKBH1*	*LOH11CR2A*
SLC47A1	*TMEM107*	*PPM1B*	*GATA6*	*TSC22D1*	*LOXL2*
SPARC	*TNFSF9*	*PPP2R1B*	*GJA1*	*CDC123*	*LRP2*
SRGN	*TNNT1*	*PPP2R2B*	*GLA*	*SPAG9*	*CAPRIN1*
SRPX	*TNPO1*	*PRIM1*	*GLG1*	*BUB3*	*NBR1*
ST3GAL2	*TOMM22*	*PRIM2*	*GLUD1*	*LARGE*	*SMAD3*
SUSD5	*TRIM7*	*PRKX*	*GNAI1*	*LRAT*	*MAN2C1*
SYDE1	*TSPAN14*	*PRPS1*	*GNG10*	*MSC*	*MLH1*
TBXA2R	*TWSG1*	*PTPN2*	*GOLGA4*	*DHRS3*	*MAP3K11*
TCF4	*TXNDC1*	*PTPRZ1*	*GSK3A*	*TRIP4*	*MOBP*
TGFB2	*TXNDC10*	*RAB3B*	*GSTT2*	*GTF3C4*	*MTM1*

TMEM158	*TXNDC9*	*RARRES2*	*GTF2E1*	*PLAA*	*MYO9A*
TMEM47	*TXNL1*	*RBBP8*	*GTF2H2*	*CDYL*	*NCBP1*
TMSL8	*TXNL4A*	*RFC3*	*GYPC*	*BAG5*	*NDUFA2*
TNFRSF19	*UBE2M*	*RFC4*	*H2AFX*	*ATP6V1G1*	*NDUFB5*
TRPA1	*USF1*	*ABCE1*	*H2AFZ*	*GTPBP1*	*NDUFB8*
TTC28	*USP14*	*ROBO1*	*HIST1H2BD*	*NFE2L3*	*NIT1*
TTLL7	*VAPA*	*RPS24*	*HAS2*	*FEZ1*	*NKTR*
TUB	*WDR41*	*RRM2*	*HELLS*	*KIAA0101*	*NOTCH1*
TUBA1A	*WDR43*	*SALL2*	*HHEX*	*KIAA0174*	*NP*
UCHL1	*WDR57*	*SALL1*	*HNRNPA1*	*ZEB2*	*ROR1*
VIM	*WDR76*	*SCNN1A*	*HNRNPA2B1*	*SUPT7L*	*OAZ2*
WIPF1	*ZCCHC2*	*SFRP1*	*HNRPH2*	*JOSD1*	*ORC1L*
WNT5B		*SFRP2*	*HOXB5*	*MED12*	*OXA1L*
ZEB1		*SFRS1*	*HSPA4*	*NAALAD2*	*PRDX1*
ZEB2		*SFRS7*	*HSPA5*	*ARPC5*	*PAK1*
ZFPM2		*ST6GAL1*	*TNC*	*ZMPSTE24*	*PDCL*
ZNF788		*SLC6A8*	*ID1*	*TRIM22*	*PCBP1*
		SLC16A1	*ID2*	*HMG20A*	*PCSK5*
		SMS	*IDH3A*	*TIMM23*	*PCTK1*
		SNRPA	*IFI16*	*NEBL*	*PCTK2*
		SNRPN	*IGFBP2*	*SORBS1*	*ENPP2*
		SORL1	*IGFBP3*	*POLR3G*	*PFTK1*
		SOX2	*CYR61*	*MGEA5*	*SERPINA1*
		SSB	*ILF2*	*BLCAP*	*PIN4*
		TDGF1	*ILF3*	*GADD45G*	*PITX2*
		TEAD4	*ING1*	*MAGED2*	*EXOSC9*
		TERF1	*INPP4A*	*STMN2*	*POU2F1*

TFAM	*ITGB1*	*IL1RAPL1*	*POU5F1*
TIA1	*JARID2*	*HHLA3*	*PPM1B*
TMPO	*JUN*	*HYPE*	*PPP1R2*
TNNT1	*JUP*	*NUDT5*	*PPP1R10*
UGP2	*KCNN2*	*FZD10*	*PPP2R1A*
UNG	*KDR*	*RNF24*	*PPP2R1B*
VSNL1	*KPNA3*	*DUSP12*	*PPP2R3A*
ZIC2	*TNPO1*	*ZHX2*	*PPP2R5C*
ZIC3	*KRT18*	*DKK1*	*PRCC*
ZNF195	*LAMA4*	*SLC4A1AP*	*PRKAR1A*
LRP8	*LOH11CR2A*	*WDFY3*	*PRNP*
FZD5	*LRP2*	*PHF8*	*PRPSAP1*
DEK	*LRP3*	*ANKRD15*	*PRSS8*
FXR1	*NBR1*	*SULF1*	*PSEN2*
USP9X	*SMAD3*	*DNAJC9*	*PSMB1*
FZD7	*MAN2C1*	*FBXW11*	*PSMC2*
UTF1	*MCC*	*OBSL1*	*PTN*
IFITM1	*MDH1*	*SMG5*	*PTPN2*
TMEFF1	*MEF2A*	*PIP5K1C*	*PTPN3*
RUVBL1	*MICA*	*FRAT2*	*RAB5A*
PPAP2A	*MICB*	*COMMD3*	*RAD23A*
USO1	*MLH1*	*SLC44A1*	*RASA1*
ADAM23	*MAP3K11*	*ICMT*	*RASGRF2*
TRIM24	*MMP2*	*CHST5*	*REST*
HESX1	*MMP9*	*MKRN1*	*RFX1*
PROM1	*MOBP*	*SSBP2*	*RGS10*
FUBP1	*MOV10*	*ZKSCAN5*	*RPL7*

DDX18	*MRE11A*	*CBY1*	*RPL9*
MAP7	*MSH2*	*RAD54B*	*RPL15*
CLDN6	*MTM1*	*BAMBI*	*RPL30*
SYNGR3	*NAP1L2*	*TXN2*	*RPL36A*
BUB3	*NDUFB3*	*ABTB2*	*RPLP1*
DDX21	*NDUFS2*	*SFRS18*	*RPS3A*
DCLK1	*NKTR*	*PRPF31*	*RPS18*
AURKB	*NMT1*	*KIAA1279*	*RPS26*
NOLC1	*NODAL*	*PHGDH*	*RPS29*
PTTG1	*NP*	*ANKRD1*	*RTN2*
MED14	*NPAS2*	*SNX5*	*SORT1*
CER1	*ROR1*	*TJP3*	*VPS52*
HOMER1	*NVL*	*TNRC6A*	*SALL1*
C1orf38	*OAZ2*	*KCNMB4*	*SAT1*
GDF3	*ORC1L*	*MRPL13*	*SC5DL*
NFE2L3	*OXA1L*	*ATAD2*	*SCNN1A*
DLG7	*P4HA1*	*TFPT*	*CXCL5*
G3BP2	*PDCL*	*TRA2A*	*SDHD*
RABGAP1L	*PBX1*	*MYEF2*	*SET*
SRA1	*PCBP1*	*KLHL5*	*SFPQ*
CHAF1A	*PCNA*	*HN1*	*SFRP1*
DNAJB6	*PCTK1*	*DBR1*	*SFRP2*
AP1M2	*PCTK2*	*CXorf26*	*SFRS4*
G3BP1	*PDHB*	*PIPOX*	*SFRS7*
GPR64	*ENPP2*	*UBR5*	*SGTA*
CEBPZ	*PEX1*	*ATP6V1D*	*SH3GL3*
AASS	*PFTK1*	*UFM1*	*ST3GAL2*

PRMT3	*SERPINA1*	*ANKHD1*	*SKIL*
ZNF267	*PIK3R2*	*WDR70*	*SLC3A2*
TRIM22	*PITX2*	*RIF1*	*HLTF*
NPM3	*EXOSC9*	*RIC8B*	*SNAPC1*
TUBB2C	*PODXL*	*C12orf35*	*SNAPC3*
LYPLA1	*POU2F1*	*DPPA4*	*SNRPA*
OLFM1	*POU4F1*	*LSG1*	*SNRPD3*
MAD2L2	*POU5F1*	*TBC1D22B*	*SNRPN*
NOL5A	*PPP1R2*	*NPLOC4*	*SNX1*
PAICS	*PPP2R1A*	*RBM22*	*SOX2*
PAICS	*PPP2R1B*	*CCDC94*	*SP2*
POLR3G	*PPP2R3A*	*FLJ10769*	*SSR4*
RAD51AP1	*PPP2R5C*	*TMEM30A*	*STAT3*
LEFTY1	*PRCP*	*H2AFJ*	*STC1*
IGF2BP3	*PKIB*	*PRR11*	*STCH*
IGF2BP2	*MAPK8*	*JMJD1A*	*STXBP2*
MTHFD2	*PRNP*	*KLHL4*	*TAF12*
NMU	*PRSS8*	*PARD3*	*TAL1*
KIF2C	*PSEN2*	*SMARCAD1*	*TALDO1*
PIM2	*PSMA1*	*OLFML3*	*TARBP2*
NUDT21	*PSMB1*	*KIF15*	*TBP*
LECT1	*PSMB4*	*KIAA1143*	*TCF7L2*
DIDO1	*PSMB5*	*ARID1B*	*TCF20*
MYST2	*PSMC2*	*LRRN1*	*PPP1R11*
HRASLS3	*PSMD9*	*TMEM16H*	*TDGF1*
PSIP1	*PTN*	*TGIF2*	*LEFTY2*
WDHD1	*PTPN1*	*PRDM14*	*THBS2*

CHEK2	*PTPN2*	*LHPP*	*THOP1*
GPR176	*PXMP3*	*SOX17*	*TIA1*
OIP5	*QARS*	*ZDHHC6*	*TIAL1*
RRAS2	*RAB5A*	*NUCKS1*	*TIMP4*
MTF2	*RAB5B*	*DDX31*	*TLE1*
SEPHS1	*RAD23A*	*ARMCX5*	*TLE2*
GARNL4	*RANGAP1*	*ACD*	*TLE3*
TTLL12	*RAP1A*	*GNPTAB*	*TNFAIP2*
PASK	*RARB*	*LRFN3*	*TOP2A*
MDN1	*RASA1*	*C13orf7*	*TPM3*
COBL	*RASGRF2*	*EFTUD1*	*HSP90B1*
BOP1	*RBBP4*	*PRKRIP1*	*TUBG1*
NCAPH	*RBM4*	*TBL1XR1*	*TXNRD1*
FRAT2	*RBP1*	*C15orf29*	*UBC*
SIRT1	*REST*	*NANOG*	*UBE2D3*
CBX5	*RFX1*	*CNTNAP3*	*UBP1*
TNPO3	*RGS10*	*DHDDS*	*UFD1L*
PRKD3	*RNF2*	*PHF17*	*UGT8*
KIF4A	*RPL17*	*GRHL2*	*VASP*
RAD54B	*RPL21*	*URM1*	*VIM*
NOL11	*RPL32*	*C14orf156*	*ZIC1*
SFRS18	*RPLP1*	*TCF7L1*	*ZIC2*
LRIG1	*RPS3A*	*USP44*	*ZIC3*
CNTNAP2	*RPS13*	*C14orf153*	*ZNF140*
AUTS2	*RPS18*	*GTPBP3*	*ZNF217*
SERBP1	*RPS26*	*C14orf151*	*MAP3K12*
PITPNC1	*RPS27A*	*LINGO1*	*USP7*

GNL3	*RYR3*	*PRPF38A*	*FZD3*
FOXD3	*S100A11*	*MST150*	*MYST3*
ITGB1BP3	*VPS52*	*ATPBD4*	*MLLT10*
C6orf66	*SALL2*	*CABLES1*	*ANP32A*
CYP2S1	*SALL1*	*WDR20*	*SLC7A5*
PYCR2	*SC5DL*	*SFXN1*	*TAF15*
RRP15	*SCNN1A*	*PRKCDBP*	*MKKS*
GMNN	*SDC4*	*NAT12*	*CDC7*
GAL	*SEPP1*	*TMEM170*	*CDC45L*
FAM108B1	*SET*	*OSR1*	*EOMES*
PIPOX	*SFRP1*	*AASDH*	*FZD1*
ZNF589	*SFRP2*	*WDR36*	*FZD7*
RNF138	*SFRS4*	*C1orf211*	*HIST2H2AA3*
HSPC111	*SGK*	*COMMD7*	*HIST2H2BE*
LARP7	*SCG5*	*IRX2*	*HIST1H4C*
ESF1	*ST3GAL2*	*RDH10*	*TTF2*
AZIN1	*SILV*	*C9orf97*	*TEAD2*
LSR	*SKIL*	*SPRED1*	*OGT*
GINS2	*SLC1A1*	*SGMS1*	*CDC42BPA*
GPRC5B	*HLTF*	*AMIGO2*	*PIK3R3*
CECR1	*SNRP70*	*FAM33A*	*PARG*
BRWD1	*SNRPA*	*IER5L*	*AP3B1*
C21orf45	*SNRPE*	*MED11*	*CDC14B*
FAM64A	*SNRPN*		*PPAP2A*
PUS7	*SNX1*		*USO1*
EPB41L4B	*SON*		*EIF3F*
L1TD1	*SOX2*		*B3GALT4*

ERCC6L	*SP2*	*GBF1*
RBM35A	*SPARC*	*RIPK1*
NCAPG2	*STAT3*	*TRIM24*
ZNF770	*STC1*	*SAP30*
PAK1IP1	*AURKA*	*HESX1*
C12orf48	*STRN*	*TSC22D1*
FANCL	*STXBP2*	*PER2*
DPPA4	*SUPT4H1*	*SPAG9*
C14orf115	*TAF4B*	*SDCCAG1*
NUDT15	*TAF12*	*TMSB10*
BXDC2	*TAL1*	*BUB3*
C14orf106	*TALDO1*	*DDX21*
LGR4	*TARBP2*	*LARGE*
MCM10	*TBCC*	*LRAT*
PRPF40A	*TBL1X*	*PNMA1*
TMEM48	*TBP*	*MSC*
C12orf11	*TCF7L2*	*DHRS3*
CCAR1	*TCF20*	*B4GALT6*
WDR12	*TDGF1*	*CNOT8*
RCC2	*TERF1*	*CER1*
KLHL7	*LEFTY2*	*CDYL*
CHST7	*THBS2*	*MED17*
EXOSC5	*TIA1*	*GGPS1*
NUP107	*TIAL1*	*ATP6V1G1*
SALL4	*TIAM1*	*RBM39*
SLC39A10	*TIMP4*	*MPHOSPH1*
MRS2L	*TLE2*	*NFE2L3*

SPC25	*TLE3*	*RNF14*
NLN	*TSPAN6*	*FEZ1*
NLGN4X	*TOP2A*	*MORF4L2*
MTA3	*TSSC1*	*MDC1*
ZNF398	*TTF1*	*ZNF516*
SEMA6A	*TUBG1*	*KIAA0391*
LRRN1	*TXNRD1*	*PUM1*
CACHD1	*UBC*	*TMEM63A*
FAM60A	*UBE2D3*	*ZNF646*
PRDM14	*SUMO1*	*HDAC9*
NOC3L	*UBP1*	*KNTC1*
ISG20L1	*UFD1L*	*FAM115A*
SLC13A3	*VIM*	*KIAA0247*
CAPRIN2	*VLDLR*	*BCLAF1*
DBNDD1	*VRK2*	*DHX38*
CAMKV	*WARS*	*MTSS1*
NUP37	*WEE1*	*SPCS2*
ELOVL6	*ZIC1*	*ARHGAP11A*
DCC1	*ZIC2*	*ZEB2*
GNPTAB	*ZIC3*	*HEPH*
C1orf108	*CNBP*	*EPM2AIP1*
NARG2	*ZNF174*	*SMG7*
LIN28	*ZNF185*	*MED12*
NANOG	*ZNF202*	*NAALAD2*
PHF17	*ZNF217*	*ACOT8*
NARG1	*ZNF226*	*ABCF2*
MYO19	*ZNF228*	*DPP3*

PUS1	*MAP3K12*	*HUWE1*
TMEM177	*USP7*	*GPC6*
SLC38A1	*BAT3*	*ACTR1A*
TXNDC1	*MYST3*	*G3BP1*
WBSCR16	*MLLT10*	*SFRS14*
CDT1	*FXR1*	*DDX39*
SPRY4	*ANP32A*	*MRPS31*
TCF7L1	*SLC7A5*	*TRIM22*
BCL2L12	*MKKS*	*HMG20A*
USP44	*PICALM*	*TIMM23*
GINS4	*AXIN2*	*VAT1*
HPS3	*CDC7*	*DDX17*
RBM13	*CDC45L*	*NEBL*
SLC7A3	*EOMES*	*ANP32B*
ZSCAN10	*FZD7*	*ARFGEF1*
ANGEL2	*FZD8*	*POLR3G*
KIFC2	*HIST2H2AA3*	*TRIM16*
LOC91431	*HIST1H4C*	*PDPN*
C20orf72	*TTF2*	*LEFTY1*
DMKN	*PPFIBP1*	*EXOC5*
EGLN3	*PARG*	*IGF2BP3*
CDCA5	*AP3B1*	*GNA13*
MAL2	*CDC14B*	*MGEA5*
FAM46B	*KHSRP*	*RBBP9*
SCGB3A2	*KLF7*	*SDCCAG8*
GYLTL1B	*PPAP2A*	*C5orf4*
LOC157627	*EIF3D*	*NMU*

C8orf42	*EIF3F*	*GADD45G*
C11orf82	*PEA15*	*MAGED2*
ARL5B	*B3GALT4*	*MORF4L1*
TUBB2B	*GBF1*	*MSL3L1*
CKMT1A	*RIPK1*	*CLP1*
	CDS2	*MAPRE2*
	MTMR1	*KIF2C*
	MPDZ	*RBPMS*
	TRIM24	*UBE2C*
	DPM1	*DIDO1*
	HESX1	*HNRPUL1*
	ALKBH1	*PWP1*
	TSC22D1	*HYPE*
	CDC123	*NUDT4*
	CDC16	*FZD10*
	DDX18	*RNF24*
	AP1S2	*DUSP12*
	SPAG9	*CBX3*
	NFS1	*OIP5*
	PAPSS2	*MTF2*
	CLDN6	*LPHN1*
	PKMYT1	*DKK1*
	USP10	*MAST1*
	SEC22C	*ACIN1*
	SDCCAG1	*AZI1*
	TMSB10	*EXPH5*
	BUB3	*MRPS27*

LARGE	*PHF8*
VAPB	*NCDN*
NOLC1	*39331*
LRAT	*ZCCHC14*
PNMA1	*ANKRD15*
MSC	*KIAA0280*
DHRS3	*RRS1*
TRIP10	*SULF1*
TRIP4	*NUP160*
GTF3C4	*FBXW11*
B4GALT6	*NEDD4L*
CNOT8	*SASH1*
CER1	*DNAJC16*
DDX23	*OBSL1*
CDYL	*KIAA0368*
MED23	*PIP5K1C*
EIF2AK3	*FRAT2*
PIGL	*COMMD3*
EEF1E1	*SLC44A1*
TMEM59	*ICMT*
BAG5	*CBX5*
ATP6V1G1	*ETHE1*
SEC22B	*ZNF281*
GTPBP1	*ORC6L*
GDF3	*PPP1R15A*
PREPL	*SSBP3*
RBM39	*SLC7A11*

NFE2L3	*SGK3*
NCOR1	*GSPT2*
FEZ1	*C9orf5*
MORF4L2	*CBY1*
ZNF516	*RAB3GAP2*
KIAA0391	*RAD54B*
FAM131B	*LSM4*
TMEM63A	*BAMBI*
SART3	*ARIH1*
KNTC1	*ABTB2*
FAM115A	*USP49*
KIAA0101	*CLIC4*
BCLAF1	*FAM98A*
KIAA0652	*C20orf194*
DHX38	*SFRS18*
DLG7	*TBC1D10B*
SFI1	*PLEKHG3*
TSC22D2	*GGA1*
ARHGAP11A	*KIAA1279*
LCMT2	*TRPC4AP*
ZEB2	*KIF26A*
EPM2AIP1	*TIMM9*
SUPT7L	*TIMM8B*
NCAPD2	*HBP1*
JOSD1	*ZRF1*
USP3	*ANKRD1*
MED12	*FOXP1*

NAALAD2	*TJP3*
ACOT8	*EIF2C2*
ABCF2	*SLC39A1*
SNUPN	*LSM3*
GPC6	*TNRC6A*
ACTR1B	*PRPF19*
ACTR1A	*KCNMB4*
SFRS14	*MAT2B*
CEBPZ	*NKIRAS1*
CHST4	*MAPBPIP*
RBM7	*C11orf67*
DDX39	*MRPS18B*
PSMD14	*MCTS1*
SPRY1	*ATAD2*
KATNB1	*MRPL15*
TRIM22	*UBE2T*
HMG20A	*C16orf80*
CITED2	*SCG3*
CEPT1	*USP25*
YAP1	*UCRC*
TIMM23	*TRA2A*
RBM14	*NME7*
HMGN4	*AK3*
SEMA4F	*COPS7A*
SEMA3C	*C14orf122*
DDX17	*TXNDC12*
NEBL	*KLHL5*

ANP32B	*APH1A*
CCT7	*RDH11*
SORBS1	*HSD17B12*
AHSA1	*ING4*
USP16	*HN1*
POLR3G	*NUSAP1*
TRIM16	*CRIM1*
PDPN	*PIPOX*
GAS2L1	*MEX3C*
LEFTY1	*UBR5*
IGF2BP3	*NIP7*
KHDRBS1	*C11orf73*
CUGBP2	*UFM1*
GNA13	*LARP7*
CCT6B	*PCF11*
MGEA5	*NAG*
NFAT5	*MRPS23*
RAI1	*SUFU*
SEC24A	*CYB5R2*
UTP14A	*UIMC1*
C5orf4	*WBP11*
NMU	*ARID4B*
BLCAP	*CRKRS*
TCERG1	*RSF1*
MORF4L1	*RNUXA*
MSL3L1	*FXYD5*
SERINC3	*RAB4B*

TMED10	*POLE3*
KDELR3	*ERRFI1*
RABL4	*GDAP1*
RBPMS	*SLC38A2*
PIM2	*CCDC93*
ABHD2	*FBXL19*
LECT1	*FAM63B*
DIDO1	*RRN3*
HNRPUL1	*TRIT1*
KRR1	*TMEM103*
PWP1	*ANKHD1*
IL1RAPL1	*FLJ20309*
PKIG	*WHSC1L1*
NUDT5	*TMEM160*
WDHD1	*TIPIN*
WDR6	*ZNF770*
FZD10	*ZSCAN2*
DDX20	*PIH1D1*
RNF24	*PPP2R3C*
DUSP12	*C14orf119*
COPE	*RNF31*
XAB1	*C6orf166*
CBX3	*RBM23*
OIP5	*DARS2*
DNAJC8	*RIF1*
ELL2	*RIC8B*
DKK1	*C12orf35*

SLC4A1AP	*DPPA4*
TPX2	*C14orf115*
NT5C2	*UBE2W*
FBXL11	*LSG1*
WDTC1	*ZNF331*
KIAA0241	*FEM1A*
EXPH5	*STAP2*
SPG20	*OSGEP*
PHF8	*HIF1AN*
39331	*IWS1*
ANKRD15	*RBM22*
RRS1	*CCDC94*
SULF1	*POLR3E*
DNAJC9	*VPS35*
KLHL18	*FLJ10769*
FBXW11	*C14orf108*
EHBP1	*RCOR3*
SIN3B	*ZNF701*
NEDD4L	*H2AFJ*
SASH1	*PRR11*
DNAJC16	*FOXJ2*
OBSL1	*JMJD1A*
SMG5	*EAPP*
KIAA0368	*CISD1*
FRAT2	*MYNN*
COTL1	*KIAA1166*
COMMD3	*UBQLN4*

SLC44A1	*BDH2*
SF3B1	*SPIRE1*
ICMT	*EXOSC5*
CBX5	*SMARCAD1*
ISCU	*OLFML3*
LEPROTL1	*XAB2*
HEY2	*TNFSF5IP1*
KCTD2	*KIF15*
R3HDM1	*AVEN*
ZNF281	*RAB25*
RBM9	*MRPL47*
CDC42EP4	*KIAA0495*
ORC6L	*ZNF286A*
MKRN1	*KIAA1143*
SSBP2	*SFRS15*
SSBP3	*ODF2L*
SLC7A11	*ARID1B*
LSM5	*NUFIP2*
SGK3	*SEMA6A*
RAB38	*ARRDC3*
GSPT2	*RANBP10*
C9orf5	*LRRN1*
RAB3GAP2	*CACHD1*
RAD54B	*TMEM16H*
BAMBI	*SCAF1*
ARIH1	*FAM60A*
METTL7A	*SENP2*

WDSOF1	*TGIF2*
POLR1A	*SAV1*
ZNF473	*NSUN3*
RP11-529I10.4	*ZNF335*
ZNF521	*PRDM14*
CLIC4	*LHPP*
C20orf194	*RAB17*
SFRS18	*NOC3L*
TMEM87A	*MOSPD3*
TBC1D10B	*NUCKS1*
GORASP2	*MRPS11*
PLEKHG3	*NOL6*
PPP1R16B	*RSRC2*
GGA1	*C1orf163*
PRPF31	*PLEKHA3*
TRPC4AP	*RASL11B*
KIF26A	*TMEM108*
PHGDH	*C19orf43*
BSCL2	*SCNM1*
HBP1	*C19orf58*
ZRF1	*TMEM109*
ANKRD1	*DCC1*
STK36	*WDR77*
SALL3	*PHF23*
SERP1	*GNPTAB*
LSM3	*LRFN3*
APEX2	*C13orf7*

TNRC6A	*C14orf138*
GOLIM4	*HMBOX1*
UBE2S	*C1orf54*
PRPF19	*C1orf108*
POLL	*PRKRIP1*
MAT2B	*TBL1XR1*
BZW2	*TBC1D17*
MCTS1	*ZNF668*
ATAD2	*C15orf29*
C16orf72	*VASH2*
UBE2T	*DCAKD*
HSPC171	*NANOG*
TBK1	*KIAA0319L*
CYP2S1	*DHDDS*
UCRC	*PHF17*
TFPT	*GRHL2*
NME7	*RMI1*
LMCD1	*C14orf159*
SLC40A1	*PIF1*
EHD4	*MUS81*
MYEF2	*FBXO11*
AK3	*WDR23*
F11R	*C1orf21*
C14orf122	*URM1*
C1orf121	*FIP1L1*
NDUFA13	*DIAPH3*
KLHL5	*MED25*

IFT52	*SLC7A5P1*
C8orf70	*TCF7L1*
APH1A	*SF3B5*
HSD17B12	*ABHD11*
ING4	*C22orf13*
HN1	*USP44*
SS18L2	*ARID5B*
NUSAP1	*FAM96A*
VRK3	*POLDIP3*
C3orf19	*LSMD1*
MRPL37	*AKT1S1*
CXorf26	*PPAPDC1B*
MRPL27	*MRPL43*
PIPOX	*GTPBP3*
ARMCX1	*HDGF2*
TNFRSF12A	*LINGO1*
UBR5	*CCDC123*
NIP7	*ZFYVE19*
DDX41	*PRPF38A*
HSPC111	*MPND*
LARS	*JUB*
UFM1	*MST150*
MIR16	*ZCCHC3*
LARP7	*ZCRB1*
ASB1	*ATPBD4*
TMEM66	*CCDC45*
MPP6	*TMEM55B*

SUFU	*BTF3L4*
UIMC1	*CABLES1*
WBP11	*ZNF300*
NUP54	*HIST3H2A*
FXYD5	*PERLD1*
MYO3A	*ARMC6*
PPIL3	*SFXN1*
ZFAND6	*C21orf66*
CCDC93	*TSGA14*
FAM35A	*EGLN3*
DDX49	*PRKCDBP*
FAM63B	*OSBPL1A*
RRN3	*TMEM123*
DYM	*WDFY2*
C10orf26	*LYPD1*
LRRC49	*NAT12*
ANKRD49	*TMEM170*
TMEM103	*HEXIM2*
WHSC1L1	*WDR81*
RPP25	*C18orf37*
ZNF434	*ZNF428*
TMEM160	*NDUFA11*
HCFC1R1	*AASDH*
ZNF770	*WDR36*
PIH1D1	*C20orf96*
PPP2R3C	*HECTD2*
PRPF39	*XRRA1*

PTCD3	*FAM76B*
RNF31	*ZNF664*
FANCL	*C12orf60*
C6orf166	*FBXL14*
THAP1	*FLJ40125*
DARS2	*CREB3L4*
RIF1	*C1orf211*
RIC8B	*COMMD7*
NADSYN1	*IRX2*
C12orf35	*AMOTL1*
P15RS	*RDH10*
DPPA4	*FBXO16*
EXDL2	*C8orf42*
C14orf115	*C9orf97*
UBE2W	*SPRED1*
RNF121	*FAM134C*
SLC39A9	*C1orf55*
ZNF331	*UBR1*
C2orf56	*TUBB*
ETNK1	*FLJ25801*
FEM1A	*FAM124A*
OTUB1	*C6orf130*
C20orf42	*SGMS1*
OSGEP	*PGM2L1*
IWS1	*GLT8D3*
RBM22	*FAM100B*
CCDC94	*ZIK1*

VPS35
FLJ10769
ZNF701
H2AFJ
PRR11
FOXJ2
JMJD1A
CAND1
UBAP2
WWC3
C3orf10
CISD1
PSENEN
APOM
LIN37
GNG12
KLHL4
NDNL2
KIAA1217
EIF4ENIF1
C21orf59
GRIPAP1
C15orf24
UBQLN4
EXOSC5
SMARCAD1
GPR108
C19orf54
C1orf174
ZNF677
FAM33A
RAB15
IMAA
IER5L
PCNXL3

FEM1C
OLFML3
XAB2
OTUD7B
FAM20C
KIF15
ANKMY2
RAB25
INTS12
PHTF2
ZNF286A
KIAA1143
SFRS15
ODF2L
ARID1B
ZNF398
SLAIN2
RANBP10
LRRN1
CACHD1
TMEM16H
NOPE
GATAD1
SCAF1
EPS15L1
C6orf115
SENP2

EXOC4
SAV1
NIF3L1
C14orf133
NSUN3
PRDM14
PERP
LHPP
KIF9
TFB2M
RAB17
NOC3L
RNF25
DCLRE1C
TTC31
NUCKS1
C11orf1
39148
NOL6
TMEM135
RSRC2
PLEKHA3
RASL11B
TMEM108
SCNM1
GIYD2
C19orf58

KCTD15
SECISBP2
TMEM109
DCC1
WDR77
C19orf42
PHF23
GNPTAB
TMEM43
LRFN3
CARS2
C13orf7
C14orf138
HMBOX1
C1orf54
C1orf108
HSPBAP1
PARP8
TMEM149
TBL1XR1
PALB2
ISOC2
C15orf29
FBXO31
ALG9
GSTCD
MOBKL2B

ASAM
METTL8
DCAKD
C9orf82
NANOG
CNTNAP3
DHDDS
PHF17
GRHL2
DNAJB14
NIP30
UXS1
FLJ22795
MUS81
C2orf44
WDR23
CYB5B
ECOP
C1orf21
TXNDC5
URM1
MAP1LC3B
MED25
C14orf156
SLC7A5P1
TCF7L1
SF3B5

ABHD11
EIF2A
USP44
TOMM40L
ARID5B
ASCC2
FAM96A
TRAF7
DCUN1D5
ALKBH7
CHCHD5
LSMD1
C14orf153
SPIRE2
DCTN5
GTPBP3
FKSG24
C14orf151
SFT2D3
ADO
ZSCAN10
LINGO1
DIRC2
ATG4C
PRPF38A
JUB
TMEM60

MST150
ZCCHC3
ZCRB1
RSPRY1
ATPBD4
ZNF551
CCDC45
TMEM55B
TCEAL8
C19orf6
C22orf32
CABLES1
WDR20
COG7
ZNF300
HIST3H2A
PERLD1
ARMC6
ATPIF1
EGLN3
PRKCDBP
SAT2
C6orf117
CCDC104
FAM54A
DTX2
STK11IP

OSBPL1A
TMEM123
TLCD1
LYPD1
SCGB3A2
SLC36A4
CYP2R1
LRIG3
NAT12
C16orf63
IQCK
FAM100A
TMEM170
HEXIM2
WDR81
C18orf37
TYW3
ZNF684
C1orf83
TMEM77
MBOAT2
OSR1
ZFP42
AASDH
GRPEL2
WDR36
FAM92A1

UNC5D
C20orf96
C20orf52
HECTD2
ZNF664
C12orf60
FBXL14
C1orf211
SLC30A7
C1orf213
COMMD7
CCDC12
C9orf19
MARVELD2
IRX2
AMOTL1
C8orf42
SPRED1
ADAL
FAM134C
ASXL1
HIGD2A
THAP8
FLJ25801
C11orf82
FAM124A
ZNRF2

SGMS1
BCL9L
GLT8D3
FAM100B
ZIK1
C19orf54
WDR62
RABL3
ATP11C
AMIGO2
FAM33A
UNQ501
RAB15
IMAA
FLJ45455
IER5L
PCNXL3
FAM128A
FAM72B

Supplementary Table 5. Immunohistochemistry staining results for 10 patiets

	MRTFA	***SRF***	***IGFBP5***
Patient24	O	O	O
Patient20	O	O	O
Patient11	O	O	O
Patient08	O	O	O
Patient05	O	O	O
Patient12	O	O	O
Patient06		O	O
Patient15	O	O	O
Patient04	O	O	O
Patient03	O	O	O

MRTFA: Myocardin related transcription factor A

SRF: Serum response factor

IGFBP5: Insulin like growth factor binding protein 5

Supplementary Table 6. Hotspot mutation lists with oncogenes

ID	Mutation	Hugo_Symbol	Chromosome	Start_Position	End_Position	Reference_Allele	Tumor_Seq_Allele2	HGVSp_Short	Existing_variation	Type	Lauren	Cell type*
P01A-ACCGTAATCCTTTCTC-1	CCND1:11:69466021	*CCND1*	11	69466021	69466021	C	T	p.P287S	COSM4855094,COSM4855095,COSM931396	Adjacent non-cancer	Intestinal	Enteroendocrine
P01A-TGGCCAGCACCTCGGA-1	BAP1:3:52443593	*BAP1*	3	52443593	52443595	GTA	-	p.Y33del		Adjacent non-cancer	Intestinal	EC
P01B-ATTTCTGTCAGTTTGG-1	RAC1:7:6441974	*RAC1*	7	6441974	6441974	C	T	p.A178V	COSM1154840,COSM389868	Cancer	Intestinal	Tumor

P01B-CCTTACGGTACCATCA-1	KRAS:12:25380282	*KRAS*	12	25380282	25380282	G	T	p.A59E	COSM1135365,COSM1318029,COSM28518,COSM547	Cancer	Intestinal	Tumor
P01B-CCTTACGGTACCATCA-1	KRAS:12:25380278	*KRAS*	12	25380278	25380278	A	C	p.G60=	COSM1159613,COSM1168050,COSM253757	Cancer	Intestinal	Tumor
P01B-CGGACTGTCGACAGCC-1	SMARCB1:22:24176330	*SMARCB1*	22	24176330	24176330	G	A	p.R374Q	rs1057517825,COSM1266245,COSM998	Cancer	Intestinal	Tumor
P01B-TACTTACAGCCACCTG-1	KRAS:12:25378561	*KRAS*	12	25378561	25378561	G	A	p.A146V	rs1057519725,COSM1360827,COSM19900,COSM5752083,COSM5752084	Cancer	Intestinal	Tumor
P01B-TTCT	CCND1:1	*CCN*	11	6946	694	C	G	p.P2	COSM4855094,COSM4855095,COSM931396	Cancer	Int	PMC

CCTAGTCGAGTG-1	1:69466021	*D1*		6021	66021			87A			estinal	
P02A-AAAGTAGTCAGGATCT-1	CCND1:11:69466022	*CCND1*	11	69466022	69466022	C	T	p.P287L	COSM2043470,COSM226265,COSM931397	Adjacent non-cancer	Diffuse	MSC
P02A-ACACTGACATAAAGGT-1	RAC1:7:6441974	*RAC1*	7	6441974	6441974	C	T	p.A178V	COSM1154840,COSM389868	Adjacent non-cancer	Diffuse	PC
P02A-ACAGCCGGTGTGGCTC-1	CCND1:11:69466021	*CCND1*	11	69466021	69466021	C	A	p.P287T	COSM4855094,COSM4855095,COSM931396	Adjacent non-cancer	Diffuse	Tumor
P02A-ACCCACTCAAGAAAGG-1	SF3B1:2:198266830	*SF3B1*	2	198266830	198266830	A	G	p.V701A	COSM4569855,COSM4745929	Adjacent non-cancer	Diffuse	GMC

P02A-ACCTTTACATCACCCT-1	RAC1:7:6441974	*RAC1*	7	6441974	6441974	C		T	p.A178V	COSM1154840,COSM389868	Adjacent non-cancer	Diffuse	GMC
P02A-ACTGCTCGTCGGCACT-1	RAC1:7:6431628	*RAC1*	7	6431628	6431628	C		A	p.Q61K		Adjacent non-cancer	Diffuse	PC
P02A-AGAATAGCAAAGTGCG-1	RAC1:7:6441974	*RAC1*	7	6441974	6441974	C		T	p.A178V	COSM1154840,COSM389868	Adjacent non-cancer	Diffuse	Tumor
P02A-AGGCCGTGTCTCTTAT-1	RAC1:7:6441974	*RAC1*	7	6441974	6441974	C		T	p.A178V	COSM1154840,COSM389868	Adjacent non-cancer	Diffuse	PMC
P02A-CACAGTAGTCCG	RAC1:7:6441974	*RAC1*	7	6441974	6441974	C		T	p.A178V	COSM1154840,COSM389868	Adjacent non-cancer	Diffuse	Tumor

TTAA-1												
P02A-CACAGTATCGCAAGCC-1	RAC1:7:6441974	*RAC1*	7	6441974	6441974	C	T	p.A178V	COSM1154840,COSM389868	Adjacent non-cancer	Diffuse	PMC
P02A-CCATGTCCACCAGGCT-1	KRAS:12:25378648	*KRAS*	12	25378648	25378648	T	C	p.K117R	COSM4696721,COSM4696722	Adjacent non-cancer	Diffuse	Tumor
P02A-GAAATGACACTCGACG-1	CCND1:11:69466022	*CCND1*	11	69466022	69466022	C	T	p.P287L	COSM2043470,COSM226265,COSM931397	Adjacent non-cancer	Diffuse	GMC
P02A-GAACCTAGTCCGAACC-1	FBXW7:4:153250906	*FBXW7*	4	153250906	153250906	G	A	p.T385I	COSM1309812,COSM1309813,COSM1309814,COSM1309815,COSM1309816	Adjacent non-cancer	Diffuse	PMC
P02A-GCA	CCND1:1	*CCN*	11	6946	694	C	T	p.P2	COSM4855094,COSM4855095,COSM931396	Adjacent	Diff	PC

GCCACAGCCTTTC-1	1:69466021	*D1*		6021	66021				87S		non-cancer	use	
P02A-GCGCCAAAGCATGGCA-1	CCND1:11:69466022	*CCND1*	11	69466022	69466022	C		T	p.P287L	COSM2043470,COSM226265,COSM931397	Adjacent non-cancer	Diffuse	GMC
P02A-TAGCCGGTCATAGCAC-1	CCND1:11:69466021	*CCND1*	11	69466021	69466021	C		A	p.P287T	COSM4855094,COSM4855095,COSM931396	Adjacent non-cancer	Diffuse	PMC
P02A-TGCGCAGCAGCATACT-1	KRAS:12:25378648	*KRAS*	12	25378648	25378648	T		C	p.K117R	COSM4696721,COSM4696722	Adjacent non-cancer	Diffuse	MSC
P02B-ACGTCAATCTGTTTGT-1	CREBBP:16:3786707	*CREBBP*	16	3786707	3786707	A		T	p.W1502R	COSM5363732	Cancer	Diffuse	MSC

P02B-ACTGCTCTCTATGTGG-1	RAC1:7:6441974	*RAC1*	7	6441974	6441974	C	T	p.A178V	COSM1154840,COSM389868	Cancer	Diffuse	PC
P02B-AGAGCTTAGGGATACC-1	CCND1:11:69466018	*CCND1*	11	69466018	69466018	A	G	p.T286A		Cancer	Diffuse	PMC
P02B-AGGCCACGTTACGTCA-1	CCND1:11:69466022	*CCND1*	11	69466022	69466022	C	G	p.P287R	COSM2043470,COSM226265,COSM931397	Cancer	Diffuse	GMC
P02B-CAACTAGAGTGGGCTA-1	SDHAF2:11:61197647	*SDHAF2*	11	61197647	61197647	C	T	p.S10L	COSM4836035	Cancer	Diffuse	Tumor
P02B-CACAGGCAGAAT	RAC1:7:6441974	*RAC1*	7	6441974	6441974	C	T	p.A178V	COSM1154840,COSM389868	Cancer	Diffuse	MSC

TGTG-1												
P02B-CACAGGCCATAGTAAG-1	CCND1:11:69466022	*CCND1*	11	69466022	69466022	C	G	p.P287R	COSM2043470,COSM226265,COSM931397	Cancer	Diffuse	PC
P02B-CACATTTAGACGACGT-1	SMAD4:18:48604706	*SMAD4*	18	48604706	48604706	G	T	p.G510*	COSM6056828	Cancer	Diffuse	Tumor
P02B-CAGAATCTCATAAAGG-1	CCND1:11:69466022	*CCND1*	11	69466022	69466022	C	T	p.P287L	COSM2043470,COSM226265,COSM931397	Cancer	Diffuse	PMC
P02B-CAGTAACCAGTAAGCG-1	RAC1:7:6441974	*RAC1*	7	6441974	6441974	C	T	p.A178V	COSM1154840,COSM389868	Cancer	Diffuse	PMC
P02B-CATCAA	SDHA:5:2	*SDHA*	5	256470	2564	G	A	p.V644M	rs3211483,COSM6170729	Cancer	Difu	MSC

GGTCCAACTA-1	56470				70						se	
P02B-CCTCTGACAGCTGCTG-1	ETV6:12:11803095	*ETV6*	12	11803095	11803095	G	A	p.X11_splice	COSM5948338	Cancer	Diffuse	GMC
P02B-CGGACACCACTTGGAT-1	CCND1:11:69466022	*CCND1*	11	69466022	69466022	C	T	p.P287L	COSM2043470,COSM226265,COSM931397	Cancer	Diffuse	PC
P02B-CGGTTAAGTGTGAAAT-1	RAC1:7:6441974	*RAC1*	7	6441974	6441974	C	T	p.A178V	COSM1154840,COSM389868	Cancer	Diffuse	PC
P02B-CGTGTCTAGGATGGTC-1	RAC1:7:6439807	*RAC1*	7	6439807	6439807	T	G	p.N111K	COSM3640063,COSM3640064,COSM5038555,COSM5038556	Cancer	Diffuse	PMC

P02B-CTAATGGTCACAACGT-1	RAC1:7:6431629	*RAC1*	7	6431629	6431629	A	G	p.Q61R	COSM1131540	Cancer	Diffuse	PC
P02B-CTAATGGTCACAACGT-1	CCND1:11:69466018	*CCND1*	11	69466018	69466018	A	G	p.T286A		Cancer	Diffuse	PC
P02B-CTCATTAGTATTCTCT-1	CCND1:11:69466021	*CCND1*	11	69466021	69466021	C	G	p.P287A	COSM4855094,COSM4855095,COSM931396	Cancer	Diffuse	Chief
P02B-CTGATCCTCCGTTGTC-1	SDHA:5:256470	*SDHA*	5	256470	256470	G	T	p.V644L	COSM6170729	Cancer	Diffuse	Tumor
P02B-CTGGTCTAGCGA	SMARCB1:22:2417	*SMARCB1*	22	24176339	241763	G	T	p.R377L	CM122478,COSM1578803,COSM27977,COSM4596765,COSM4596766,COSM989	Cancer	Diffuse	GMC

TCCC-1	6339				39							
P02B-GACGGCTTCCGTCAAA-1	RAC1:7:6441974	*RAC1*	7	6441974	6441974	C	T	p.A178V	COSM1154840,COSM389868	Cancer	Diffuse	PC
P02B-GCAGCCAAGGAATTAC-1	CCND1:11:69466021	*CCND1*	11	69466021	69466021	C	G	p.P287A	COSM4855094,COSM4855095,COSM931396	Cancer	Diffuse	GMC
P02B-GGGAGATCAATAGCAA-1	RAC1:7:6441974	*RAC1*	7	6441974	6441974	C	T	p.A178V	COSM1154840,COSM389868	Cancer	Diffuse	PC
P02B-GGGCACTTCACAATGC-1	CCND1:11:69466022	*CCND1*	11	69466022	69466022	C	G	p.P287R	COSM2043470,COSM226265,COSM931397	Cancer	Diffuse	GMC
P02B-GGTGAA	EIF1AX:X:20	*EIF1*	X	20156719	2015	C	A	p.R13L	COSM1119080	Cancer	Difu	PC

GAGTACGTTC-1	156719	*AX*			6719						se	
P02B-GTATCTTGTAAGAGGA-1	RAC1:7:6441974	*RAC1*	7	6441974	6441974	C	T	p.A178V	COSM1154840,COSM389868	Cancer	Diffuse	MSC
P02B-GTGCATATCCTTGCCA-1	CCND1:11:69466022	*CCND1*	11	69466022	69466022	C	G	p.P287R	COSM2043470,COSM226265,COSM931397	Cancer	Diffuse	PMC
P02B-TAAGCGTGTTCAGCGC-1	RAC1:7:6441974	*RAC1*	7	6441974	6441974	C	T	p.A178V	COSM1154840,COSM389868	Cancer	Diffuse	PMC
P02B-TCACAAGCAGCTGGCT-1	CCND1:11:69466022	*CCND1*	11	69466022	69466022	C	G	p.P287R	COSM2043470,COSM226265,COSM931397	Cancer	Diffuse	PC

P02B-TCCACACGTCTAGTCA-1	RAC1:7:6441974	*RAC1*	7	6441974	6441974	C	T	p.A178V	COSM1154840,COSM389868	Cancer	Diffuse	MSC
P02B-TGAGGGAGTTCGTGAT-1	CCND1:11:69466022	*CCND1*	11	69466022	69466022	C	G	p.P287R	COSM2043470,COSM226265,COSM931397	Cancer	Diffuse	PC
P02B-TGTCCCATCTAACGGT-1	RAC1:7:6426892	*RAC1*	7	6426892	6426892	C	A	p.P29T	COSM1167878,COSM125734	Cancer	Diffuse	PC
P02B-TGTCCCATCTAACGGT-1	RAC1:7:6426892	*RAC1*	7	6426892	6426893	CC	TT	p.P29F		Cancer	Diffuse	PC
P02B-TTAGGACGTCTG	RAC1:7:6441974	*RAC1*	7	6441974	6441974	C	T	p.A178V	COSM1154840,COSM389868	Cancer	Diffuse	PC

GAGA-1												
P03A-CACCACTAGCCAGTAG-1	CCND1:11:69466021	*CCND1*	11	69466021	69466021	C	G	p.P287A	COSM4855094,COSM4855095,COSM931396	Adjacent non-cancer	Diffuse	MSC
P03A-CCTTCGATCCCTCAGT-1	CCND1:11:69466022	*CCND1*	11	69466022	69466022	C	G	p.P287R	COSM2043470,COSM226265,COSM931397	Adjacent non-cancer	Diffuse	GMC
P03A-CTGATCCTCTGATACG-1	KRAS:12:25380278	*KRAS*	12	25380278	25380278	A	T	p.G60=	rs397517037,COSM1159613,COSM1168050,COSM253757	Adjacent non-cancer	Diffuse	GMC
P03A-TCGCGAGCAAGAGTCG-1	CCND1:11:69466022	*CCND1*	11	69466022	69466022	C	G	p.P287R	COSM2043470,COSM226265,COSM931397	Adjacent non-cancer	Diffuse	PMC
P03B-ACCCAC	RAC1:7:6	*RAC1*	7	6441974	6441	C	T	p.A178V	COSM1154840,COSM389868	Cancer	Diffu	GMC

TTCCTCGCAT-1	441974				974						se	
P03B-ACGAGCCGTATAATGG-1	KRAS:12:25380277	*KRAS*	12	25380277	25380278	GA	TT	p.Q61K	COSM4387500,COSM87298	Cancer	Diffuse	MSC
P03B-ACGAGCCGTATAATGG-1	KRAS:12:25380277	*KRAS*	12	25380277	25380277	G	C	p.Q61E	rs121913238,COSM1159597,COSM549,COSM550	Cancer	Diffuse	MSC
P03B-AGCATACTCAGCAACT-1	RAC1:7:6441974	*RAC1*	7	6441974	6441974	C	T	p.A178V	COSM1154840,COSM389868	Cancer	Diffuse	PMC
P03B-CAAGTTGGTTAAAGTG-1	RAC1:7:6441974	*RAC1*	7	6441974	6441974	C	T	p.A178V	COSM1154840,COSM389868	Cancer	Diffuse	Tumor

P03B-CCGTGGACAGCTTCGG-1	RAC1:7:6441974	*RAC1*	7	6441974	6441974	C	T	p.A178V	COSM1154840,COSM389868	Cancer	Diffuse	Tumor
P03B-CCTTCCCAGTACCGGA-1	SMAD2:18:45368211	*SMAD2*	18	45368211	45368211	G	C	p.S464*	COSM268154,COSM268520,COSM4169157	Cancer	Diffuse	Tumor
P03B-CGATTGACACTGTCGG-1	ETV6:12:11803095	*ETV6*	12	11803095	11803095	G	A	p.X11_splice	COSM5948338	Cancer	Diffuse	MSC
P03B-CGGACACAGCTAGTCT-1	RAC1:7:6439806	*RAC1*	7	6439806	6439806	A	T	p.N111I	COSM1684687,COSM5624655,COSM5624656	Cancer	Diffuse	PC
P03B-CTCAGAACAGGG	RAC1:7:6441974	*RAC1*	7	6441974	6441974	C	T	p.A178V	COSM1154840,COSM389868	Cancer	Diffuse	Tumor

TTAG-1												
P03B-GACTAACTCGAACGGA-1	RAC1:7:6441974	*RAC1*	7	6441974	6441974	C	T	p.A178V	COSM1154840,COSM389868	Cancer	Diffuse	GMC
P03B-GCACATATCTGCTTGC-1	RAC1:7:6441974	*RAC1*	7	6441974	6441974	C	T	p.A178V	COSM1154840,COSM389868	Cancer	Diffuse	MSC
P03B-GCGAGAAGTCAATGTC-1	RAC1:7:6439807	*RAC1*	7	6439807	6439807	T	G	p.N111K	COSM3640063,COSM3640064,COSM5038555,COSM5038556	Cancer	Diffuse	Tumor
P03B-GGCTCGAAGAATCTCC-1	CCND1:11:69466021	*CCND1*	11	69466021	69466021	C	A	p.P287T	COSM4855094,COSM4855095,COSM931396	Cancer	Diffuse	PMC
P03B-GTAACT	CCND1:1:6	*CCN*	11	69466021	6946	C	G	p.P287A	COSM4855094,COSM4855095,COSM931396	Cancer	Difu	MSC

GCACAAGACG-1	9466021	*D1*			6021						se	
P03B-GTGCAGCTCGCTTAGA-1	CCND1:11:69466021	*CCND1*	11	69466021	69466021	C	G	p.P287A	COSM4855094,COSM4855095,COSM931396	Cancer	Diffuse	GMC
P03B-GTGGGTCCAGTCGTGC-1	RAC1:7:6441974	*RAC1*	7	6441974	6441974	C	T	p.A178V	COSM1154840,COSM389868	Cancer	Diffuse	PC
P03B-TCTCTAATCTTCCTTC-1	NFE2L2:2:178098810	*NFE2L2*	2	178098810	178098810	C	A	p.E79*	COSM120958,COSM132851,COSM1631472	Cancer	Diffuse	PC
P03B-TCTGAGATCTGTCAAG-1	SPOP:17:47696450	*SPOP*	17	47696450	47696450	A	C	p.F125V	COSM95273	Cancer	Diffuse	EC

P03B-TGCACCTTCTACTATC-1	CCND1:11:69466021	*CCND1*	11	69466021	69466021	C	T	p.P287S	COSM4855094,COSM4855095,COSM931396	Cancer	Diffuse	GMC
P03B-TGCGCAGTCTGTTGAG-1	CCND1:11:69466021	*CCND1*	11	69466021	69466021	C	A	p.P287T	COSM4855094,COSM4855095,COSM931396	Cancer	Diffuse	MSC
P03B-TGTTCCGAGATCCCAT-1	RAC1:7:6441974	*RAC1*	7	6441974	6441974	C	T	p.A178V	COSM1154840,COSM389868	Cancer	Diffuse	MSC
P03B-TTGCGTCGTCTCTTAT-1	PTPN11:12:112888199	*PTPN11*	12	112888199	112888199	C	G	p.A72G	rs121918454,CM013417,COSM13015,COSM13035,COSM5945277	Cancer	Diffuse	Tumor
P04A-CTGAAGTCACGT	CCND1:11:6946	*CCND1*	11	69466019	694660	C	T	p.T286I	COSM931395	Adjacent non-	Diffuse	PMC

GAGA-1	6019				19					cancer		
P04B-TCATTACGTGACGCCT-1	CCND1:11:69466022	*CCND1*	11	69466022	69466022	C	T	p.P287L	COSM2043470,COSM226265,COSM931397	Cancer	Diffuse	MSC
P05A-CTGTGCTCAGGCTCAC-1	RAC1:7:6441974	*RAC1*	7	6441974	6441974	C	T	p.A178V	COSM1154840,COSM389868	Adjacent non-cancer	Diffuse	EC
P05A-GGAAAGCTCATTCACT-1	FBXW7:4:153244092	*FBXW7*	4	153244092	153244092	G	A	p.R689W	COSM1154288,COSM206681,COSM206682,COSM206683,COSM27083,COSM5751359,COSM5751360,COSM5751361,COSM5751362,COSM5751363	Adjacent non-cancer	Diffuse	GMC
P05B-TAAACCGTCGGTTAAC-1	RAC1:7:6441974	*RAC1*	7	6441974	6441974	C	T	p.A178V	COSM1154840,COSM389868	Cancer	Diffuse	PMC
P06A-AAC	KRAS:12:	*KR*	12	2539	253	C	A	p.Q22H	COSM545	Adjacent	Diff	GMC

TCAGCATGTCCTC-1	25398253	*AS*		8253	98253					non-cancer	use	
P06A-ACATCAGTCAATAAGG-1	CCND1:11:69466021	*CCND1*	11	69466021	69466021	C	T	p.P287S	COSM4855094,COSM4855095,COSM931396	Adjacent non-cancer	Diffuse	PC
P06A-ACATGGTGTAGAGTGC-1	RAC1:7:6441974	*RAC1*	7	6441974	6441974	C	T	p.A178V	COSM1154840,COSM389868	Adjacent non-cancer	Diffuse	PC
P06A-ACGCAGCCATGGTCTA-1	RAC1:7:6441974	*RAC1*	7	6441974	6441974	C	T	p.A178V	COSM1154840,COSM389868	Adjacent non-cancer	Diffuse	PMC
P06A-ACTTGTTGTCATGCCG-1	RAC1:7:6441974	*RAC1*	7	6441974	6441974	C	T	p.A178V	COSM1154840,COSM389868	Adjacent non-cancer	Diffuse	PC

P06A-AGAGCTTGTTGCCTCT-1	RAC1:7:6441974	*RAC1*	7	6441974	6441974	C	T	p.A178V	COSM1154840,COSM389868	Adjacent non-cancer	Diffuse	GMC
P06A-AGGTCATGTTAGAACA-1	RAC1:7:6441974	*RAC1*	7	6441974	6441974	C	T	p.A178V	COSM1154840,COSM389868	Adjacent non-cancer	Diffuse	PC
P06A-AGTGTCATCATCATTC-1	RAC1:7:6441974	*RAC1*	7	6441974	6441974	C	T	p.A178V	COSM1154840,COSM389868	Adjacent non-cancer	Diffuse	PC
P06A-ATGGGAGAGATGTTAG-1	RAC1:7:6441974	*RAC1*	7	6441974	6441974	C	T	p.A178V	COSM1154840,COSM389868	Adjacent non-cancer	Diffuse	GMC
P06A-ATTCTACAGCAC	CCND1:11:69466021	*CCND1*	11	69466021	69466021	C	T	p.P287S	COSM4855094,COSM4855095,COSM931396	Adjacent non-cancer	Diffuse	GMC

CGTC-1					21							
P06A-CAGAGAGTCAGGTAAA-1	RAC1:7:6441974	*RAC1*	7	6441974	6441974	C	T	p.A178V	COSM1154840,COSM389868	Adjacent non-cancer	Diffuse	PMC
P06A-CGGACGTTCATGCTCC-1	RAC1:7:6441974	*RAC1*	7	6441974	6441974	C	T	p.A178V	COSM1154840,COSM389868	Adjacent non-cancer	Diffuse	GMC
P06A-CTCAGAAGTCATCGGC-1	RAC1:7:6441974	*RAC1*	7	6441974	6441974	C	T	p.A178V	COSM1154840,COSM389868	Adjacent non-cancer	Diffuse	PMC
P06A-CTCGTCACACGCGAAA-1	RAC1:7:6441974	*RAC1*	7	6441974	6441974	C	T	p.A178V	COSM1154840,COSM389868	Adjacent non-cancer	Diffuse	PMC
P06A-GAACGG	RAC1:7:6	*RAC1*	7	6441974	6441	C	T	p.A178V	COSM1154840,COSM389868	Adjacent no	Difu	Chief

ACAAGCCGCT-1	441974				974					n-cancer	se	
P06A-GACCTGGCAGACGTAG-1	RAC1:7:6441974	*RAC1*	7	6441974	6441974	C	T	p.A178V	COSM1154840,COSM389868	Adjacent non-cancer	Diffuse	PC
P06A-GCTCTGTAGCCAGTAG-1	RAC1:7:6441974	*RAC1*	7	6441974	6441974	C	T	p.A178V	COSM1154840,COSM389868	Adjacent non-cancer	Diffuse	GMC
P06A-GGCTCGAAGTACTTGC-1	RAC1:7:6441974	*RAC1*	7	6441974	6441974	C	T	p.A178V	COSM1154840,COSM389868	Adjacent non-cancer	Diffuse	GMC
P06A-GTATTCTCACATGTGT-1	CCND1:11:69466019	*CCND1*	11	69466019	69466019	C	T	p.T286I	COSM931395	Adjacent non-cancer	Diffuse	PMC

P06A-GTCTTCGGTCTAACC-1	CCND1:11:69466021	*CCND1*	11	69466021	69466021	C	T	p.P287S	COSM4855094,COSM4855095,COSM931396	Adjacent non-cancer	Diffuse	GMC
P06A-GTTCATTTCGTTACAG-1	RAC1:7:6441974	*RAC1*	7	6441974	6441974	C	T	p.A178V	COSM1154840,COSM389868	Adjacent non-cancer	Diffuse	GMC
P06A-TACAGTGCATCCAACA-1	RAC1:7:6441974	*RAC1*	7	6441974	6441974	C	T	p.A178V	COSM1154840,COSM389868	Adjacent non-cancer	Diffuse	PMC
P06A-TACTTGTCAACACGCC-1	RAC1:7:6441974	*RAC1*	7	6441974	6441974	C	T	p.A178V	COSM1154840,COSM389868	Adjacent non-cancer	Diffuse	GMC
P06A-TAGACCAGTCTC	RAC1:7:6441974	*RAC1*	7	6441974	6441974	C	T	p.A178V	COSM1154840,COSM389868	Adjacent non-cancer	Diffuse	PC

CCTA-1												
P06A-TAGCCGGCAAACCCAT-1	RAC1:7:6441974	*RAC1*	7	6441974	6441974	C	T	p.A178V	COSM1154840,COSM389868	Adjacent non-cancer	Difuse	Chief
P06A-TCTTCGGGTGAAAGAG-1	RAC1:7:6441974	*RAC1*	7	6441974	6441974	C	T	p.A178V	COSM1154840,COSM389868	Adjacent non-cancer	Difuse	GMC
P06A-TGCGGGTCAGTGAGTG-1	RAC1:7:6441974	*RAC1*	7	6441974	6441974	C	T	p.A178V	COSM1154840,COSM389868	Adjacent non-cancer	Difuse	PMC
P06A-TTGAACGTCTAACTCT-1	RAC1:7:6441974	*RAC1*	7	6441974	6441974	C	T	p.A178V	COSM1154840,COSM389868	Adjacent non-cancer	Difuse	Enteroendocrine
P06A-TTGACT	CCND1:11:6	*CCN*	11	69466022	6946	C	G	p.P287R	COSM2043470,COSM226265,COSM931397	Adjacent no	Difu	PMC

TTCTGTTTGT-1	946 602 2	*D1*			6022					n-cancer	se	
P06A-TTTGGTTAGGCCCTTG-1	CCND1:11:69466018	*CCND1*	11	69466018	69466018	A	G	p.T286A		Adjacent non-cancer	Diffuse	PMC
P06B-AACTGGTAGGTACTCT-1	RAC1:7:6441974	*RAC1*	7	6441974	6441974	C	T	p.A178V	COSM1154840,COSM389868	Cancer	Diffuse	Enteroendocrine
P06B-AGAGCTTAGTGAACGC-1	FBXW7:4:153244185	*FBXW7*	4	153244185	153244185	G	T	p.R658=	COSM1427626,COSM167197,COSM167198,COSM167199,COSM22967,COSM4837611,COSM4837612,COSM4837613,COSM4837614,COSM4837615	Cancer	Diffuse	GMC
P06B-GGGCACTGTTAAGATG-1	RAC1:7:6441974	*RAC1*	7	6441974	6441974	C	T	p.A178V	COSM1154840,COSM389868	Cancer	Diffuse	GMC

P06B-GTGAAGGGTTCCACAA-1	EP300:22:41525969	*EP300*	22	41525969	41525969	T	C	p.L415P	COSM221269	Cancer	Diffuse	Tumor
P07A-CCCATACTCCTTGACC-1	RAC1:7:6441974	*RAC1*	7	6441974	6441974	C	T	p.A178V	COSM1154840,COSM389868	Adjacent non-cancer	Intestinal	PMC
P07A-GTAACGTTCGCCAGCA-1	RAC1:7:6441974	*RAC1*	7	6441974	6441974	C	T	p.A178V	COSM1154840,COSM389868	Adjacent non-cancer	Intestinal	PC
P07B-AACTCCCCAGTTAACC-1	RAC1:7:6441974	*RAC1*	7	6441974	6441974	C	T	p.A178V	COSM1154840,COSM389868	Cancer	Intestinal	EC
P07B-ACACCA	CDK4:12:581	*CDK4*	12	58145436	5814	T	A	p.K22M	COSM3463915	Cancer	Inte	Tumor

ACAGGCTGAA-1	45436				5436						stinal	
P07B-ACGGGTCCACCGTTGG-1	CCND1:11:69466018	*CCND1*	11	69466018	69466018	A	G	p.T286A		Cancer	Intestinal	MSC
P07B-CTCACACCAACTGGCC-1	CCND1:11:69466021	*CCND1*	11	69466021	69466021	C	T	p.P287S	COSM4855094,COSM4855095,COSM931396	Cancer	Intestinal	Tumor
P07B-TATTACCTCAGGCCCA-1	CCND1:11:69466019	*CCND1*	11	69466019	69466019	C	T	p.T286I	COSM931395	Cancer	Intestinal	PC
P08A-AGTCTTTAGTACG	CCND1:11:69466022	*CCND1*	11	69466022	694660	C	G	p.P287R	COSM2043470,COSM226265,COSM931397	Adjacent non-cancer	Diffuse	EC

ACG-1					22							
P08A-ATCTACTCACATTCGA-1	CCND1:11:69466021	*CCND1*	11	69466021	69466021	C	T	p.P287S	COSM4855094,COSM4855095,COSM931396	Adjacent non-cancer	Diffuse	PMC
P08A-CCGGTAGCACTAAGTC-1	PDGFRA:4:55144146	*PDGFRA*	4	55144146	55144146	A	T	p.N659Y	COSM22416,COSM51516	Adjacent non-cancer	Diffuse	Fibroblast
P08A-CCTACACGTACTTCTT-1	RAC1:7:6441974	*RAC1*	7	6441974	6441974	C	T	p.A178V	COSM1154840,COSM389868	Adjacent non-cancer	Diffuse	EC
P08A-CGGAGCTGTACAGACG-1	RAC1:7:6441974	*RAC1*	7	6441974	6441974	C	T	p.A178V	COSM1154840,COSM389868	Adjacent non-cancer	Diffuse	EC
P08A-CTAGAG	CCND1:11:6	*CCN*	11	69466022	6946	C	G	p.P287R	COSM2043470,COSM226265,COSM931397	Adjacent no	Difu	EC

TAGGCGTACA-1	9466022	*D1*			6022					n-cancer	se	
P08A-CTAGAGTAGGCGTACA-1	RAC1:7:6441974	*RAC1*	7	6441974	6441974	C	T	p.A178V	COSM1154840,COSM389868	Adjacent non-cancer	Diffuse	EC
P08A-TACTTGTAGTACATGA-1	FBXW7:4:153244185	*FBXW7*	4	153244185	153244185	G	A	p.R658*	COSM1427626,COSM167197,COSM167198,COSM167199,COSM22967,COSM4837611,COSM4837612,COSM4837613,COSM4837614,COSM4837615	Adjacent non-cancer	Diffuse	EC
P08B-AGCTCTCCATGGTAGG-1	CCND1:11:69466021	*CCND1*	11	69466021	69466021	C	T	p.P287S	COSM4855094,COSM4855095,COSM931396	Cancer	Diffuse	MSC
P08B-CCTAAAGGTCGAATCT-1	CCND1:11:69466021	*CCND1*	11	69466021	69466021	C	A	p.P287T	COSM4855094,COSM4855095,COSM931396	Cancer	Diffuse	EC

P08B-GCAGTTAGTTCTGTTT-1	CCND1:11:69466022	*CCND1*	11	69466022	69466022	C	T	p.P287L	COSM2043470,COSM226265,COSM931397	Cancer	Diffuse	Tumor
P08B-GGCCGATGTCCGCTGA-1	CDKN2A:9:21970972	*CDKN2A*	9	21970972	21970972	T	C	p.Y129C	COSM13633	Cancer	Diffuse	Enteroendocrine
P08B-GGTGTTATCAACCATG-1	KRAS:12:25380275	*KRAS*	12	25380275	25380276	TT	GC	p.Q61R	COSM1168052	Cancer	Diffuse	Fibroblast
P08B-GGTGTTATCAACCATG-1	KRAS:12:25380275	*KRAS*	12	25380275	25380275	T	G	p.Q61H	rs17851045,COSM1135364,COSM1146992,COSM554,COSM555	Cancer	Diffuse	Fibroblast
P08B-GTCCTCAAGGAC	CCND1:11:69466022	*CCND1*	11	69466022	694660	C	T	p.P287L	COSM2043470,COSM226265,COSM931397	Cancer	Diffuse	Fibroblast

TGGT-1					22							
P08B-TATGCCCCAGCTCGCA-1	CCND1:11:69466018	*CCND1*	11	69466018	69466018	A	G	p.T286A		Cancer	Diffuse	MSC
P08B-TCGCGAGGTGCACTTA-1	SMARCA4:19:11106926	*SMARCA4*	19	11106926	11106928	AGA	-	p.K546del	COSM5576272,COSM5576273	Cancer	Diffuse	Fibroblast
P08B-TGGTTAGAGAAACCGC-1	CCND1:11:69466022	*CCND1*	11	69466022	69466022	C	G	p.P287R	COSM2043470,COSM226265,COSM931397	Cancer	Diffuse	Tumor
P09A-AAGACCTTCAGGTAAA-1	EIF1AX:X:20156713	*EIF1AX*	X	20156713	20156713	C	A	p.G15V	COSM3973543,COSM3973544	Adjacent non-cancer	Intestinal	MSC
P09A-AAG	RAC1:7:6	*RA*	7	6441	644	C	T	p.A1	COSM1154840,COSM389868	Adjacent	Int	PMC

GAGCGTGTGGTTT-1	441974	*C1*		974	1974			78V		non-cancer	estinal	
P09A-AAGTCTGTCCACTCCA-1	CCND1:11:69466021	*CCND1*	11	69466021	69466021	C	A	p.P287T	COSM4855094,COSM4855095,COSM931396	Adjacent non-cancer	Intestinal	PMC
P09A-ACGAGGAAGCCTTGAT-1	CCND1:11:69466022	*CCND1*	11	69466022	69466022	C	T	p.P287L	COSM2043470,COSM226265,COSM931397	Adjacent non-cancer	Intestinal	GMC
P09A-AGAGCGATCCCTAATT-1	RAC1:7:6441974	*RAC1*	7	6441974	6441974	C	T	p.A178V	COSM1154840,COSM389868	Adjacent non-cancer	Intestinal	GMC
P09A-AGCTCCTAGCAG	CREBBP:16:378	*CREBBP*	16	37886 05	37886	T	C	p.Y1450C	COSM88739	Adjacent non-	Intesti	GMC

GTCA-1	8605				05					cancer	nal	
P09A-ATCATGGCAAATACAG-1	RAC1:7:6441974	*RAC1*	7	6441974	6441974	C	T	p.A178V	COSM1154840,COSM389868	Adjacent non-cancer	Intestinal	GMC
P09A-ATTATCCGTTCATGGT-1	CCND1:11:69466021	*CCND1*	11	69466021	69466021	C	T	p.P287S	COSM4855094,COSM4855095,COSM931396	Adjacent non-cancer	Intestinal	PMC
P09A-CACAAACCAATGGTCT-1	VHL:3:10183734	*VHL*	3	10183734	10183734	C	A	p.S68*	rs869025617,CM003058,CM971566,COSM14372,COSM17870	Adjacent non-cancer	Intestinal	GMC
P09A-CACACAACACCTTGTC-1	RAC1:7:6441974	*RAC1*	7	6441974	6441974	C	T	p.A178V	COSM1154840,COSM389868	Adjacent non-cancer	Intestinal	GMC

P09A-CATCAAGAGACAGAGA-1	RAC1:7:6441974	*RAC1*	7	6441974	6441974	C	T	p.A178V	COSM1154840,COSM389868	Adjacent non-cancer	Intestinal	GMC
P09A-CCACCTAGTCTAGTCA-1	RAC1:7:6441974	*RAC1*	7	6441974	6441974	C	T	p.A178V	COSM1154840,COSM389868	Adjacent non-cancer	Intestinal	GMC
P09A-CCAGCGACAGTGGGAT-1	RAC1:7:6441974	*RAC1*	7	6441974	6441974	C	T	p.A178V	COSM1154840,COSM389868	Adjacent non-cancer	Intestinal	GMC
P09A-CCGTGGAAGGATGGAA-1	RAC1:7:6441974	*RAC1*	7	6441974	6441974	C	T	p.A178V	COSM1154840,COSM389868	Adjacent non-cancer	Intestinal	GMC
P09A-CGA	RAC1:7:6	*RA*	7	6441	644	C	T	p.A1	COSM1154840,COSM389868	Adjacent	Int	GMC

TCGGTCTTGCAAG-1	441974	*C1*		974	1974			78V		non-cancer	estinal	
P09A-CGCTTCACATGAAGTA-1	CCND1:11:69466021	*CCND1*	11	69466021	69466021	C	G	p.P287A	COSM4855094,COSM4855095,COSM931396	Adjacent non-cancer	Intestinal	GMC
P09A-CGCTTCACATGAAGTA-1	CCND1:11:69466018	*CCND1*	11	69466018	69466018	A	G	p.T286A		Adjacent non-cancer	Intestinal	GMC
P09A-CGTCTACCACCCGCTAG-1	RAC1:7:6441974	*RAC1*	7	6441974	6441974	C	T	p.A178V	COSM1154840,COSM389868	Adjacent non-cancer	Intestinal	Tumor
P09A-CTAGCCTGTCTA	ERBB3:12:5648	*ERBB3*	12	56482606	564826	A	C	p.T355P		Adjacent non-	Intesti	MSC

CCTC-1	2606				06					cancer	nal	
P09A-CTCTACGGTTGAGTTC-1	RAC1:7:6441974	*RAC1*	7	6441974	6441974	C	T	p.A178V	COSM1154840,COSM389868	Adjacent non-cancer	Intestinal	GMC
P09A-CTGTTTACAATCGAAA-1	CCND1:11:69466022	*CCND1*	11	69466022	69466022	C	G	p.P287R	COSM2043470,COSM226265,COSM931397	Adjacent non-cancer	Intestinal	PMC
P09A-GACGTGCTCGAGCCCA-1	FBXW7:4:153244092	*FBXW7*	4	153244092	153244092	G	A	p.R689W	COSM1154288,COSM206681,COSM206682,COSM206683,COSM27083,COSM5751359,COSM5751360,COSM5751361,COSM5751362,COSM5751363	Adjacent non-cancer	Intestinal	Chief
P09A-GCGAGAAAGCCCAATT-1	RAC1:7:6441974	*RAC1*	7	6441974	6441974	C	T	p.A178V	COSM1154840,COSM389868	Adjacent non-cancer	Intestinal	PMC

P09A-GCTGCAGAGAACAACT-1	RAC1:7:6441974	*RAC1*	7	6441974	6441974	C		T	p.A178V	COSM1154840,COSM389868	Adjacent non-cancer	Intestinal	GMC
P09A-GTGCAGCGTCAGTGGA-1	RAC1:7:6441974	*RAC1*	7	6441974	6441974	C		T	p.A178V	COSM1154840,COSM389868	Adjacent non-cancer	Intestinal	Chief
P09A-TATCAGGGTGTCGCTG-1	PTEN:10:89725043	*PTEN*	10	89725043	89725043	G		T	p.X343_splice	COSM1180410,COSM5962,COSM921160	Adjacent non-cancer	Intestinal	GMC
P09A-TGGGCGTTCTTGCAAG-1	RAC1:7:6441974	*RAC1*	7	6441974	6441974	C		T	p.A178V	COSM1154840,COSM389868	Adjacent non-cancer	Intestinal	GMC
P09B-ACG	RAC1:7:6	*RA*	7	6441	644	C		T	p.A1	COSM1154840,COSM389868	Cancer	Int	PC

GGTCTCCAAACTG-1	441974	*C1*		974	1974			78V			estinal	
P09B-AGCCTAATCAGCTTAG-1	RAC1:7:6441974	*RAC1*	7	6441974	6441974	C	T	p.A178V	COSM1154840,COSM389868	Cancer	Intestinal	MSC
P09B-AGTAGTCGTTCGCGAC-1	KRAS:12:25380276	*KRAS*	12	25380276	25380276	T	C	p.Q61R	rs121913240,COSM1140131,COSM1158660,COSM3688142,COSM551,COSM552,COSM553	Cancer	Intestinal	EC
P09B-AGTAGTCGTTCGCGAC-1	KRAS:12:25380276	*KRAS*	12	25380276	25380277	TG	GC	p.Q61A		Cancer	Intestinal	EC
P09B-CCACGGAAGCCC	RAC1:7:6441974	*RAC1*	7	6441974	64419	C	T	p.A178V	COSM1154840,COSM389868	Cancer	Intesti	MSC

GAAA-1					74						nal	
P09B-CTGATCCATTCCTGGC-1	RAC1:7:6441974	*RAC1*	7	6441974	6441974	C	T	p.A178V	COSM1154840,COSM389868	Cancer	Intestinal	MSC
P09B-GATCGCGAGCCCTAAT-1	RAC1:7:6441974	*RAC1*	7	6441974	6441974	C	T	p.A178V	COSM1154840,COSM389868	Cancer	Intestinal	MSC
P09B-GATCGCGTCACCTTAT-1	PTEN:10:89725053	*PTEN*	10	89725053	89725053	T	G	p.Y346D		Cancer	Intestinal	PC
P09B-GCAATCACAGCTGCAC-1	RAC1:7:6441974	*RAC1*	7	6441974	6441974	C	T	p.A178V	COSM1154840,COSM389868	Cancer	Intestinal	MSC

P09B-GCGCGATAGGGTTTCT-1	RAC1:7:6439806	*RAC1*	7	6439806	6439806	A	T	p.N111I	COSM1684687,COSM5624655,COSM5624656	Cancer	Intestinal	MSC
P09B-TCTTTCCCAGGATTGG-1	CCND1:11:69466022	*CCND1*	11	69466022	69466022	C	T	p.P287L	COSM2043470,COSM226265,COSM931397	Cancer	Intestinal	EC
P09B-TGCGGGTAGGTGTTAA-1	RAC1:7:6441974	*RAC1*	7	6441974	6441974	C	T	p.A178V	COSM1154840,COSM389868	Cancer	Intestinal	PMC
P10A-AGCTCCTCATAGTAAG-1	RHOA:3:49405933	*RHOA*	3	49405933	49405933	G	T	p.L69M		Adjacent non-cancer	Diffuse	Fibroblast
P10A-CGGACG	CREBBP:16:	*CREB*	16	3786715	3786	A	C	p.L1499R	COSM220497,COSM88752	Adjacent no	Difu	Goblet

TGTATGAATG-1	3786715	*BP*			715					n-cancer	se	
P10A-CGGACGTGTATGAATG-1	CREBBP:16:3786703	*CREBBP*	16	3786703	3786703	T	C	p.Y1503C	rs587783497,CM085345,COSM1161162,COSM88745	Adjacent non-cancer	Diffuse	Goblet
P10A-GTGAAGGGTCAACATC-1	CCND1:11:69466022	*CCND1*	11	69466022	69466022	C	G	p.P287R	COSM2043470,COSM226265,COSM931397	Adjacent non-cancer	Diffuse	PC
P10B-ACGAGGATCCGCGTTT-1	CCND1:11:69466022	*CCND1*	11	69466022	69466022	C	T	p.P287L	COSM2043470,COSM226265,COSM931397	Cancer	Diffuse	PC
P10B-CGTCACTTCGGCGCTA-1	SDHA:5:256470	*SDHA*	5	256470	256470	G	T	p.V644L	COSM6170729	Cancer	Diffuse	PMC

P10B-CTGATCCTCACTCTTA-1	EIF1AX:X:20156720	*EIF1AX*	X	20156720	20156720	G	C	p.R13G	COSM5899335	Cancer	Diffuse	MSC
P10B-GATCGTAAGTTCGCGC-1	KRAS:12:25380275	*KRAS*	12	25380275	25380276	TT	AA	p.Q61L	COSM1168052	Cancer	Diffuse	Tumor
P10B-GATCGTAAGTTCGCGC-1	KRAS:12:25380275	*KRAS*	12	25380275	25380275	T	A	p.Q61H	rs17851045,COSM1135364,COSM1146992,COSM554,COSM555	Cancer	Diffuse	Tumor
P10B-GGTATTGAGCCTTGAT-1	RAC1:7:6441974	*RAC1*	7	6441974	6441974	C	T	p.A178V	COSM1154840,COSM389868	Cancer	Diffuse	PC
P11A-CCACGGATCCCT	RAC1:7:6441974	*RAC1*	7	6441974	6441974	C	T	p.A178V	COSM1154840,COSM389868	Adjacent non-cancer	Diffuse	PMC

CTTT-1												
P11A-CCTTCCCGTCGACTGC-1	RAC1:7:6441974	*RAC1*	7	6441974	6441974	C	T	p.A178V	COSM1154840,COSM389868	Adjacent non-cancer	Diffuse	PMC
P11B-AAAGCAAAGGTTCCTA-1	KRAS:12:25378648	*KRAS*	12	25378648	25378648	T	C	p.K117R	COSM4696721,COSM4696722	Cancer	Diffuse	PC
P11B-CGTTAGACACGGATAG-1	RAC1:7:6439807	*RAC1*	7	6439807	6439807	T	A	p.N111K	COSM3640063,COSM3640064,COSM5038555,COSM5038556	Cancer	Diffuse	Fibroblast
P11B-TGAGAGGAGGACACCA-1	RAC1:7:6441974	*RAC1*	7	6441974	6441974	C	T	p.A178V	COSM1154840,COSM389868	Cancer	Diffuse	Tumor
P11B-TGGGAA	RAC1:7:6	*RAC1*	7	6441974	6441	C	T	p.A178V	COSM1154840,COSM389868	Cancer	Diffu	PMC

GAGGACACCA-1	441974				974						se	
P11B-TTTGGTTTCAAGAAGT-1	RAC1:7:6441974	*RAC1*	7	6441974	6441974	C	T	p.A178V	COSM1154840,COSM389868	Cancer	Diffuse	Chief
P12A-ACGCCGACAGATCGGA-1	RB1:13:48881414	*RB1*	13	48881414	48881414	A	G	p.X46_splice	CS040288,COSM5686958,COSM5686959	Adjacent non-cancer	Diffuse	EC
P12A-AGAGTGGGTACCATCA-1	RAC1:7:6441974	*RAC1*	7	6441974	6441974	C	T	p.A178V	COSM1154840,COSM389868	Adjacent non-cancer	Diffuse	Chief
P12A-ATGAGGGGTCAGCTAT-1	RAC1:7:6441974	*RAC1*	7	6441974	6441974	C	T	p.A178V	COSM1154840,COSM389868	Adjacent non-cancer	Diffuse	EC

P12A-ATTCTACTCAGCTCTC-1	RAC1:7:6441974	*RAC1*	7	6441974	6441974	C	T	p.A178V	COSM1154840,COSM389868	Adjacent non-cancer	Diffuse	Chief
P12A-ATTGGACGTACCTACA-1	CREBBP:16:3786705	*CREBBP*	16	3786705	3786705	C	G	p.W1502C	COSM1377824,COSM88753	Adjacent non-cancer	Diffuse	Fibroblast
P12A-ATTGGACGTACCTACA-1	CREBBP:16:3786707	*CREBBP*	16	3786707	3786707	A	T	p.W1502R	COSM5363732	Adjacent non-cancer	Diffuse	Fibroblast
P12A-CAAGGCCAGTGTGAAT-1	RAC1:7:6441974	*RAC1*	7	6441974	6441974	C	T	p.A178V	COSM1154840,COSM389868	Adjacent non-cancer	Diffuse	EC
P12A-GAACATCAGCAG	RAC1:7:6441974	*RAC1*	7	6441974	6441974	C	T	p.A178V	COSM1154840,COSM389868	Adjacent non-cancer	Diffuse	EC

GCTA-1												
P12A-GACCAATAGCCTCGTG-1	RAC1:7:6441974	*RAC1*	7	6441974	6441974	C	T	p.A178V	COSM1154840,COSM389868	Adjacent non-cancer	Diffuse	EC
P12A-GAGCAGAGTAGATTAG-1	RAC1:7:6441974	*RAC1*	7	6441974	6441974	C	T	p.A178V	COSM1154840,COSM389868	Adjacent non-cancer	Diffuse	Fibroblast
P12A-GCCTCTAGTTGCCTCT-1	PTEN:10:89720876	*PTEN*	10	89720876	89720876	G	A	p.X342_splice	rs786201041,CS043794,CS110216,COSM3441286,COSM5957,COSM5978	Adjacent non-cancer	Diffuse	PMC
P12A-GGCTCGACACGCTTTC-1	RAC1:7:6441974	*RAC1*	7	6441974	6441974	C	T	p.A178V	COSM1154840,COSM389868	Adjacent non-cancer	Diffuse	GMC
P12A-GGGACC	PTEN:10:897	*PTEN*	10	89720857	8972	C	A	p.Y336*	COSM5290,COSM5300	Adjacent no	Difu	PMC

TTCCGTTGTC-1	20857				0857					n-cancer	se	
P12A-TCAGGTACAAAGGTGC-1	RAC1:7:6439806	*RAC1*	7	6439806	6439806	A	T	p.N111I	COSM1684687,COSM5624655,COSM5624656	Adjacent non-cancer	Diffuse	Fibroblast
P12B-ACGGGCTAGGAATCGC-1	CCND1:11:69466022	*CCND1*	11	69466022	69466022	C	G	p.P287R	COSM2043470,COSM226265,COSM931397	Cancer	Diffuse	MSC
P12B-AGCAGCCGTGCCTGTG-1	RAC1:7:6441974	*RAC1*	7	6441974	6441974	C	T	p.A178V	COSM1154840,COSM389868	Cancer	Diffuse	EC
P12B-AGGTCCGAGAAACCGC-1	CCND1:11:69466021	*CCND1*	11	69466021	69466021	C	T	p.P287S	COSM4855094,COSM4855095,COSM931396	Cancer	Diffuse	MSC

P12B-CAAGATCGTCGGCTCA-1	PPP6C:9:127912061	*PPP6C*	9	127912061	127912061	G	A	p.S270L	COSM1167935,COSM228125	Cancer	Diffuse	MSC
P12B-CACACCTTCAGTACGT-1	RAC1:7:6441974	*RAC1*	7	6441974	6441974	C	T	p.A178V	COSM1154840,COSM389868	Cancer	Diffuse	EC
P12B-CATCGAAAGGCCGAAT-1	RAC1:7:6441974	*RAC1*	7	6441974	6441974	C	T	p.A178V	COSM1154840,COSM389868	Cancer	Diffuse	CAF
P12B-CTGCGGAGTGTGTGCC-1	CDKN2A:9:21971028	*CDKN2A*	9	21971028	21971028	C	T	p.W110*	rs121913389,CM060208,COSM12547,COSM126615,COSM126616,COSM1598222,COSM48297	Cancer	Diffuse	MSC
P12B-GATCGATTCCGA	FBXW7:4:153244185	*FBXW7*	4	153244185	1532444	G	A	p.R658*	COSM1427626,COSM167197,COSM167198,COSM167199,COSM22967,COSM4837611,COSM4837612,COSM4837613,COSM4837614,COSM4837615	Cancer	Diffuse	EC

GCCA-1					185							
P12B-GCTTCCACAACTGCGC-1	EP300:22:41566475	*EP300*	22	41566475	41566475	A	C	p.H1451P	COSM1034564,COSM1484264,COSM254672	Cancer	Diffuse	MSC
P12B-GGCTCGACAAGTCTAC-1	KRAS:12:25380279	*KRAS*	12	25380279	25380279	C	T	p.G60D	COSM1667041,COSM4531523,COSM548,COSM5879374,COSM87290	Cancer	Diffuse	MSC
P12B-GGGCATCGTCCGTGAC-1	CCND1:11:69466018	*CCND1*	11	69466018	69466018	A	G	p.T286A		Cancer	Diffuse	MSC
P12B-GGGTCTGTCAGGCGAA-1	CCND1:11:69466022	*CCND1*	11	69466022	69466022	C	G	p.P287R	COSM2043470,COSM226265,COSM931397	Cancer	Diffuse	EC
P12B-GTT	CCND1:1	*CCN*	11	6946	694	A	G	p.T2		Cancer	Diff	MSC

CTCGCAATGCCAT-1	1:69466018	*D1*		6018	66018			86A			use	
P12B-TACAGTGTCCTATTCA-1	RAC1:7:6441974	*RAC1*	7	6441974	6441974	C	T	p.A178V	COSM1154840,COSM389868	Cancer	Diffuse	MSC
P12B-TCTTTCCTCAGAGACG-1	CREBBP:16:3786705	*CREBBP*	16	3786705	3786705	C	G	p.W1502C	COSM1377824,COSM88753	Cancer	Diffuse	CAF
P12B-TGATTTCAGCACCGCT-1	PTEN:10:89725054	*PTEN*	10	89725054	89725054	A	G	p.Y346C		Cancer	Diffuse	CAF
P13A-ACATACGTCTTAGAGC-1	RAC1:7:6441974	*RAC1*	7	6441974	6441974	C	T	p.A178V	COSM1154840,COSM389868	Adjacent non-cancer	Intestinal	EC

P13A-ATTGGACGTCTAGGTT-1	NFE2L2:2:178098803	*NFE2L2*	2	178098803	178098803	C	T	p.G81D	COSM132957,COSM132961	Adjacent non-cancer	Intestinal	Fibroblast
P13A-CACAAACAGTATTGGA-1	CCND1:11:69466022	*CCND1*	11	69466022	69466022	C	G	p.P287R	COSM2043470,COSM226265,COSM931397	Adjacent non-cancer	Intestinal	EC
P13A-CGCCAAGCAAGTCTAC-1	CCND1:11:69466018	*CCND1*	11	69466018	69466018	A	G	p.T286A		Adjacent non-cancer	Intestinal	Chief
P13A-CTTACCGAGATGCGAC-1	CREBBP:16:3786715	*CREBBP*	16	3786715	3786715	A	C	p.L1499R	COSM220497,COSM88752	Adjacent non-cancer	Intestinal	EC
P13A-CTT	CCND1:1	*CCN*	11	6946	694	C	G	p.P2	COSM2043470,COSM226265,COSM931397	Adjacent	Int	EC

ACCGAGATGCGAC-1	1:69466022	*D1*		6022	66022			87R		non-cancer	estinal	
P13A-TCAGGTAAGCCACGTC-1	CCND1:11:69466022	*CCND1*	11	69466022	69466022	C	T	p.P287L	COSM2043470,COSM226265,COSM931397	Adjacent non-cancer	Intestinal	PC
P13B-ACCGTAATCGAACTGT-1	CDKN2A:9:21970971	*CDKN2A*	9	21970971	21970971	G	T	p.Y129*	COSM126614,COSM13221,COSM28562,COSM3788241	Cancer	Intestinal	MSC
P13B-CACAGGCAGCCCAAC-1	CCND1:11:69466021	*CCND1*	11	69466021	69466021	C	A	p.P287T	COSM4855094,COSM4855095,COSM931396	Cancer	Intestinal	PC
P13B-CAGTAACGTGCG	KRAS:12:25380278	*KRAS*	12	25380278	253802	A	C	p.G60=	COSM1159613,COSM1168050,COSM253757	Cancer	Intesti	MSC

AAAC-1					78						nal	
P13B-CCGTTCAGTAAGTTCC-1	PTEN:10:89720808	*PTEN*	10	89720808	89720808	T	C	p.L320S	CD110181,CM992427,CM033671,COSM28895,COSM35671	Cancer	Intestinal	Tumor
P13B-CGACCTTAGATCGGGT-1	RAC1:7:6441974	*RAC1*	7	6441974	6441974	C	T	p.A178V	COSM1154840,COSM389868	Cancer	Intestinal	PC
P13B-GCAAACTAGGCTCAGA-1	CDKN2A:9:21970969	*CDKN2A*	9	21970969	21970969	A	C	p.L130R	COSM13670,COSM18438,COSM28675,COSM33799,COSM3395738,COSM3395739,COSM3788239,COSM3788240,COSM4571148	Cancer	Intestinal	MSC
P13B-GCTCCTAAGCGTAATA-1	CDKN2A:9:21970969	*CDKN2A*	9	21970969	21970969	A	T	p.L130Q	COSM13670,COSM18438,COSM28675,COSM33799,COSM3395738,COSM3395739,COSM3788239,COSM3788240,COSM4571148	Cancer	Intestinal	PC

P13B-GTCGGGTTCACCAGGC-1	CDKN2A:9:21971036	*CDKN2A*	9	21971036	21971036	C	T	p.D108N	rs121913381,CM071585,CM973278,COSM12484,COSM1314728,COSM13489,COSM13520,COSM1674414,COSM753735,COSM753736,COSM753737,COSM753738	Cancer	Intestinal	MSC
P13B-GTCGGGTTCACCAGGC-1	KRAS:12:25378647	*KRAS*	12	25378647	25378647	T	A	p.K117N	COSM1256061,COSM1562192,COSM19940,COSM28519	Cancer	Intestinal	MSC
P13B-TATGCCCCAGGAATCG-1	CCND1:11:69466018	*CCND1*	11	69466018	69466018	A	G	p.T286A		Cancer	Intestinal	PC
P14A-ACGTCAAAGGACACCA-1	RAC1:7:6441974	*RAC1*	7	6441974	6441974	C	T	p.A178V	COSM1154840,COSM389868	Adjacent non-cancer	Diffuse	GMC
P14A-ACGTCA	TP53:17:7	*TP53*	17	7574018	7574	G	A	p.R337C	rs587782529,CM981929,TP53_g.16900C>T,COSM11071,COSM111628,COSM111629,COSM117591,C	Adjacent no	Difu	PC

ATCTATGTGG-1	574018				018				OSM1563605,COSM1563606,COSM235697	n-cancer	se		
P14A-CCTACCACACGGTAGA-1	RAC1:7:6441974	*RAC1*	7	6441974	6441974	C		T	p.A178V	COSM1154840,COSM389868	Adjacent non-cancer	Diffuse	PMC
P14A-CCTATTATCTCCGGTT-1	RAC1:7:6441974	*RAC1*	7	6441974	6441974	C		T	p.A178V	COSM1154840,COSM389868	Adjacent non-cancer	Diffuse	EC
P14A-CCTATTATCTCCGGTT-1	CCND1:11:69466018	*CCND1*	11	69466018	69466018	A		G	p.T286A		Adjacent non-cancer	Diffuse	EC
P14A-TCATTTGTCTCAAGTG-1	RAC1:7:6441974	*RAC1*	7	6441974	6441974	C		T	p.A178V	COSM1154840,COSM389868	Adjacent non-cancer	Diffuse	PMC

P14A-TTAGGCACATCGATGT-1	CCND1:11:69466021	*CCND1*	11	69466021	69466021	C	A	p.P287T	COSM4855094,COSM4855095,COSM931396	Adjacent non-cancer	Diffuse	GMC
P14B-ACAGCTACAGCGTTCG-1	CCND1:11:69466021	*CCND1*	11	69466021	69466021	C	T	p.P287S	COSM4855094,COSM4855095,COSM931396	Cancer	Diffuse	Tumor
P14B-ACTATCTGTTACAGAA-1	RAC1:7:6441974	*RAC1*	7	6441974	6441974	C	T	p.A178V	COSM1154840,COSM389868	Cancer	Diffuse	MSC
P14B-ACTTGTTCAAGCCGTC-1	PTEN:10:89720876	*PTEN*	10	89720876	89720876	G	T	p.X342_splice	CS043794,CS110216,COSM3441286,COSM5957,COSM5978	Cancer	Diffuse	Tumor
P14B-ACTTGTTCAAGC	PTEN:10:89720808	*PTEN*	10	89720808	897208	T	C	p.L320S	CD110181,CM992427,CM033671,COSM28895,COSM35671	Cancer	Diffuse	Tumor

CGTC-1					08							
P14B-ACTTGTTCAAGCCGTC-1	CCND1:11:69466022	*CCND1*	11	69466022	69466022	C	T	p.P287L	COSM2043470,COSM226265,COSM931397	Cancer	Diffuse	Tumor
P14B-AGACGTTTCAAGGTAA-1	FBXW7:4:153244185	*FBXW7*	4	153244185	153244185	G	A	p.R658*	COSM1427626,COSM167197,COSM167198,COSM167199,COSM22967,COSM4837611,COSM4837612,COSM4837613,COSM4837614,COSM4837615	Cancer	Diffuse	MSC
P14B-CATCGGGCACGGTTTA-1	KRAS:12:25398283	*KRAS*	12	25398283	25398283	A	C	p.G12=	COSM1159170,COSM3772370,COSM523,COSM524	Cancer	Diffuse	Tumor
P14B-CATTATCTCGGTGTCG-1	CCND1:11:69466022	*CCND1*	11	69466022	69466022	C	T	p.P287L	COSM2043470,COSM226265,COSM931397	Cancer	Diffuse	Tumor
P14B-CCT	CCND1:1	*CCN*	11	6946	694	C	G	p.P2	COSM2043470,COSM226265,COSM931397	Cancer	Diff	Tumor

TCCCAGACGCTTT-1	1:69466022	*D1*		6022	66022			87R			use	
P14B-CGAACATTCCCTAACC-1	RAC1:7:6441974	*RAC1*	7	6441974	6441974	C	T	p.A178V	COSM1154840,COSM389868	Cancer	Diffuse	CAF
P14B-CTAACTTAGCCCTAAT-1	CCND1:11:69466021	*CCND1*	11	69466021	69466021	C	T	p.P287S	COSM4855094,COSM4855095,COSM931396	Cancer	Diffuse	Tumor
P14B-CTACATTTCTGACCTC-1	CCND1:11:69466018	*CCND1*	11	69466018	69466018	A	G	p.T286A		Cancer	Diffuse	Tumor
P14B-GAGTCCGGTCGCATCG-1	RAC1:7:6441974	*RAC1*	7	6441974	6441974	C	T	p.A178V	COSM1154840,COSM389868	Cancer	Diffuse	Tumor

P14B-GATCGCGAGTCCCACG-1	KRAS:12:25378647	*KRAS*	12	25378647	25378647	T	G	p.K117N	rs770248150,COSM1256061,COSM1562192,COSM19940,COSM28519	Cancer	Diffuse	Tumor
P14B-GTAGGCCCAATGTTGC-1	CCND1:11:69466021	*CCND1*	11	69466021	69466021	C	T	p.P287S	COSM4855094,COSM4855095,COSM931396	Cancer	Diffuse	MSC
P14B-GTCCTCATCACCATAG-1	CCND1:11:69466021	*CCND1*	11	69466021	69466021	C	G	p.P287A	COSM4855094,COSM4855095,COSM931396	Cancer	Diffuse	Tumor
P14B-GTCTCGTTCCAGAGGA-1	CCND1:11:69466018	*CCND1*	11	69466018	69466018	A	G	p.T286A		Cancer	Diffuse	CAF
P14B-GTGCAGCTCTCC	CCND1:11:69466022	*CCND1*	11	69466022	694660	C	T	p.P287L	COSM2043470,COSM226265,COSM931397	Cancer	Diffuse	Tumor

TATA-1					22							
P14B-GTGCAGCTCTCCTATA-1	CCND1:11:69466022	*CCND1*	11	69466022	69466022	C	G	p.P287R	COSM2043470,COSM226265,COSM931397	Cancer	Diffuse	Tumor
P14B-TAGAGCTTCGGTTAAC-1	VHL:3:10188208	*VHL*	3	10188208	10188208	G	C	p.W117C	CM951286,HM971481,COSM14328,COSM14399,COSM17964,COSM479172	Cancer	Diffuse	Tumor
P14B-TGCCCATTCGAACTGT-1	CCND1:11:69466022	*CCND1*	11	69466022	69466022	C	G	p.P287R	COSM2043470,COSM226265,COSM931397	Cancer	Diffuse	Tumor
P15A-AACCATGAGTCCATAC-1	RAC1:7:6441974	*RAC1*	7	6441974	6441974	C	T	p.A178V	COSM1154840,COSM389868	Adjacent non-cancer	Diffuse	Chief
P15A-ACGCCA	DNMT3A:2:2	*DNMT*	2	25457243	2545	G	A	p.R882C	rs377577594,COSM1166704,COSM1583136,COSM4383521,COSM53042,COSM87001	Adjacent no	Difu	GMC

GGTCTAGTGT-1	5457243	*3A*			7243					n-cancer	se	
P15A-ACTGATGCAGGATCGA-1	CCND1:11:69466021	*CCND1*	11	69466021	69466021	C	A	p.P287T	COSM4855094,COSM4855095,COSM931396	Adjacent non-cancer	Diffuse	PC
P15A-AGGCCGTAGGCCCTCA-1	RAC1:7:6441974	*RAC1*	7	6441974	6441974	C	T	p.A178V	COSM1154840,COSM389868	Adjacent non-cancer	Diffuse	Chief
P15A-ATTGGACGTTTCCACC-1	RAC1:7:6439807	*RAC1*	7	6439807	6439807	T	G	p.N111K	COSM3640063,COSM3640064,COSM5038555,COSM5038556	Adjacent non-cancer	Diffuse	Chief
P15A-CAGCAGCCATACGCCG-1	RAC1:7:6441974	*RAC1*	7	6441974	6441974	C	T	p.A178V	COSM1154840,COSM389868	Adjacent non-cancer	Diffuse	GMC

P15A-CCTTACGCATTATCTC-1	CCND1:11:69466018	*CCND1*	11	69466018	69466018	A	G	p.T286A		Adjacent non-cancer	Diffuse	GMC
P15A-CTCACACTCTTGAGAC-1	CCND1:11:69466021	*CCND1*	11	69466021	69466021	C	A	p.P287T	COSM4855094,COSM4855095,COSM931396	Adjacent non-cancer	Diffuse	PC
P15A-CTCGGGAGTGCGCTTG-1	CCND1:11:69466021	*CCND1*	11	69466021	69466021	C	G	p.P287A	COSM4855094,COSM4855095,COSM931396	Adjacent non-cancer	Diffuse	Chief
P15A-CTGTTTAAGGTGCAAC-1	CCND1:11:69466018	*CCND1*	11	69466018	69466018	A	G	p.T286A		Adjacent non-cancer	Diffuse	PMC
P15A-GATCTAGTCTAC	CCND1:11:69466022	*CCND1*	11	69466022	694660	C	T	p.P287L	COSM2043470,COSM226265,COSM931397	Adjacent non-cancer	Diffuse	PC

TCAT-1					22							
P15A-GATCTAGTCTACTCAT-1	RAC1:7:6439806	*RAC1*	7	6439806	6439806	A	G	p.N111S	COSM1684687,COSM5624655,COSM5624656	Adjacent non-cancer	Diffuse	PC
P15A-GCTGGGTAGCAGCGTA-1	CCND1:11:69466019	*CCND1*	11	69466019	69466019	C	T	p.T286I	COSM931395	Adjacent non-cancer	Diffuse	PC
P15A-TACTCATTCAAACCGT-1	PTEN:10:89711902	*PTEN*	10	89711902	89711902	T	C	p.Y174H	COSM28897,COSM5221	Adjacent non-cancer	Diffuse	GMC
P15A-TGAAAGAGTAGGACAC-1	RAC1:7:6441974	*RAC1*	7	6441974	6441974	C	T	p.A178V	COSM1154840,COSM389868	Adjacent non-cancer	Diffuse	GMC
P15B-AGCGTA	RAC1:7:6	*RAC1*	7	6441974	6441	C	T	p.A178V	COSM1154840,COSM389868	Cancer	Diffu	GMC

TGTTCACGGC-1	441974				974						se	
P15B-AGCTCTCCACCAGGCT-1	RAC1:7:6439807	*RAC1*	7	6439807	6439807	T	G	p.N111K	COSM3640063,COSM3640064,COSM5038555,COSM5038556	Cancer	Diffuse	MSC
P15B-CAGATCAGTTCGGCAC-1	CCND1:11:69466022	*CCND1*	11	69466022	69466022	C	T	p.P287L	COSM2043470,COSM226265,COSM931397	Cancer	Diffuse	MSC
P15B-CATGACATCGCACTCT-1	CCND1:11:69466022	*CCND1*	11	69466022	69466022	C	T	p.P287L	COSM2043470,COSM226265,COSM931397	Cancer	Diffuse	MSC
P15B-GCACATAGTGCCTGTG-1	RAC1:7:6441974	*RAC1*	7	6441974	6441974	C	T	p.A178V	COSM1154840,COSM389868	Cancer	Diffuse	MSC

P15B-GCTCTGTAGTGGGATC-1	RAC1:7:6441974	*RAC1*	7	6441974	6441974	C	T	p.A178V	COSM1154840,COSM389868	Cancer	Diffuse	MSC
P15B-GGGTCTGTCTGCGTAA-1	PTPN11:12:112888189	*PTPN11*	12	112888189	112888189	G	A	p.E69K	rs397507511,CM030493,COSM13013,COSM6006328	Cancer	Diffuse	GMC
P15B-TGACTAGGTCGGCTCA-1	RAC1:7:6441974	*RAC1*	7	6441974	6441974	C	T	p.A178V	COSM1154840,COSM389868	Cancer	Diffuse	PC
P15B-TGAGAGGCAGCTGTTA-1	RAC1:7:6441974	*RAC1*	7	6441974	6441974	C	T	p.A178V	COSM1154840,COSM389868	Cancer	Diffuse	GMC
P16A-ATTACTCAGCGC	CDKN2A:9:2197	*CDKN2A*	9	21970972	219709	T	C	p.Y129C	COSM13633	Adjacent non-	Diffuse	Enteroendoc

CTCA-1	0972				72					cancer		rine
P16A-CGTTGGGCATACGCCG-1	RAC1:7:6441974	*RAC1*	7	6441974	6441974	C	T	p.A178V	COSM1154840,COSM389868	Adjacent non-cancer	Diffuse	EC
P16A-TACCTTACATTAACCG-1	RAC1:7:6441974	*RAC1*	7	6441974	6441974	C	T	p.A178V	COSM1154840,COSM389868	Adjacent non-cancer	Diffuse	Tumor
P16A-TCAACGATCAGGCCCA-1	CCND1:11:69466022	*CCND1*	11	69466022	69466022	C	T	p.P287L	COSM2043470,COSM226265,COSM931397	Adjacent non-cancer	Diffuse	PC
P16B-AAAGATGCAGCCACCA-1	CCND1:11:69466022	*CCND1*	11	69466022	69466022	C	G	p.P287R	COSM2043470,COSM226265,COSM931397	Cancer	Diffuse	MSC
P16B-ACGAGC	RAC1:7:6	*RAC1*	7	6441974	6441	C	T	p.A178V	COSM1154840,COSM389868	Cancer	Diffu	Tumor

CCAGACAAGC-1	441974				974						se	
P16B-AGATCTGTCGTTTAGG-1	RAC1:7:6441974	*RAC1*	7	6441974	6441974	C	T	p.A178V	COSM1154840,COSM389868	Cancer	Diffuse	MSC
P16B-AGCCTAAGTAAACCTC-1	CCND1:11:69466021	*CCND1*	11	69466021	69466021	C	T	p.P287S	COSM4855094,COSM4855095,COSM931396	Cancer	Diffuse	MSC
P16B-AGCGGTCGTGAACCTT-1	RAC1:7:6441974	*RAC1*	7	6441974	6441974	C	T	p.A178V	COSM1154840,COSM389868	Cancer	Diffuse	MSC
P16B-AGGCCACTCTAACGGT-1	SMARCA4:19:11106926	*SMARCA4*	19	11106926	11106928	AGA	-	p.K546del	COSM5576272,COSM5576273	Cancer	Diffuse	MSC

P16B-CACATAGGTGTAATGA-1	CCND1:11:69466022	*CCND1*	11	69466022	69466022	C	T	p.P287L	COSM2043470,COSM226265,COSM931397	Cancer	Diffuse	MSC
P16B-CACCTTGTCTGCAGTA-1	CCND1:11:69466021	*CCND1*	11	69466021	69466021	C	G	p.P287A	COSM4855094,COSM4855095,COSM931396	Cancer	Diffuse	MSC
P16B-CACCTTGTCTGCAGTA-1	CCND1:11:69466022	*CCND1*	11	69466022	69466022	C	G	p.P287R	COSM2043470,COSM226265,COSM931397	Cancer	Diffuse	MSC
P16B-CAGCAGCTCTGTCTAT-1	RAC1:7:6441974	*RAC1*	7	6441974	6441974	C	T	p.A178V	COSM1154840,COSM389868	Cancer	Diffuse	MSC
P16B-CCACCTATCCAA	CCND1:11:69466022	*CCND1*	11	69466022	694660	C	T	p.P287L	COSM2043470,COSM226265,COSM931397	Cancer	Diffuse	MSC

ATGC-1					22							
P16B-CCTAGCTCACGTTGGC-1	RAC1:7:6439807	*RAC1*	7	6439807	6439807	T	A	p.N111K	COSM3640063,COSM3640064,COSM5038555,COSM5038556	Cancer	Diffuse	MSC
P16B-CCTCTGATCTCGCTTG-1	RAC1:7:6441974	*RAC1*	7	6441974	6441974	C	T	p.A178V	COSM1154840,COSM389868	Cancer	Diffuse	MSC
P16B-CGCTATCTCAAACGGG-1	RAC1:7:6441974	*RAC1*	7	6441974	6441974	C	T	p.A178V	COSM1154840,COSM389868	Cancer	Diffuse	MSC
P16B-GATCGCGGTGTGAATA-1	RAC1:7:6441974	*RAC1*	7	6441974	6441974	C	T	p.A178V	COSM1154840,COSM389868	Cancer	Diffuse	Tumor
P16B-GATCTA	RAC1:7:6	*RAC1*	7	6441974	6441	C	T	p.A178V	COSM1154840,COSM389868	Cancer	Diffu	PC

GAGTACGCGA-1	441974				974						se	
P16B-GCATGTAGTAGTAGTA-1	RAC1:7:6441974	*RAC1*	7	6441974	6441974	C	T	p.A178V	COSM1154840,COSM389868	Cancer	Diffuse	MSC
P16B-GCGACCACATTACGAC-1	RAC1:7:6441974	*RAC1*	7	6441974	6441974	C	T	p.A178V	COSM1154840,COSM389868	Cancer	Diffuse	MSC
P16B-GGCGTGTTCTGTTTGT-1	EP300:22:41566488	*EP300*	22	41566488	41566488	G	A	p.Q1455=	COSM1308207,COSM4387471	Cancer	Diffuse	MSC
P16B-GGGATGATCTTCGGTC-1	RAC1:7:6441974	*RAC1*	7	6441974	6441974	C	T	p.A178V	COSM1154840,COSM389868	Cancer	Diffuse	PC

P16B-GTACGTAGTTTGTTTC-1	GNAS:20:57484595	*GNAS*	20	57484595	57484595	C	A	p.Q227K	rs797045203,COSM28618,COSM4962872,COSM4962873,COSM5352260	Cancer	Diffuse	PC
P16B-GTGCATAAGCCCAACC-1	CCND1:11:69466021	*CCND1*	11	69466021	69466021	C	A	p.P287T	COSM4855094,COSM4855095,COSM931396	Cancer	Diffuse	MSC
P16B-TAAGCGTAGCGTAGTG-1	RAC1:7:6441974	*RAC1*	7	6441974	6441974	C	T	p.A178V	COSM1154840,COSM389868	Cancer	Diffuse	MSC
P16B-TCACGAACACCAACCG-1	RAC1:7:6441974	*RAC1*	7	6441974	6441974	C	T	p.A178V	COSM1154840,COSM389868	Cancer	Diffuse	MSC
P16B-TCGCGAGGTCAC	RAC1:7:6439807	*RAC1*	7	6439807	6439807	T	A	p.N111K	COSM3640063,COSM3640064,COSM5038555,COSM5038556	Cancer	Diffuse	MSC

TGGC-1												
P16B-TGCACCTCAGAGCCAA-1	KRAS:12:25380282	*KRAS*	12	25380282	25380282	G	T	p.A59E	COSM1135365,COSM1318029,COSM28518,COSM547	Cancer	Diffuse	GMC
P16B-TTGCGTCGTACAGTGG-1	SMAD2:18:45368213	*SMAD2*	18	45368213	45368213	G	T	p.C463*		Cancer	Diffuse	PC
P17A-AACTCTTAGTAGCCGA-1	CCND1:11:69466022	*CCND1*	11	69466022	69466022	C	T	p.P287L	COSM2043470,COSM226265,COSM931397	Adjacent non-cancer	Intestinal	MSC
P17A-CCGTTCAAGGATCGCA-1	RB1:13:48954300	*RB1*	13	48954300	48954300	G	A	p.X474_splice	CS058009	Adjacent non-cancer	Intestinal	EC

P17B-AAGTCTGCACTGAAGG-1	CDKN2A:9:21970972	*CDKN2A*	9	21970972	21970972	T	C	p.Y129C	COSM13633	Cancer	Intestinal	PC
P17B-ACAGCTAAGGAGCGAG-1	CDKN2A:9:21970987	*CDKN2A*	9	21970987	21971685	CGATGGCCCAGCTCCTCAGCCAGGTCCACGGGCAGACGGCCCCAGGCATCGCGCACGTCCAGCCGCGCCCCGGCCCGGTGCAGCACCACCAGCGTGTCCAGGAAGCCCTCCCGGGCAGCGTCGTGCACGGGTCGGGTGAGAGTGGCGGGGTCGGCGCAGTTGGGCTCCGCGCCGTGGAGCAGCAGCAGCTCCGCCACTCGGGCGCTGCCCATCATCATGACCTGCCAGAGAGAACAGAATGGTCAGAGCCAGGGTGGGGGCCGGCATGACGGAAAGGAAGCTTGTGTAGAGCCCCCTCACCGCCAAGCAGACCCCCACACAAGCCCCAGGTGTCTAATTACCCCTACATTTGCTTCCAGTTTCCAATTTCCTTCTTGAGTTCTCTATCCATTCTTCAGTACACAATGAATTCCATTATATCCTCCGAACTTCTGCGGAGCTGTCGTCACAGGCAGAGAGCACTGTGAGGCACGGGCAAAATAGCAAAGGGGCAGGGACAGACTGACTTTTACTCCAGGCTAACTTCCTGTATTTCCCCTGAGATACAACTACTGAAATTTCTTCCTGAAATTATGTTAGGCCTGGAGATTTTTTTTTTTTTTTTTGTTCACTGCTGTATATCCAAGCGCAGAATGTGGTAATTGTTAAAAAGAGAAAACTTGTTTGTTTGTTAAAACAAATTCTCACAAAACTTTTAAG	-	p.X51_splice		Cancer	Intestinal	PC
P17B-ACAGCTAAGGAGCGAG-1	CDKN2A:9:21970969	*CDKN2A*	9	21970969	21970969	A	C	p.L130R	COSM13670,COSM18438,COSM28675,COSM33799,COSM3395738,COSM3395739,COSM3788239,COSM3788240,COSM4571148	Cancer	Intestinal	PC

P17B-ACGGGCTTCTTGCCGT-1	CDKN2A:9:21968242	*CDKN2A*	9	21968242	21968242	C	G	p.X153_splice	CS127044,COSM21562,COSM3952628,COSM99937	Cancer	Intestinal	PC
P17B-ACGGGCTTCTTGCCGT-1	CCND1:11:69466022	*CCND1*	11	69466022	69466022	C	T	p.P287L	COSM2043470,COSM226265,COSM931397	Cancer	Intestinal	PC
P17B-ACGGGCTTCTTGCCGT-1	CDKN2A:9:21970969	*CDKN2A*	9	21970969	21970969	A	C	p.L130R	COSM13670,COSM18438,COSM28675,COSM33799,COSM3395738,COSM3395739,COSM3788239,COSM3788240,COSM4571148	Cancer	Intestinal	PC
P17B-ACGGGCTTCTTGCCGT-1	CDKN2A:9:21968242	*CDKN2A*	9	21968242	21968243	-	A	p.X153_splice		Cancer	Intestinal	PC
P17B-ACG	CDKN2A:	*CDK*	9	2197	219	C	A	p.D1	rs121913381,CM071585,CM973278,COSM12484,COSM1314728,COSM13489,COSM13520,COSM1674	Cancer	Int	PC

GGCTTCTTGCCGT-1	9:21971036	*N2A*		1036	71036			08Y	414,COSM753735,COSM753736,COSM753737,COSM753738		estinal	
P17B-ACGGGCTTCTTGCCGT-1	PPP6C:9:127912080	*PPP6C*	9	127912080	127912080	G	A	p.R264C	rs763733111,COSM1151204,COSM221754	Cancer	Intestinal	PC
P17B-AGATCTGAGTCCTCCT-1	CDKN2A:9:21971035	*CDKN2A*	9	21971035	21971035	T	C	p.D108G	COSM1638187,COSM4767456,COSM4767457,COSM4767458,COSM4767459,COSM753739,COSM753740,COSM753741	Cancer	Intestinal	PC
P17B-AGATCTGAGTCCTCCT-1	CDKN2A:9:21971028	*CDKN2A*	9	21971028	21971028	C	T	p.W110*	rs121913389,CM060208,COSM12547,COSM126615,COSM126616,COSM1598222,COSM48297	Cancer	Intestinal	PC
P17B-AGATCTGAGTCC	CDKN2A:9:2197	*CDKN2A*	9	21971036	219710	C	A	p.D108Y	rs121913381,CM071585,CM973278,COSM12484,COSM1314728,COSM13489,COSM13520,COSM1674414,COSM753735,COSM753736,COSM753737,COSM753738	Cancer	Intesti	PC

TCCT-1	1036				36						nal	
P17B-AGCGTATGTAGCGTCC-1	RAC1:7:6441974	*RAC1*	7	6441974	6441974	C	T	p.A178V	COSM1154840,COSM389868	Cancer	Intestinal	PC
P17B-AGGGATGAGTGTACCT-1	CDKN2A:9:21970969	*CDKN2A*	9	21970969	21970969	A	T	p.L130Q	COSM13670,COSM18438,COSM28675,COSM33799,COSM3395738,COSM3395739,COSM3788239,COSM3788240,COSM4571148	Cancer	Intestinal	PC
P17B-AGGGATGAGTGTACCT-1	CDKN2A:9:21971028	*CDKN2A*	9	21971028	21971028	C	T	p.W110*	rs121913389,CM060208,COSM12547,COSM126615,COSM126616,COSM1598222,COSM48297	Cancer	Intestinal	PC
P17B-AGTCTTTCACGGCTAC-1	CDKN2A:9:21970969	*CDKN2A*	9	21970969	21970969	A	T	p.L130Q	COSM13670,COSM18438,COSM28675,COSM33799,COSM3395738,COSM3395739,COSM3788239,COSM3788240,COSM4571148	Cancer	Intestinal	PC

P17B-AGTGGGAAGAGAACAG-1	CDKN2A:9:21970969	*CDKN2A*	9	21970969	21970969	A	C	p.L130R	COSM13670,COSM18438,COSM28675,COSM33799,COSM3395738,COSM3395739,COSM3788239,COSM3788240,COSM4571148	Cancer	Intestinal	PC
P17B-ATAAGAGGTTGTGGAG-1	CDKN2A:9:21968242	*CDKN2A*	9	21968242	21968242	C	T	p.X153_splice	CS127044,COSM21562,COSM3952628,COSM99937	Cancer	Intestinal	PC
P17B-ATAAGAGGTTGTGGAG-1	CDKN2A:9:21968242	*CDKN2A*	9	21968242	21968243	-	A	p.X153_splice		Cancer	Intestinal	PC
P17B-ATGAGGGCACCAGGCT-1	CDKN2A:9:21970987	*CDKN2A*	9	21970987	21971685	CGATGGCCCAGCTCCTCAGCCAGGTCCACGGGCAGACGGCCCCAGGCATCGCGCACGTCCAGCCGCGCCCCGGCCCGGTGCAGCACCACCAGCGTGTCCAGGAAGCCCTCCCGGGCAGCGTCGTGCACGGGTCGGGTGAGAGTGGCGGGGTCGGCGCAGTTGGGCTCCGCGCCGTGGAGCAGCAGCAGCTCCGCCACTCGGGCGCTGCCCATCATCATGACCTGCCAGAGAGAACAGAATGGTCAGAGCCAGGGTGGGGGCCGGCATGACGGAAAGGAAGCTTGTGTAGAGCCCCCTCACCGCCAAGCAGACCCCCACACAAGCCCCAGGTGTCTAATTACCCCTACATTTGCTTCCAGTTTCCAATTTCCTTCTTGAGTTCTCTATCCATTCTTCAGTACACAATGAATTCCATTATATCCTCCGAACTTCTGCGGAGCTGTCGTCACAGGCAGAGAGCAC	-	p.X51_splice		Cancer	Intestinal	PC

						TGTGAGGCACGGGCAAAATAGCAAAGGGGCAGGGACAGACTGACTTTTACTCCAGGCTAACTTCCTGTATTTCCCCTGAGATACAACTACTGAAATTTCTTCCTGAAATTATGTTAGGCCTGGAGATTTTTTTTTTTTTTTTTTGTTCACTGCTGTATATCCAAGCGCAGAATGTGGTAATTGTTAAAAAGAGAAAACTTGTTTGTTTGTTAAAACAAATTCTCACAAAACTTTTAAG						
P17B-ATGAGGGCACCAGGCT-1	CDKN2A:9:21971028	*CDKN2A*	9	21971028	21971028	C	T	p.W110*	rs121913389,CM060208,COSM12547,COSM126615,COSM126616,COSM1598222,COSM48297	Cancer	Intestinal	PC
P17B-ATGAGGGCACCAGGCT-1	CDKN2A:9:21971029	*CDKN2A*	9	21971029	21971029	C	T	p.W110*	rs1057519852,COSM12481,COSM126617,COSM126618,COSM3382498	Cancer	Intestinal	PC
P17B-ATGAGGGCACCAGGCT-1	SMARCA4:19:11106926	*SMARCA4*	19	11106926	11106928	AGA	-	p.K546del	COSM5576272,COSM5576273	Cancer	Intestinal	PC
P17B-ATGAGGGCA	CDKN2A:9:2197	*CDKN*	9	21970987	21971	CGATGGCCCAGCTCCTCAGCCAGGTCCACGGGCAGACGGCCCCAGGCATCGCGCACGTCCAGCCGCGCCCCGGCCCGGTGCAGCACCACCAGCGTGTCCAGGAAGCCCTCCCGGGCAGCGTCGTGCACGGGTCGGGTGAGAGTGGCGGGGTCGGCGCAGTTGGGCTCCGCGCCGTGGAGCAGCAGCAGCT	-	p.X51_splice		Cancer	Intes	PC

GCT GTA T-1	098 7	*2 A*			6 8 5	CCGCCACTCGGGCGCTGCCCATCATCATGACCTGCCAGAGAGAACAGAATGGTCAGAGCCAGGGTGGGGGCCGGCATGACGGAAAGGAAGCTTGTGTAGAGCCCCCTCACCGCCAAGCAGACCCCCACACAAGCCCCAGGTGTCTAATTACCCCTACATTTGCTTCCAGTTTCCAATTTCCTTCTTGAGTTCTCTATCCATTCTTCAGTACACAATGAATTCCATTATATCCTCCGAACTTCTGCGGAGCTGTCGTCACAGGCAGAGAGCACTGTGAGGCACGGGCAAAATAGCAAAGGGGCAGGGACAGACTGACTTTTACTCCAGGCTAACTTCCTGTATTTCCCCTGAGATACAACTACTGAAATTTCTTCCTGAAATTATGTTAGGCCTGGAGATTTTTTTTTTTTTTTTTGTTCACTGCTGTATATCCAAGCGCAGAATGTGGTAATTGTTAAAAAGAGAAAACTTGTTTGTTTGTTAAAACAAATTCTCACAAAACTTTTAAG					ti n a l	
P17B - ATG AGG GCA GCT GTA T-1	CD KN 2A: 9:2 197 096 9	*C D K N 2 A*	9	21 97 09 69	2 1 9 7 0 9 6 9	A	T	p. L1 30 Q	COSM13670,COSM18438,COSM28675,COSM33799,COSM3395738,COSM3395739,COSM3788239,COSM3788240,COSM4571148	Ca nce r	I n t e s ti n a l	P C
P17B - ATG AGG GCA GCT GTA T-1	CD KN 2A: 9:2 197 097 1	*C D K N 2 A*	9	21 97 09 71	2 1 9 7 0 9 7 1	G	C	p. Y1 29 *	COSM126614,COSM13221,COSM28562,COSM3788241	Ca nce r	I n t e s ti n a l	P C
P17B - ATG AGG GCA GCT	CD KN 2A: 9:2 197 103 5	*C D K N 2 A*	9	21 97 10 35	2 1 9 7 1 0	T	C	p. D1 08 G	COSM1638187,COSM4767456,COSM4767457,COSM4767458,COSM4767459,COSM753739,COSM753740,COSM753741	Ca nce r	I n t e s ti n	P C

GTAT-1					35						al	
P17B-CACAGGCGTCGCTTCT-1	CDKN2A:9:21971035	*CDKN2A*	9	21971035	21971035	T	C	p.D108G	COSM1638187,COSM4767456,COSM4767457,COSM4767458,COSM4767459,COSM753739,COSM753740,COSM753741	Cancer	Intestinal	PC
P17B-CACAGGCGTCGCTTCT-1	CDKN2A:9:21970971	*CDKN2A*	9	21970971	21970971	G	C	p.Y129*	COSM126614,COSM13221,COSM28562,COSM3788241	Cancer	Intestinal	PC
P17B-CACAGGCGTCGCTTCT-1	CCND1:11:69466021	*CCND1*	11	69466021	69466021	C	G	p.P287A	COSM4855094,COSM4855095,COSM931396	Cancer	Intestinal	PC
P17B-CACTCCAGTTCGGCAC-1	CDKN2A:9:21970971	*CDKN2A*	9	21970971	21970971	G	T	p.Y129*	COSM126614,COSM13221,COSM28562,COSM3788241	Cancer	Intestinal	PC

P17B-CAGCATAAGTCCTCCT-1	CDKN2A:9:21970987	*CDKN2A*	9	21970987	21971685	CGATGGCCCAGCTCCTCAGCCAGGTCCACGGGCAGACGGCCCCAGGCATCGCGCACGTCCAGCCGCGCCCCGGCCCGGTGCAGCACCACCAGCGTGTCCAGGAAGCCCTCCCGGGCAGCGTCGTGCACGGGTCGGGTGAGAGTGGCGGGGTCGGCGCAGTTGGGCTCCGCGCCGTGGAGCAGCAGCAGCTCCGCCACTCGGGCGCTGCCCATCATCATGACCTGCCAGAGAGAACAGAATGGTCAGAGCCAGGGTGGGGGCCGGCATGACGGAAAGGAAGCTTGTGTAGAGCCCCCTCACCGCCAAGCAGACCCCCACACAAGCCCCAGGTGTCTAATTACCCCTACATTTGCTTCCAGTTTCCAATTTCCTTCTTGAGTTCTCTATCCATTCTTCAGTACACAATGAATTCCATTATATCCTCCGAACTTCTGCGGAGCTGTCGTCACAGGCAGAGAGCACTGTGAGGCACGGGCAAAATAGCAAAGGGGCAGGGACAGACTGACTTTTACTCCAGGCTAACTTCCTGTATTTCCCCTGAGATACAACTACTGAAATTTCTTCCTGAAATTATGTTAGGCCTGGAGATTTTTTTTTTTTTTTTGTTCACTGCTGTATATCCAAGCGCAGAATGTGGTAATTGTTAAAAAGAGAAAACTTGTTTGTTTGTTAAAACAAATTCTCACAAAACTTTTAAG	-	p.X51_splice		Cancer	Intestinal	PC
P17B-CATATGGAGTACTTGC-1	CDKN2A:9:21971000	*CDKN2A*	9	21971000	21971000	C	A	p.E120*	CD972119,COSM12479,COSM13296,COSM3092256,COSM753749	Cancer	Intestinal	PC
P17B-CATATGGAGTACTTGC-1	CDKN2A:9:21971017	*CDKN2A*	9	21971017	21971017	G	A	p.P114L	rs121913386,CM983988,COSM12476,COSM13830,COSM3092257,COSM4408164,COSM4408165,COSM4408166,COSM4408167,COSM4605168,COSM4605169,COSM4605170,COSM753742,COSM753743	Cancer	Intestinal	PC

P17B-CATATGGAGTACTTGC-1	CDKN2A:9:21971017	*CDKN2A*	9	21971017	21971018	GG	AA	p.P114F		Cancer	Intestinal	PC
P17B-CATCGGGCATTGGCGC-1	CDKN2A:9:21970971	*CDKN2A*	9	21970971	21970971	G	C	p.Y129*	COSM126614,COSM13221,COSM28562,COSM3788241	Cancer	Intestinal	PC
P17B-CATGCCTGTGTCCTCT-1	CCND1:11:69466022	*CCND1*	11	69466022	69466022	C	T	p.P287L	COSM2043470,COSM226265,COSM931397	Cancer	Intestinal	PMC
P17B-CCAGCGACACTCTGTC-1	CDKN2A:9:21971000	*CDKN2A*	9	21971000	21971000	C	T	p.E120K	CD972119,COSM12479,COSM13296,COSM3092256,COSM753749	Cancer	Intestinal	PC
P17B-CCA	KRAS:12:	*KR*	12	2538	253	G	T	p.Q61K	rs121913238,COSM1159597,COSM549,COSM550	Cancer	Int	PC

GCGACACTCTGTC-1	25380277	*AS*		0277	80277						estinal	
P17B-CCAGCGACACTCTGTC-1	KRAS:12:25378647	*KRAS*	12	25378647	25378647	T	G	p.K117N	rs770248150,COSM1256061,COSM1562192,COSM19940,COSM28519	Cancer	Intestinal	PC
P17B-CCAGCGACACTCTGTC-1	CDKN2A:9:21971035	*CDKN2A*	9	21971035	21971035	T	A	p.D108V	COSM1638187,COSM4767456,COSM4767457,COSM4767458,COSM4767459,COSM753739,COSM753740,COSM753741	Cancer	Intestinal	PC
P17B-CCAGCGACACTCTGTC-1	CCND1:11:69466021	*CCND1*	11	69466021	69466021	C	G	p.P287A	COSM4855094,COSM4855095,COSM931396	Cancer	Intestinal	PC
P17B-CCAGCGACACTC	KRAS:12:25380277	*KRAS*	12	25380277	253802	GA	TT	p.Q61K	COSM4387500,COSM87298	Cancer	Intesti	PC

TGTC-1					78						nal	
P17B-CCGGGATCACACTGCG-1	CCND1:11:69466021	*CCND1*	11	69466021	69466021	C	T	p.P287S	COSM4855094,COSM4855095,COSM931396	Cancer	Intestinal	PC
P17B-CCTCTGACACGGTAAG-1	PTEN:10:89725043	*PTEN*	10	89725043	89725043	G	-	p.X343_splice	COSM1180410,COSM5962,COSM921160	Cancer	Intestinal	PC
P17B-CCTCTGACACGGTAAG-1	CDKN2A:9:21970987	*CDKN2A*	9	21970987	21971685	CGATGGCCCAGCTCCTCAGCCAGGTCCACGGGCAGACGGCCCCAGGCATCGCGCACGTCCAGCCGCGCCCCGGCCCGGTGCAGCACCACCAGCGTGTCCAGGAAGCCCTCCCGGGCAGCGTCGTGCACGGGTCGGGTGAGAGTGGCGGGGTCGGCGCAGTTGGGCTCCGCGCCGTGGAGCAGCAGCAGCTCCGCCACTCGGGCGCTGCCCATCATCATGACCTGCCAGAGAGAACAGAATGGTCAGAGCCAGGGTGGGGGCCGGCATGACGGAAAGGAAGCTTGTGTAGAGCCCCCTCACCGCCAAGCAGACCCCCACACAAGCCCCAGGTGTCTAATTACCCCTACATTTGCTTCCAGTTTCCAATTTCCTTCTTGAGTTCTCTATCCATTCTTCAGTACACAATGAATTCCATTATATCCTCCGAACTTCTGCGGAGCTGTCGTCACAGGCAGAGAGCACTGTGAGGCACGGGCAAAATAGCAAAGGGGCAGGGACAGACTGACTTTTACTCCAGGCTAACTTCCTGTATTTCCCCTGAGATACAACTACTGAAATTTCTTCCTGAAATTATGTTAGGCCTGGAGATTTTTTTTTTTTTTTTTTGTTCACTGCTGTATATCCAAGCGCAGAATGTGGTAATTGTTAAAAAGAGAAA	-	p.X51_splice		Cancer	Intestinal	PC

						ACTTGTTTGTTTGTTAAAACAAATTCTCACAAAACTTTTAAG						
P17B-CCTCTGACACGGTAAG-1	CDKN2A:9:21970972	*CDKN2A*	9	21970972	21970972	T	C	p.Y129C	COSM13633	Cancer	Intestinal	PC
P17B-CGATTGAAGTTGTAGA-1	CDKN2A:9:21970969	*CDKN2A*	9	21970969	21970969	A	T	p.L130Q	COSM13670,COSM18438,COSM28675,COSM33799,COSM3395738,COSM3395739,COSM3788239,COSM3788240,COSM4571148	Cancer	Intestinal	PC
P17B-CGTGAGCGTGAGCGAT-1	CDKN2A:9:21971017	*CDKN2A*	9	21971017	21971017	G	T	p.P114H	CM983988,COSM12476,COSM13830,COSM3092257,COSM4408164,COSM4408165,COSM4408166,COSM4408167,COSM4605168,COSM4605169,COSM4605170,COSM753742,COSM753743	Cancer	Intestinal	PC
P17B-CGTGAGCGTGAGCGAT-1	CDKN2A:9:21971017	*CDKN2A*	9	21971017	21971018	GG	AA	p.P114F		Cancer	Intestinal	PC

P17B-CTAGCCTCAAGGACTG-1	CDKN2A:9:21971036	*CDKN2A*	9	21971036	21971036	C	G	p.D108H	CM071585,CM973278,COSM12484,COSM1314728,COSM13489,COSM13520,COSM1674414,COSM753735,COSM753736,COSM753737,COSM753738	Cancer	Intestinal	PC
P17B-CTAGCCTCAAGGACTG-1	CREBBP:16:3786707	*CREBBP*	16	3786707	3786707	A	T	p.W1502R	COSM5363732	Cancer	Intestinal	PC
P17B-CTAGCCTTCCCACTTG-1	SMARCA4:19:11106926	*SMARCA4*	19	11106926	11106928	AGA	-	p.K546del	COSM5576272,COSM5576273	Cancer	Intestinal	PC
P17B-CTTGGCTAGGTGGGTT-1	CDKN2A:9:21970971	*CDKN2A*	9	21970971	21970971	G	T	p.Y129*	COSM126614,COSM13221,COSM28562,COSM3788241	Cancer	Intestinal	PC
P17B-CTTT	CDKN2A:	*CDK*	9	2197	219	G	T	p.Y1	COSM126614,COSM13221,COSM28562,COSM3788241	Cancer	Int	PC

GCGTCTCGAGTA-1	9:21970971	*N2A*		0971	70971			29*			estinal	
P17B-GAACATCCATCAGTAC-1	CDKN2A:9:21970971	*CDKN2A*	9	21970971	21970971	G	C	p.Y129*	COSM126614,COSM13221,COSM28562,COSM3788241	Cancer	Intestinal	PC
P17B-GAACATCCATCAGTAC-1	CDKN2A:9:21971035	*CDKN2A*	9	21971035	21971035	T	A	p.D108V	COSM1638187,COSM4767456,COSM4767457,COSM4767458,COSM4767459,COSM753739,COSM753740,COSM753741	Cancer	Intestinal	PC
P17B-GAACATCCATCAGTAC-1	CDKN2A:9:21971000	*CDKN2A*	9	21971000	21971000	C	T	p.E120K	CD972119,COSM12479,COSM13296,COSM3092256,COSM753749	Cancer	Intestinal	PC
P17B-GAACATCCATCA	SMARCA4:19:1110	*SMARC*	19	11106926	111069	AGA	-	p.K546del	COSM5576272,COSM5576273	Cancer	Intesti	PC

GTAC-1	6926	*A4*			28						nal	
P17B-GCAAACTAGAAGATTC-1	CDKN2A:9:21971000	*CDKN2A*	9	21971000	21971000	C	A	p.E120*	CD972119,COSM12479,COSM13296,COSM3092256,COSM753749	Cancer	Intestinal	Tumor
P17B-GCAATCAAGACTAAGT-1	CDKN2A:9:21971000	*CDKN2A*	9	21971000	21971000	C	T	p.E120K	CD972119,COSM12479,COSM13296,COSM3092256,COSM753749	Cancer	Intestinal	PC
P17B-GCACATAGTTAGGGTG-1	CDKN2A:9:21970971	*CDKN2A*	9	21970971	21970971	G	T	p.Y129*	COSM126614,COSM13221,COSM28562,COSM3788241	Cancer	Intestinal	PC
P17B-GCAGTTACATTCCTGC-1	CDKN2A:9:21971029	*CDKN2A*	9	21971029	21971029	C	T	p.W110*	rs1057519852,COSM12481,COSM126617,COSM126618,COSM3382498	Cancer	Intestinal	PC

P17B-GCATACAAGCCCTAAT-1	CDKN2A:9:21970969	*CDKN2A*	9	21970969	21970969	A	T	p.L130Q	COSM13670,COSM18438,COSM28675,COSM33799,COSM3395738,COSM3395739,COSM3788239,COSM3788240,COSM4571148	Cancer	Intestinal	PC
P17B-GGACAGACATGTAAGA-1	CDKN2A:9:21971110	*CDKN2A*	9	21971110	21971110	T	C	p.H83R	rs1057519881,COSM1167960,COSM1167961,COSM12494,COSM13253,COSM3656622,COSM5410930,COSM5410931,COSM5410932,COSM5410933,COSM5822047,COSM5822048,COSM5822049	Cancer	Intestinal	PC
P17B-GGCGACTAGCGTGAGT-1	CDKN2A:9:21971035	*CDKN2A*	9	21971035	21971035	T	A	p.D108V	COSM1638187,COSM4767456,COSM4767457,COSM4767458,COSM4767459,COSM753739,COSM753740,COSM753741	Cancer	Intestinal	PC
P17B-GGCTGGTAGTGTACGG-1	CDKN2A:9:21970971	*CDKN2A*	9	21970971	21970971	G	C	p.Y129*	COSM126614,COSM13221,COSM28562,COSM3788241	Cancer	Intestinal	PC
P17B-GGT	CDKN2A:	*CDK*	9	2197	219	A	C	p.L1	COSM13670,COSM18438,COSM28675,COSM33799,COSM3395738,	Cancer	Int	PC

GCGTTCCCAAGTA-1	9:21970969	*N2A*		0969	70969			30R	COSM3395739,COSM3788239,COSM3788240,COSM4571148		estinal	
P17B-GGTGTTAAGTGCTGCC-1	CDKN2A:9:21970971	*CDKN2A*	9	21970971	21970971	G	T	p.Y129*	COSM126614,COSM13221,COSM28562,COSM3788241	Cancer	Intestinal	PC
P17B-GGTGTTAAGTGCTGCC-1	CDKN2A:9:21970969	*CDKN2A*	9	21970969	21970969	A	T	p.L130Q	COSM13670,COSM18438,COSM28675,COSM33799,COSM3395738,COSM3395739,COSM3788239,COSM3788240,COSM4571148	Cancer	Intestinal	PC
P17B-GGTGTTAAGTGCTGCC-1	CDKN2A:9:21970970	*CDKN2A*	9	21970970	21970970	G	C	p.L130V	CM080134,COSM3395740,COSM3395741,COSM3395742	Cancer	Intestinal	PC
P17B-GTGCAGCCAGAT	CDKN2A:9:2197	*CDKN2A*	9	21971029	219710	C	T	p.W110*	rs1057519852,COSM12481,COSM126617,COSM126618,COSM3382498	Cancer	Intesti	PC

GGCA-1	1029				29						nal	
P17B-GTGCAGCCAGATGGCA-1	CDKN2A:9:21971017	*CDKN2A*	9	21971017	21971017	G	A	p.P114L	rs121913386,CM983988,COSM12476,COSM13830,COSM3092257,COSM4408164,COSM4408165,COSM4408166,COSM4408167,COSM4605168,COSM4605169,COSM4605170,COSM753742,COSM753743	Cancer	Intestinal	PC
P17B-GTGCAGCCAGATGGCA-1	CDKN2A:9:21970972	*CDKN2A*	9	21970972	21970972	T	C	p.Y129C	COSM13633	Cancer	Intestinal	PC
P17B-GTGCAGCCAGATGGCA-1	CDKN2A:9:21971017	*CDKN2A*	9	21971017	21971018	GG	AA	p.P114F		Cancer	Intestinal	PC
P17B-GTGCAGCCAGATGGCA-1	CDKN2A:9:21971155	*CDKN2A*	9	21971155	21971694	GCGCCGTGGAGCAGCAGCAGCTCCGCCACTCGGGCGCTGCCCATCATCATGACCTGCCAGAGAGAACAGAATGGTCAGAGCCAGGGTGGGGGCCGGCATGACGGAAAGGAAGCTTGTGTAGAGCCCCCTCACCGCCAAGCAGACCCCCACACAAGCCCCAGGTGTCTAATTACCCCTACATTTGCTTCCAGTTTCCAATTTCCTTCTTGAGTTCTCTATCCATTCTTCAGTACACAATGAATTCCATTATATCCTCCGAACTTCTGCGGAGCTGTCGTCACAGGCAGAGAGCACTGTGAGGCACGGGCAAAATAGCAAAGGGGCAGGGACAGACTGACTTTTACTCCA	-	p.X51_splice		Cancer	Intestinal	PC

						GGCTAACTTCCTGTATTTCCCCTGAGATACAACTACTGAAATTTCTTCCTGAAATTATGTTAGGCCTGGAGATTTTTTTTTTTTTTTTTGTTCACTGCTGTATATCCAAGCGCAGAATGTGGTAATTGTTAAAAAGAGAAAACTTGTTTGTTTGTTAAAACAAATTCTCACAAAACTTTTAAGTTACACTTA						
P17B-GTGCAGCTCTGCGACG-1	CDKN2A:9:21971017	*CDKN2A*	9	21971017	21971018	GG	AA	p.P114F		Cancer	Intestinal	PC
P17B-GTGCAGCTCTGCGACG-1	CDKN2A:9:21971017	*CDKN2A*	9	21971017	21971017	G	A	p.P114L	rs121913386,CM983988,COSM12476,COSM13830,COSM3092257,COSM4408164,COSM4408165,COSM4408166,COSM4408167,COSM4605168,COSM4605169,COSM4605170,COSM753742,COSM753743	Cancer	Intestinal	PC
P17B-GTGCATAGTCAGGACA-1	CDKN2A:9:21971016	*CDKN2A*	9	21971016	21971016	G	A	p.R170C	COSM4387396,COSM4993334,COSM4993335,COSM4993336,COSM4993337,COSM85158	Cancer	Intestinal	PC
P17B-GTGCATAGTCAG	CDKN2A:9:21971016	*CDKN2A*	9	21971016	219710	GG	AA	p.P114L	COSM255155,COSM28573	Cancer	Intestin	PC

GACA-1					17						al	
P17B-GTTAAGCCACGGCGTT-1	CDKN2A:9:21970987	*CDKN2A*	9	21970987	21971685	CGATGGCCCAGCTCCTCAGCCAGGTCCACGGGCAGACGGCCCCAGGCATCGCGCACGTCCAGCCGCGCCCCGGCCCGGTGCAGCACCACCAGCGTGTCCAGGAAGCCCTCCCGGGCAGCGTCGTGCACGGGTCGGGTGAGAGTGGCGGGGTCGGCGCAGTTGGGCTCCGCGCCGTGGAGCAGCAGCAGCTCCGCCACTCGGGCGCTGCCCATCATCATGACCTGCCAGAGAGAACAGAATGGTCAGAGCCAGGGTGGGGGCCGGCATGACGGAAAGGAAGCTTGTGTAGAGCCCCCTCACCGCCAAGCAGACCCCCACACAAGCCCCAGGTGTCTAATTACCCCTACATTTGCTTCCAGTTTCCAATTTCCTTCTTGAGTTCTCTATCCATTCTTCAGTACACAATGAATTCCATTATATCCTCCGAACTTCTGCGGAGCTGTCGTCACAGGCAGAGAGCACTGTGAGGCACGGGCAAAATAGCAAAGGGGCAGGGACAGACTGACTTTTACTCCAGGCTAACTTCCTGTATTTCCCCTGAGATACAACTACTGAAATTTCTTCCTGAAATTATGTTAGGCCTGGAGATTTTTTTTTTTTTTTTGTTCACTGCTGTATATCCAAGCGCAGAATGTGGTAATTGTTAAAAAGAGAAAACTTGTTTGTTTGTTAAAACAAATTCTCACAAAACTTTTAAG	-	p.X51_splice		Cancer	Intestinal	PC
P17B-GTTAAGCCACGGCGTT-1	CDKN2A:9:21970969	*CDKN2A*	9	21970969	21970969	A	T	p.L130Q	COSM13670,COSM18438,COSM28675,COSM33799,COSM3395738,COSM3395739,COSM3788239,COSM3788240,COSM4571148	Cancer	Intestinal	PC
P17B-TACTCATGTGTGCCTG-1	STAG2:X:123179198	*STAG2*	X	123179198	123179198	G	A	p.R216Q	COSM1138066,COSM487901	Cancer	Intestinal	PC

P17B-TATCTCAGTCCATCCT-1	CCND1:11:69466021	*CCND1*	11	69466021	69466021	C	A	p.P287T	COSM4855094,COSM4855095,COSM931396	Cancer	Intestinal	PC
P17B-TATGCCCAGACTGTAA-1	CDKN2A:9:21971029	*CDKN2A*	9	21971029	21971029	C	T	p.W110*	rs1057519852,COSM12481,COSM126617,COSM126618,COSM3382498	Cancer	Intestinal	PC
P17B-TATGCCCAGACTGTAA-1	CDKN2A:9:21971018	*CDKN2A*	9	21971018	21971018	G	T	p.P114T	CM014526,CX073790,COSM13713,COSM3952629,COSM3952630,COSM3952631,COSM3952632	Cancer	Intestinal	PC
P17B-TATGCCCAGACTGTAA-1	CDKN2A:9:21971036	*CDKN2A*	9	21971036	21971036	C	G	p.D108H	CM071585,CM973278,COSM12484,COSM1314728,COSM13489,COSM13520,COSM1674414,COSM753735,COSM753736,COSM753737,COSM753738	Cancer	Intestinal	PC
P17B-TAT	CDKN2A:	*CDK*	9	2197	219	G	T	p.Y1	COSM126614,COSM13221,COSM28562,COSM3788241	Cancer	Int	PC

GCCCAGACTGTAA-1	9:21970971	*N2A*		0971	70971			29*			estinal	
P17B-TATGCCCAGACTGTAA-1	CDKN2A:9:21970969	*CDKN2A*	9	21970969	21970969	A	T	p.L130Q	COSM13670,COSM18438,COSM28675,COSM33799,COSM3395738,COSM3395739,COSM3788239,COSM3788240,COSM4571148	Cancer	Intestinal	PC
P17B-TCAACGAAGACTTTCG-1	CDKN2A:9:21970971	*CDKN2A*	9	21970971	21970971	G	T	p.Y129*	COSM126614,COSM13221,COSM28562,COSM3788241	Cancer	Intestinal	PC
P17B-TCAACGAAGACTTTCG-1	CDKN2A:9:21970969	*CDKN2A*	9	21970969	21970969	A	C	p.L130R	COSM13670,COSM18438,COSM28675,COSM33799,COSM3395738,COSM3395739,COSM3788239,COSM3788240,COSM4571148	Cancer	Intestinal	PC
P17B-TCAGGTACACAA	CCND1:11:6946	*CCND1*	11	69466021	6946660	C	G	p.P287A	COSM4855094,COSM4855095,COSM931396	Cancer	Intesti	PC

CGTT-1	6021				21						nal	
P17B-TCAGGTACACAACGTT-1	CDKN2A:9:21970987	*CDKN2A*	9	21970987	21971685	CGATGGCCCAGCTCCTCAGCCAGGTCCACGGGCAGACGGCCCCAGGCATCGCGCACGTCCAGCCGCGCCCCGGCCCGGTGCAGCACCACCAGCGTGTCCAGGAAGCCCTCCCGGGCAGCGTCGTGCACGGGTCGGGTGAGAGTGGCGGGGTCGGCGCAGTTGGGCTCCGCGCCGTGGAGCAGCAGCAGCTCCGCCACTCGGGCGCTGCCCATCATCATGACCTGCCAGAGAGAACAGAATGGTCAGAGCCAGGGTGGGGGCCGGCATGACGGAAAGGAAGCTTGTGTAGAGCCCCCTCACCGCCAAGCAGACCCCCACACAAGCCCCAGGTGTCTAATTACCCCTACATTTGCTTCCAGTTTCCAATTTCCTTCTTGAGTTCTCTATCCATTCTTCAGTACACAATGAATTCCATTATATCCTCCGAACTTCTGCGGAGCTGTCGTCACAGGCAGAGAGCACTGTGAGGCACGGGCAAAATAGCAAAGGGGCAGGGACAGACTGACTTTTACTCCAGGCTAACTTCCTGTATTTCCCCTGAGATACAACTACTGAAATTTCTTCCTGAAATTATGTTAGGCCTGGAGATTTTTTTTTTTTTTTTTGTTCACTGCTGTATATCCAAGCGCAGAATGTGGTAATTGTTAAAAAGAGAAAACTTGTTTGTTTGTTAAAACAAATTCTCACAAAACTTTTAAG	-	p.X51_splice		Cancer	Intestinal	PC
P17B-TCGAGGCCACTCTGTC-1	RAC1:7:6441974	*RAC1*	7	6441974	6441974	C	T	p.A178V	COSM1154840,COSM389868	Cancer	Intestinal	PC
P17B-TCGAGGCGTACGCACC-1	CDK4:12:58145430	*CDK4*	12	58145430	58145430	C	A	p.R24L	CM980320,COSM1989836	Cancer	Intestin	PC

											al	
P17B-TCGAGGCGTACGCACC-1	SMARCB1:22:24176339	*SMARCB1*	22	24176339	24176339	G	A	p.R377H	rs387906812,CM122478,COSM1578803,COSM27977,COSM4596765,COSM4596766,COSM989	Cancer	Intestinal	PC
P17B-TCGCGTTCACCATCCT-1	CDKN2A:9:21970987	*CDKN2A*	9	21970987	21971685	CGATGGCCCAGCTCCTCAGCCAGGTCCACGGGCAGACGGCCCCAGGCATCGCGCACGTCCAGCCGCGCCCCGGCCCGGTGCAGCACCACCAGCGTGTCCAGGAAGCCCTCCCGGGCAGCGTCGTGCACGGGTCGGGTGAGAGTGGCGGGGTCGGCGCAGTTGGGCTCCGCGCCGTGGAGCAGCAGCAGCTCCGCCACTCGGGCGCTGCCCATCATCATGACCTGCCAGAGAGAACAGAATGGTCAGAGCCAGGGTGGGGGCCGGCATGACGGAAAGGAAGCTTGTGTAGAGCCCCCTCACCGCCAAGCAGACCCCCACACAAGCCCCAGGTGTCTAATTACCCCTACATTTGCTTCCAGTTTCCAATTTCCTTCTTGAGTTCTCTATCCATTCTTCAGTACACAATGAATTCCATTATATCCTCCGAACTTCTGCGGAGCTGTCGTCACAGGCAGAGAGCACTGTGAGGCACGGGCAAAATAGCAAAGGGGCAGGGACAGACTGACTTTTACTCCAGGCTAACTTCCTGTATTTCCCCTGAGATACAACTACTGAAATTTCTTCCTGAAATTATGTTAGGCCTGGAGATTTTTTTTTTTTTTTTGTTCACTGCTGTATATCCAAGCGCAGAATGTGGTAATTGTTAAAAAGAGAAAACTTGTTTGTTTGTTAAAACAAATTCTCACAAAACTTTTAAG	-	p.X51_splice		Cancer	Intestinal	PC
P17B-TGGGCGTGTGACGGTA-1	CDKN2A:9:21970969	*CDKN2A*	9	21970969	21970969	A	C	p.L130R	COSM13670,COSM18438,COSM28675,COSM33799,COSM3395738,COSM3395739,COSM3788239,COSM3788240,COSM4571148	Cancer	Intestinal	PC

P17B-TGGGCGTGTGACGGTA-1	SMARCB1:22:24176339	*SMARCB1*	22	24176339	24176339	G	A	p.R377H	rs387906812,CM122478,COSM1578803,COSM27977,COSM4596765,COSM4596766,COSM989	Cancer	Intestinal	PC
P17B-TTCGAAGCAAACAACA-1	CCND1:11:69466022	*CCND1*	11	69466022	69466022	C	T	p.P287L	COSM2043470,COSM226265,COSM931397	Cancer	Intestinal	PMC
P17B-TTTCCTCTCGTTACGA-1	CDKN2A:9:21971029	*CDKN2A*	9	21971029	21971029	C	T	p.W110*	rs1057519852,COSM12481,COSM126617,COSM126618,COSM3382498	Cancer	Intestinal	PC
P17B-TTTGTCAAGCCTTGAT-1	CDKN2A:9:21971028	*CDKN2A*	9	21971028	21971028	C	T	p.W110*	rs121913389,CM060208,COSM12547,COSM126615,COSM126616,COSM1598222,COSM48297	Cancer	Intestinal	PC
P18A-AAC	CCND1:1	*CCN*	11	6946	694	C	T	p.T2	COSM931395	Adjacent	Int	GMC

TCC CTC TAT CGC C-1	1:6 946 601 9	*D 1*		60 19	6 6 0 1 9			86 I		no n-can cer	e s ti n a l	
P18A - AGA GCG ACA AGC TGA G-1	CC ND 1:1 1:6 946 602 1	*C C N D 1*	1 1	69 46 60 21	6 9 4 6 6 0 2 1	C	A	p. P2 87 T	COSM4855094,COSM4855095,COSM931396	Ad jac ent no n-can cer	I n t e s ti n a l	G M C
P18A - AGG CCG TGT ACA TCC A-1	RA C1: 7:6 441 974	*R A C 1*	7	64 41 97 4	6 4 4 1 9 7 4	C	T	p. A1 78 V	COSM1154840,COSM389868	Ad jac ent no n-can cer	I n t e s ti n a l	M S C
P18A - CAG CAG CTC CTT GGT C-1	PTP N11 :12: 112 888 199	*P T P N 11*	1 2	11 28 88 19 9	1 1 2 8 8 8 1 9 9	C	T	p. A7 2V	rs121918454,CM013417,COSM13015,COSM13035,COSM5945277	Ad jac ent no n-can cer	I n t e s ti n a l	G M C
P18A - CAT CGA ACA TAT	CC ND 1:1 1:6 946	*C C N D 1*	1 1	69 46 60 22	6 9 4 6 6 0	C	G	p. P2 87 R	COSM2043470,COSM226265,COSM931397	Ad jac ent no n-	I n t e s ti	P M C

ACCG-1	6022				22					cancer	nal	
P18A-GATCGATAGTCTCGGC-1	CCND1:11:69466022	*CCND1*	11	69466022	69466022	C	G	p.P287R	COSM2043470,COSM226265,COSM931397	Adjacent non-cancer	Intestinal	PC
P18A-TCAGCAACAATGTTGC-1	KRAS:12:25380279	*KRAS*	12	25380279	25380279	C	A	p.G60V	rs727503108,COSM1667041,COSM4531523,COSM548,COSM5879374,COSM87290	Adjacent non-cancer	Intestinal	MSC
P18A-TGTATTCGTAACGTTC-1	CCND1:11:69466022	*CCND1*	11	69466022	69466022	C	G	p.P287R	COSM2043470,COSM226265,COSM931397	Adjacent non-cancer	Intestinal	PMC
P18B-AAACGGGAGTTAGCGG-1	CCND1:11:69466018	*CCND1*	11	69466018	69466018	A	G	p.T286A		Cancer	Intestinal	PMC

P18B-AACTCAGTCGGCTACG-1	CCND1:11:69466022	*CCND1*	11	69466022	69466022	C	G	p.P287R	COSM2043470,COSM226265,COSM931397	Cancer	Intestinal	Chief
P18B-CACACAACACGACGAA-1	CCND1:11:69466021	*CCND1*	11	69466021	69466021	C	A	p.P287T	COSM4855094,COSM4855095,COSM931396	Cancer	Intestinal	PMC
P18B-CGGAGCTAGTAACCCT-1	KRAS:12:25378648	*KRAS*	12	25378648	25378648	T	C	p.K117R	COSM4696721,COSM4696722	Cancer	Intestinal	Enteroendocrine
P18B-CTCGAAACACTACAGT-1	CCND1:11:69466022	*CCND1*	11	69466022	69466022	C	G	p.P287R	COSM2043470,COSM226265,COSM931397	Cancer	Intestinal	GMC
P18B-TCA	EP300:22:	*EP*	22	4156	415	G	A	p.W14	COSM1205369	Cancer	Int	GMC

TTTGCACTTCTGC-1	41566520	*300*		6520	66520			66*			estinal	
P19A-ATAACGCTCGTCTGAA-1	RAC1:7:6441974	*RAC1*	7	6441974	6441974	C	T	p.A178V	COSM1154840,COSM389868	Adjacent non-cancer	Intestinal	GMC
P19A-CATTCGCTCTCGTTTA-1	FBXW7:4:153244091	*FBXW7*	4	153244091	153244091	C	T	p.R689Q	COSM1594355,COSM302214,COSM302215,COSM302216,COSM302217	Adjacent non-cancer	Intestinal	Enteroendocrine
P19A-CGGACGTCACTTCGGA-1	CCND1:11:69466022	*CCND1*	11	69466022	69466022	C	T	p.P287L	COSM2043470,COSM226265,COSM931397	Adjacent non-cancer	Intestinal	GMC
P19B-AACTCAGCAGCT	CDKN2A:9:2197	*CDKN2A*	9	21971018	219710	G	A	p.P114S	rs104894104,CM014526,CX073790,COSM13713,COSM3952629,COSM3952630,COSM3952631,COSM3952632	Cancer	Intesti	MSC

GCAC-1	1018				18						nal	
P19B-AACTCAGCAGCTGCAC-1	RAC1:7:6431628	*RAC1*	7	6431628	6431628	C	A	p.Q61K		Cancer	Intestinal	MSC
P19B-AACTCCCAGTGGGCTA-1	CCND1:11:69466022	*CCND1*	11	69466022	69466022	C	G	p.P287R	COSM2043470,COSM226265,COSM931397	Cancer	Intestinal	MSC
P19B-ACACCGGTCCAGAAGG-1	CDKN2A:9:21971017	*CDKN2A*	9	21971017	21971017	G	T	p.P114H	CM983988,COSM12476,COSM13830,COSM3092257,COSM4408164,COSM4408165,COSM4408166,COSM4408167,COSM4605168,COSM4605169,COSM4605170,COSM753742,COSM753743	Cancer	Intestinal	MSC
P19B-ACACCGGTCCAGAAGG-1	CDKN2A:9:21971017	*CDKN2A*	9	21971017	21971018	GG	AA	p.P114F		Cancer	Intestinal	MSC

P19B-ACACCGGTCCAGAAGG-1	CDKN2A:9:21971018	*CDKN2A*	9	21971018	21971018	G	T	p.P114T	CM014526,CX073790,COSM13713,COSM3952629,COSM3952630,COSM3952631,COSM3952632	Cancer	Intestinal	MSC
P19B-ACCCACTAGAGAGCTC-1	RAC1:7:6431628	*RAC1*	7	6431628	6431628	C	A	p.Q61K		Cancer	Intestinal	PC
P19B-ACCGTAACATGCCCGA-1	RAC1:7:6441974	*RAC1*	7	6441974	6441974	C	T	p.A178V	COSM1154840,COSM389868	Cancer	Intestinal	Tumor
P19B-ACCGTAACATGCCCGA-1	CCND1:11:69466022	*CCND1*	11	69466022	69466022	C	G	p.P287R	COSM2043470,COSM226265,COSM931397	Cancer	Intestinal	Tumor
P19B-AGA	CCND1:1	*CCN*	11	6946	694	C	G	p.P2	COSM4855094,COSM4855095,COSM931396	Cancer	Int	MSC

TCTGTCAGAAATG-1	1:69466021	*D1*		6021	66021			87A			estinal	
P19B-AGCCTAATCGTCACGG-1	RAC1:7:6426907	*RAC1*	7	6426907	6426907	C	T	p.P34S	COSM3640041,COSM3640042	Cancer	Intestinal	PC
P19B-AGGGATGTCAACCATG-1	CCND1:11:69466022	*CCND1*	11	69466022	69466022	C	T	p.P287L	COSM2043470,COSM226265,COSM931397	Cancer	Intestinal	MSC
P19B-AGGTCATAGATTACC C-1	CCND1:11:69466022	*CCND1*	11	69466022	69466022	C	G	p.P287R	COSM2043470,COSM226265,COSM931397	Cancer	Intestinal	Chief
P19B-AGTGAGGAGTAC	CDKN2A:9:2197	*CDKN2A*	9	21970971	219709	G	C	p.Y129*	COSM126614,COSM13221,COSM28562,COSM3788241	Cancer	Intesti	Tumor

CGGA-1	0971				71						nal	
P19B-ATTACTCCAGGAACGT-1	RAC1:7:6441974	*RAC1*	7	6441974	6441974	C	T	p.A178V	COSM1154840,COSM389868	Cancer	Intestinal	GMC
P19B-CAGCATATCGCGGATC-1	CDKN2A:9:21970971	*CDKN2A*	9	21970971	21970971	G	T	p.Y129*	COSM126614,COSM13221,COSM28562,COSM3788241	Cancer	Intestinal	MSC
P19B-CATCAAGGTAAGAGGA-1	CDKN2A:9:21970969	*CDKN2A*	9	21970969	21970969	A	C	p.L130R	COSM13670,COSM18438,COSM28675,COSM33799,COSM3395738,COSM3395739,COSM3788239,COSM3788240,COSM4571148	Cancer	Intestinal	PC
P19B-CGGACGTTCCAAATGC-1	RAC1:7:6439806	*RAC1*	7	6439806	6439806	A	G	p.N111S	COSM1684687,COSM5624655,COSM5624656	Cancer	Intestinal	MSC

P19B-CGTCTACAGACACTAA-1	RAC1:7:6441974	*RAC1*	7	6441974	6441974	C	T	p.A178V	COSM1154840,COSM389868	Cancer	Intestinal	MSC
P19B-CGTTCTGTCCTCGCAT-1	CDKN2A:9:21970972	*CDKN2A*	9	21970972	21970972	T	C	p.Y129C	COSM13633	Cancer	Intestinal	MSC
P19B-CTAATGGCAGATCCAT-1	CDKN2A:9:21968242	*CDKN2A*	9	21968242	21968242	C	T	p.X153_splice	CS127044,COSM21562,COSM3952628,COSM99937	Cancer	Intestinal	MSC
P19B-CTAATGGCAGATCCAT-1	CDKN2A:9:21968242	*CDKN2A*	9	21968242	21968243	-	A	p.X153_splice		Cancer	Intestinal	MSC
P19B-CTA	RAC1:7:6	*RA*	7	6441	644	C	T	p.A1	COSM1154840,COSM389868	Cancer	Int	EC

GAGTGTGGCTCCA-1	441974	*C1*		974	1974			78V			estinal	
P19B-CTCCTAGCATCCCACT-1	CCND1:11:69466022	*CCND1*	11	69466022	69466022	C	G	p.P287R	COSM2043470,COSM226265,COSM931397	Cancer	Intestinal	MSC
P19B-CTCGGAGCAAGAGGCT-1	CDKN2A:9:21970987	*CDKN2A*	9	21970987	21971685	CGATGGCCCAGCTCCTCAGCCAGGTCCACGGGCAGACGGCCCCAGGCATCGCGCACGTCCAGCCGCGCCCCGGCCCGGTGCAGCACCACCAGCGTGTCCAGGAAGCCCTCCCGGGCAGCGTCGTGCACGGGTCGGGTGAGAGTGGCGGGGTCGGCGCAGTTGGGCTCCGCGCCGTGGAGCAGCAGCAGCTCCGCCACTCGGGCGCTGCCCATCATCATGACCTGCCAGAGAGAACAGAATGGTCAGAGCCAGGGTGGGGGCCGGCATGACGGAAAGGAAGCTTGTGTAGAGCCCCCTCACCGCCAAGCAGACCCCCACACAAGCCCCAGGTGTCTAATTACCCCTACATTTGCTTCCAGTTTCCAATTTCCTTCTTGAGTTCTCTATCCATTCTTCAGTACACAATGAATTCCATTATATCCTCCGAACTTCTGCGGAGCTGTCGTCACAGGCAGAGAGCACTGTGAGGCACGGGCAAAATAGCAAAGGGGCAGGGACAGACTGACTTTTACTCCAGGCTAACTTCCTGTATTTCCCCTGAGATACAACTACTGAAATTTCTTCCTGAAATTATGTTAGGCCTGGAGATTTTTTTTTTTTTTTTTGTTCACTGCTGTATATCCAAGCGCAGAATGTGGTAATTGTTAAAAAGAGAAAACTTGTTTGTTTGTTAAAACAAATTCTCACAAAACTTTTAAG	-	p.X51_splice		Cancer	Intestinal	MSC
P19B-CTCTAATGT	CCND1:11:6946	*CCND1*	11	69466022	69466	C	G	p.P287R	COSM2043470,COSM226265,COSM931397	Cancer	Intes	MSC

CTAGAGG-1	6022				022					tinal	
P19B-CTCTAATGTCTAGAGG-1	RAC1:7:6441974	*RAC1*	7	6441974	6441974	C	T	p.A178V	COSM1154840,COSM389868	Cancer	Intestinal	MSC
P19B-CTGATAGGTTTGTTGG-1	CDKN2A:9:21970987	*CDKN2A*	9	21970987	21971685	CGATGGCCCAGCTCCTCAGCCAGGTCCACGGGCAGACGGCCCCAGGCATCGCGCACGTCCAGCCGCGCCCCGGCCCGGTGCAGCACCACCAGCGTGTCCAGGAAGCCCTCCCGGGCAGCGTCGTGCACGGGTCGGGTGAGAGTGGCGGGGTCGGCGCAGTTGGGCTCCGCGCCGTGGAGCAGCAGCAGCTCCGCCACTCGGGCGCTGCCCATCATCATGACCTGCCAGAGAGAACAGAATGGTCAGAGCCAGGGTGGGGGCCGGCATGACGGAAAGGAAGCTTGTGTAGAGCCCCCTCACCGCCAAGCAGACCCCCACACAAGCCCCAGGTGTCTAATTACCCCTACATTTGCTTCCAGTTTCCAATTTCCTTCTTGAGTTCTCTATCCATTCTTCAGTACACAATGAATTCCATTATATCCTCCGAACTTCTGCGGAGCTGTCGTCACAGGCAGAGAGCACTGTGAGGCACGGGCAAAATAGCAAAGGGGCAGGGACAGACTGACTTTTACTCCAGGCTAACTTCCTGTATTTCCCCTGAGATACAACTACTGAAATTTCTTCCTGAAATTATGTTAGGCCTGGAGATTTTTTTTTTTTTTTTTGTTCACTGCTGTATATCCAAGCGCAGAATGTGGTAATTGTTAAAAAGAGAAAACTTGTTTGTTTGTTAAAACAAATTCTCACAAAACTTTTAAG	-	p.X51_splice		Cancer	Intestinal	PC
P19B-CTGGTCTCATCG	RAC1:7:6439806	*RAC1*	7	6439806	6439806	A	G	p.N111S	COSM1684687,COSM5624655,COSM5624656	Cancer	Intestin	MSC

ATGT-1											al	
P19B-GAAACTCAGATGTGGC-1	CCND1:11:69466021	*CCND1*	11	69466021	69466021	C	A	p.P287T	COSM4855094,COSM4855095,COSM931396	Cancer	Intestinal	MSC
P19B-GAAACTCAGATGTGGC-1	CCND1:11:69466022	*CCND1*	11	69466022	69466022	C	T	p.P287L	COSM2043470,COSM226265,COSM931397	Cancer	Intestinal	MSC
P19B-GAATGAATCGCAAACT-1	RAC1:7:6431629	*RAC1*	7	6431629	6431629	A	G	p.Q61R	COSM1131540	Cancer	Intestinal	MSC
P19B-GGCAATTGTAAAGTCA-1	CCND1:11:69466018	*CCND1*	11	69466018	69466018	A	G	p.T286A		Cancer	Intestinal	PC

P19B-GGCCGATGTCAGAGGT-1	CCND1:11:69466022	*CCND1*	11	69466022	69466022	C	T	p.P287L	COSM2043470,COSM226265,COSM931397	Cancer	Intestinal	Tumor
P19B-GTACTTTGTCTTTCAT-1	RAC1:7:6439807	*RAC1*	7	6439807	6439807	T	G	p.N111K	COSM3640063,COSM3640064,COSM5038555,COSM5038556	Cancer	Intestinal	MSC
P19B-GTTCTCGAGCGGCTTC-1	CCND1:11:69466022	*CCND1*	11	69466022	69466022	C	G	p.P287R	COSM2043470,COSM226265,COSM931397	Cancer	Intestinal	MSC
P19B-TAGGCATGTAGGACAC-1	CCND1:11:69466022	*CCND1*	11	69466022	69466022	C	G	p.P287R	COSM2043470,COSM226265,COSM931397	Cancer	Intestinal	MSC
P19B-TCG	RAC1:7:6	*RA*	7	6441	644	C	T	p.A1	COSM1154840,COSM389868	Cancer	Int	MSC

CGA GAG TAC GCG A-1	441 974	*C 1*		97 4	1 9 7 4			78 V			estinal	
P19B - TGA GAG GCA TTCT TAC- 1	RA C1: 7:6 441 974	*R A C 1*	7	64 41 97 4	6 4 4 1 9 7 4	C	T	p. A1 78 V	COSM1154840,COSM389868	Ca nce r	Intestinal	M S C
P19B - TGA GAG GCA TTCT TAC- 1	CC ND 1:1 1:6 946 602 2	*C C N D 1*	1 1	69 46 60 22	6 9 4 6 6 0 2 2	C	T	p. P2 87 L	COSM2043470,COSM226265,COS M931397	Ca nce r	Intestinal	M S C
P19B - TGC CCA TCA GGA TTG G-1	RA C1: 7:6 439 806	*R A C 1*	7	64 39 80 6	6 4 3 9 8 0 6	A	T	p. N1 11 I	COSM1684687,COSM5624655,CO SM5624656	Ca nce r	Intestinal	M S C
P19B - TGG CCA GTC GTC	CC ND 1:1 1:6 946	*C C N D 1*	1 1	69 46 60 22	6 9 4 6 6 0	C	G	p. P2 87 R	COSM2043470,COSM226265,COS M931397	Ca nce r	Intesti	M S C

TGCT-1	6022				22						nal	
P20A-ATTACTCAGGGTATCG-1	RAC1:7:6441974	*RAC1*	7	6441974	6441974	C	T	p.A178V	COSM1154840,COSM389868	Adjacent non-cancer	Diffuse	Chief
P20A-CAAGATCCACACAGAG-1	RAC1:7:6441974	*RAC1*	7	6441974	6441974	C	T	p.A178V	COSM1154840,COSM389868	Adjacent non-cancer	Diffuse	GMC
P20A-GATCTAGTCGAGCCCA-1	CCND1:11:69466022	*CCND1*	11	69466022	69466022	C	G	p.P287R	COSM2043470,COSM226265,COSM931397	Adjacent non-cancer	Diffuse	GMC
P20A-TAGACCATCGACCAGC-1	RAC1:7:6441974	*RAC1*	7	6441974	6441974	C	T	p.A178V	COSM1154840,COSM389868	Adjacent non-cancer	Diffuse	EC
P21A-AAA	CCND1:1	*CCN*	11	6946	694	C	A	p.P2	COSM4855094,COSM4855095,COSM931396	Adjacent	Int	Goblet

CGG GTC GTT GCC T-1	1:6 946 602 1	*D 1*		60 21	6 6 0 2 1			87 T		non-cancer	e s ti n a l	
P21A - AAC GTT GTC CTT GAC C-1	CC ND 1:1 1:6 946 602 2	*C C N D 1*	1 1	69 46 60 22	6 9 4 6 6 0 2 2	C	G	p. P2 87 R	COSM2043470,COSM226265,COSM931397	Adjacent non-cancer	I n t e s ti n a l	G M C
P21A - AAG TCT GCA TCC CAC T-1	CC ND 1:1 1:6 946 602 1	*C C N D 1*	1 1	69 46 60 21	6 9 4 6 6 0 2 1	C	G	p. P2 87 A	COSM4855094,COSM4855095,COSM931396	Adjacent non-cancer	I n t e s ti n a l	C hi ef
P21A - AAG TCT GCA TCC CAC T-1	CC ND 1:1 1:6 946 602 2	*C C N D 1*	1 1	69 46 60 22	6 9 4 6 6 0 2 2	C	T	p. P2 87 L	COSM2043470,COSM226265,COSM931397	Adjacent non-cancer	I n t e s ti n a l	C hi ef
P21A - AGG TCC GGT CAG	CC ND 1:1 1:6 946	*C C N D 1*	1 1	69 46 60 21	6 9 4 6 6 0	C	T	p. P2 87 S	COSM4855094,COSM4855095,COSM931396	Adjacent non-	I n t e s ti	G M C

AGGT-1	6021				21					cancer	nal	
P21A-AGGTCCGGTCAGAGGT-1	CCND1:11:69466022	*CCND1*	11	69466022	69466022	C	T	p.P287L	COSM2043470,COSM226265,COSM931397	Adjacent non-cancer	Intestinal	GMC
P21A-CAGTCCTGTGGACGAT-1	CCND1:11:69466022	*CCND1*	11	69466022	69466022	C	T	p.P287L	COSM2043470,COSM226265,COSM931397	Adjacent non-cancer	Intestinal	GMC
P21A-CCTAGCTGTCAAACTC-1	CCND1:11:69466022	*CCND1*	11	69466022	69466022	C	G	p.P287R	COSM2043470,COSM226265,COSM931397	Adjacent non-cancer	Intestinal	GMC
P21A-CGAGCCAAGTGTTAGA-1	EP300:22:41566522	*EP300*	22	41566522	41566522	T	C	p.Y1467H	COSM220521,COSM220522,COSM3357344	Adjacent non-cancer	Intestinal	Enteroendocrine

P21A-CGAGCCAAGTGTTAGA-1	EP300:22:41566522	*EP300*	22	41566522	41566524	TAC	AAA	p.Y1467K		Adjacent non-cancer	Intestinal	Enteroendocrine
P21A-CGTTAGAAGGAGCGTT-1	PTEN:10:89720857	*PTEN*	10	89720857	89720857	C	G	p.Y336*	COSM5290,COSM5300	Adjacent non-cancer	Intestinal	Fibroblast
P21A-CTCATTACAGAGCCAA-1	CCND1:11:69466022	*CCND1*	11	69466022	69466022	C	G	p.P287R	COSM2043470,COSM226265,COSM931397	Adjacent non-cancer	Intestinal	Enteroendocrine
P21A-CTCTAATAGTAACCCT-1	CCND1:11:69466022	*CCND1*	11	69466022	69466022	C	G	p.P287R	COSM2043470,COSM226265,COSM931397	Adjacent non-cancer	Intestinal	PMC
P21A-CTG	CCND1:1	*CCN*	11	6946	694	C	T	p.P2	COSM2043470,COSM226265,COSM931397	Adjacent	Int	Chief

ATA GAG ACG ACG T-1	1:6 946 602 2	*D 1*		60 22	6 6 0 2 2			87 L		non-cancer	estinal	
P21A - CTG ATA GAG ACG ACG T-1	CC ND 1:1 1:6 946 602 1	*C C N D 1*	1 1	69 46 60 21	6 9 4 6 6 0 2 1	C	T	p. P2 87 S	COSM4855094,COSM4855095,COSM931396	Adjacent non-cancer	Intestinal	C hi ef
P21A - GGA CAA GCA TAC TCTT -1	CC ND 1:1 1:6 946 602 2	*C C N D 1*	1 1	69 46 60 22	6 9 4 6 6 0 2 2	C	T	p. P2 87 L	COSM2043470,COSM226265,COSM931397	Adjacent non-cancer	Intestinal	P M C
P21A - TGG GAA GCA CCT ATC C-1	FB XW 7:4: 153 244 184	*F B X W 7*	4	15 32 44 18 4	1 5 3 2 4 4 1 8 4	C	T	p. R6 58 Q	rs759610249,COSM1594354,COSM206684,COSM206685,COSM206686,COSM206687	Adjacent non-cancer	Intestinal	En ter oe nd oc rin e
P21A - TGG GAA GCA CCT	FB XW 7:4: 153 244 092	*F B X W 7*	4	15 32 44 09 2	1 5 3 2 4 4	G	A	p. R6 89 W	COSM1154288,COSM206681,COSM206682,COSM206683,COSM27083,COSM5751359,COSM5751360,COSM5751361,COSM5751362,COSM5751363	Adjacent non-	Intesti	En ter oe nd oc

ATCC-1					092					cancer	nal	rine
P22A-ATCACGATCGCGTTTC-1	RAC1:7:6441974	*RAC1*	7	6441974	6441974	C	T	p.A178V	COSM1154840,COSM389868	Adjacent non-cancer	Intestinal	PMC
P22A-CGCTATCTCCTATTCA-1	KRAS:12:25398220	*KRAS*	12	25398220	25398220	A	T	p.D33E	COSM1511784,COSM1511785,COSM4384682,COSM4384683	Adjacent non-cancer	Intestinal	PC
P22A-CTCGGGAAGATTACCC-1	CCND1:11:69466021	*CCND1*	11	69466021	69466021	C	T	p.P287S	COSM4855094,COSM4855095,COSM931396	Adjacent non-cancer	Intestinal	Chief
P22A-CTCTACGGTTGACGTT-1	CCND1:11:69466022	*CCND1*	11	69466022	69466022	C	G	p.P287R	COSM2043470,COSM226265,COSM931397	Adjacent non-cancer	Intestinal	Enteroendocrine

P22A-GGTGTTATCTAACGGT-1	RAC1:7:6441974	*RAC1*	7	6441974	6441974	C	T	p.A178V	COSM1154840,COSM389868	Adjacent non-cancer	Intestinal	GMC
P22A-TACTCATTCGGATGGA-1	FOXA1:14:38061240	*FOXA1*	14	38061240	38061240	G	T	p.S250Y	COSM433050,COSM5950325	Adjacent non-cancer	Intestinal	GMC
P22A-TTAGTTCTCAACCAAC-1	RAC1:7:6441974	*RAC1*	7	6441974	6441974	C	T	p.A178V	COSM1154840,COSM389868	Adjacent non-cancer	Intestinal	GMC
P22B-ACCCACTGTTCTGTTT-1	CCND1:11:69466018	*CCND1*	11	69466018	69466018	A	G	p.T286A		Cancer	Intestinal	Chief
P22B-CAA	CCND1:1	*CCN*	11	6946	694	C	T	p.P2	COSM4855094,COSM4855095,COSM931396	Cancer	Int	PC

GTTGCAGCGTCCA-1	1:69466021	*D1*		6021	66021			87S			estinal	
P22B-CATATGGTCCGCAGTG-1	CCND1:11:69466019	*CCND1*	11	69466019	69466019	C	T	p.T286I	COSM931395	Cancer	Intestinal	PMC
P22B-CCGTGGAGTTGTCTTT-1	CCND1:11:69466022	*CCND1*	11	69466022	69466022	C	G	p.P287R	COSM2043470,COSM226265,COSM931397	Cancer	Intestinal	PMC
P22B-CGATTGAAGACGCTTT-1	CCND1:11:69466022	*CCND1*	11	69466022	69466022	C	T	p.P287L	COSM2043470,COSM226265,COSM931397	Cancer	Intestinal	PMC
P22B-CGTTAGATCTGT	CCND1:11:6946	*CCND1*	11	69466018	694660	A	G	p.T286A		Cancer	Intesti	PMC

CAAG-1	6018				18						nal	
P22B-CTCCTAGCAGTTCATG-1	CCND1:11:69466022	*CCND1*	11	69466022	69466022	C	G	p.P287R	COSM2043470,COSM226265,COSM931397	Cancer	Intestinal	PMC
P22B-GCACTCTCATACAGCT-1	CCND1:11:69466019	*CCND1*	11	69466019	69466019	C	T	p.T286I	COSM931395	Cancer	Intestinal	PMC
P22B-GGTGAAGGTGCGAAAC-1	CCND1:11:69466022	*CCND1*	11	69466022	69466022	C	T	p.P287L	COSM2043470,COSM226265,COSM931397	Cancer	Intestinal	PMC
P22B-GTCAAGTGTCACCTAA-1	CCND1:11:69466022	*CCND1*	11	69466022	69466022	C	G	p.P287R	COSM2043470,COSM226265,COSM931397	Cancer	Intestinal	GMC

P23A-AAGTCTGGTAAGAGAG-1	CCND1:11:69466022	*CCND1*	11	69466022	69466022	C	G	p.P287R	COSM2043470,COSM226265,COSM931397	Adjacent non-cancer	Diffuse	PMC
P23A-ACAGCTATCTTGAGAC-1	HIST1H3B:6:26031971	*HIST1H3B*	6	26031971	26031971	C	T	p.E106=	rs752372678,COSM4903076	Adjacent non-cancer	Diffuse	MSC
P23A-AGGTCCGCAGACAGGT-1	CCND1:11:69466018	*CCND1*	11	69466018	69466018	A	G	p.T286A		Adjacent non-cancer	Diffuse	GMC
P23A-CACTCCACAAGTTCTG-1	EP300:22:41566475	*EP300*	22	41566475	41566475	A	T	p.H1451L	COSM1034564,COSM1484264,COSM254672	Adjacent non-cancer	Diffuse	GMC
P23B-CAGCATAAGCAC	CCND1:11:69466021	*CCND1*	11	69466021	694660	C	T	p.P287S	COSM4855094,COSM4855095,COSM931396	Cancer	Diffuse	PMC

CGCT-1					21							
P24A-ATCACGAGTTTGCATG-1	NFE2L2:2:178098969	*NFE2L2*	2	178098969	178098970	GC	TT	p.Q26K	COSM1613739	Adjacent non-cancer	Diffuse	PMC
P24A-ATCACGAGTTTGCATG-1	NFE2L2:2:178098969	*NFE2L2*	2	178098969	178098969	G	T	p.Q26K	COSM1009931,COSM132986	Adjacent non-cancer	Diffuse	PMC
P24A-CGGGTCACAAGCGATG-1	RAC1:7:6426907	*RAC1*	7	6426907	6426907	C	T	p.P34S	COSM3640041,COSM3640042	Adjacent non-cancer	Diffuse	Enteroendocrine
P24A-CGGGTCACAAGCGATG-1	RAC1:7:6441974	*RAC1*	7	6441974	6441974	C	T	p.A178V	COSM1154840,COSM389868	Adjacent non-cancer	Diffuse	Enteroendocrine

P24A-GAATGAAAGTAGCGGT-1	RAC1:7:6441974	*RAC1*	7	6441974	6441974	C	T	p.A178V	COSM1154840,COSM389868	Adjacent non-cancer	Diffuse	GMC
P24A-GAATGAATCCTGCTTG-1	RAC1:7:6441974	*RAC1*	7	6441974	6441974	C	T	p.A178V	COSM1154840,COSM389868	Adjacent non-cancer	Diffuse	GMC
P24A-GAGTCCGTCCACGTTC-1	RAC1:7:6441974	*RAC1*	7	6441974	6441974	C	T	p.A178V	COSM1154840,COSM389868	Adjacent non-cancer	Diffuse	EC
P24A-GCGCAACTCGCGGATC-1	CREBBP:16:3786715	*CREBBP*	16	3786715	3786715	A	C	p.L1499R	COSM220497,COSM88752	Adjacent non-cancer	Diffuse	GMC
P24A-TCAATCTTCGGA	RHOA:3:49405953	*RHOA*	3	49405953	494059	C	T	p.G62E	COSM4118486,COSM5883835	Adjacent non-cancer	Diffuse	GMC

TGGA-1					53							
P24B-AGCTTGATCTTCATGT-1	RAC1:7:6441974	*RAC1*	7	6441974	6441974	C	T	p.A178V	COSM1154840,COSM389868	Cancer	Diffuse	Tumor
P24B-ATCCGAATCGTGGTCG-1	CCND1:11:69466022	*CCND1*	11	69466022	69466022	C	G	p.P287R	COSM2043470,COSM226265,COSM931397	Cancer	Diffuse	Tumor
P24B-CACAAACAGACGACGT-1	RAC1:7:6441974	*RAC1*	7	6441974	6441974	C	T	p.A178V	COSM1154840,COSM389868	Cancer	Diffuse	MSC
P24B-CGGAGCTCAATCCAAC-1	CCND1:11:69466022	*CCND1*	11	69466022	69466022	C	T	p.P287L	COSM2043470,COSM226265,COSM931397	Cancer	Diffuse	GMC
P24B-CGTAGG	FBXW7:4:153	*FBX*	4	153244	1532	C	T	p.R658Q	rs759610249,COSM1594354,COSM206684,COSM206685,COSM206686,COSM206687	Cancer	Difu	GMC

CCAGTATAAG-1	244184	*W7*		184	441184						se	
P24B-CTGCCTAAGACAAGCC-1	CTNNB1:3:41266101	*CTNNB1*	3	41266101	41266101	C	G	p.S33C	rs121913400,COSM5669,COSM5673,COSM5677	Cancer	Diffuse	PMC
P24B-CTGCCTAAGACAAGCC-1	CTNNB1:3:41266103	*CTNNB1*	3	41266103	41266103	G	A	p.G34R	rs121913399,COSM3660550,COSM5684,COSM5686	Cancer	Diffuse	PMC
P24B-CTGCCTAAGACAAGCC-1	CTNNB1:3:41266100	*CTNNB1*	3	41266100	41266213	TCTGGAATCCATTCTGGTGCCACTACCACAGCTCCTTCTCTGAGTGGTAAAGGCAATCCTGAGGAAGAGGATGTGGATACCTCCCAAGTCCTGTATGAGTGGGAACAGGGATTT	-	p.G34_S71del		Cancer	Diffuse	PMC
P24B-CTGCCTAAGACAAGCC-1	CTNNB1:3:41266100	*CTNNB1*	3	41266100	41266100	T	G	p.S33A	COSM27311,COSM5682,COSM5683	Cancer	Diffuse	PMC

P24B-CTGCCTAAGACAAGCC-1	CTNNB1:3:41266098	*CTNNB1*	3	41266098	41266112	ACTCTGGAATCCATT	-	p.D32_S37delinsA		Cancer	Diffuse	PMC
P24B-CTGCCTAAGACAAGCC-1	CTNNB1:3:41266098	*CTNNB1*	3	41266098	41266106	ACTCTGGAA	-	p.D32_I35delinsV		Cancer	Diffuse	PMC
P24B-CTGCCTAAGACAAGCC-1	CTNNB1:3:41266098	*CTNNB1*	3	41266098	41266098	A	T	p.D32V	rs121913396,COSM5681,COSM5690,COSM5691	Cancer	Diffuse	PMC
P24B-CTGCCTAAGACAAGCC-1	CTNNB1:3:41266103	*CTNNB1*	3	41266103	41266104	GG	TT	p.G34L	COSM1666841	Cancer	Diffuse	PMC
P24B-GATGCTAGTTTA	RAC1:7:6441974	*RAC1*	7	6441974	6441974	C	T	p.A178V	COSM1154840,COSM389868	Cancer	Diffuse	EC

GCTG-1													
P24B-GCATACAAGTCGAGTG-1	RAC1:7:6441974	*RAC1*	7	6441974	6441974	C	T	p.A178V	COSM1154840,COSM389868	Cancer	Diffuse	EC
P24B-GGTGAAGCATGGTCTA-1	CCND1:11:69466019	*CCND1*	11	69466019	69466019	C	T	p.T286I	COSM931395	Cancer	Diffuse	Enteroendocrine
P24B-GTAGGCCTCTGCAAGT-1	CCND1:11:69466022	*CCND1*	11	69466022	69466022	C	T	p.P287L	COSM2043470,COSM226265,COSM931397	Cancer	Diffuse	Enteroendocrine
P24B-GTAGGCCTCTGCAAGT-1	KRAS:12:25380277	*KRAS*	12	25380277	25380278	GA	TT	p.Q61K	COSM4387500,COSM87298	Cancer	Diffuse	Enteroendocrine
P24B-GTAGGC	KRAS:12:253	*KRAS*	12	25380277	2538	G	T	p.Q61K	rs121913238,COSM1159597,COSM549,COSM550	Cancer	Difu	Enteroend

CTCTGCAAGT-1	80277				0277						se	ocrine
P24B-TCTTTCCAGGTGACCA-1	CCND1:11:69466018	*CCND1*	11	69466018	69466018	A	G	p.T286A		Cancer	Diffuse	Tumor
P24B-TGCTACCTCCACGTGG-1	CDKN2A:9:21970969	*CDKN2A*	9	21970969	21970969	A	T	p.L130Q	COSM13670,COSM18438,COSM28675,COSM33799,COSM3395738,COSM3395739,COSM3788239,COSM3788240,COSM4571148	Cancer	Diffuse	MSC
P24B-TTGCGTCTCCCAAGTA-1	KRAS:12:25378562	*KRAS*	12	25378562	25378562	C	T	p.A146T	rs121913527,COSM1140130,COSM1165198,COSM19404,COSM19905,COSM5967494	Cancer	Diffuse	EC
P25A-ATCATCTAGGGTTTCT-1	CCND1:11:69466022	*CCND1*	11	69466022	69466022	C	T	p.P287L	COSM2043470,COSM226265,COSM931397	Control		Enteroendo

P25A-CGTCACTGTTAGTGGG-1	RAC1:7:6441974	*RAC1*	7	6441974	6441974	C	T	p.A178V	COSM1154840,COSM389868	Control		B
C02A-TGGTTCCGTGGACGAT-1	CCND1:11:69466022	*CCND1*	11	69466022	69466022	C	G	p.P287R	COSM2043470,COSM226265,COSM931397	Control		PMC
P27A-AAGGAGCTCGGCATCG-1	RAC1:7:6441974	*RAC1*	7	6441974	6441974	C	T	p.A178V	COSM1154840,COSM389868	Control		B
P27A-AAGGTTCGTCTCCACT-1	FBXW7:4:153244091	*FBXW7*	4	153244091	153244091	C	T	p.R689Q	COSM1594355,COSM302214,COSM302215,COSM302216,COSM302217	Control		B
P27A-ACACCAAGTCGT	CCND1:11:6946	*CCND1*	11	69466021	694660	C	A	p.P287T	COSM4855094,COSM4855095,COSM931396	Control		B

GGC T-1	602 1				21							
P27A -ACG GCC ACA TGG ATG G-1	RA C1: 7:6 441 974	*RAC1*	7	64 41 97 4	6441974	C	T	p. A1 78 V	COSM1154840,COSM389868	Co ntr ol		En ter oe nd o
P27A -AGC GGT CCA TCG ACG C-1	CC ND 1:1 1:6 946 602 2	*CCND1*	11	69 46 60 22	69466022	C	T	p. P2 87 L	COSM2043470,COSM226265,COS M931397	Co ntr ol		P M C
P27A -AGC GGT CCA TCG ACG C-1	RA C1: 7:6 441 974	*RAC1*	7	64 41 97 4	6441974	C	T	p. A1 78 V	COSM1154840,COSM389868	Co ntr ol		P M C
P27A -ATC CGA AAG AGC TTCT -1	CC ND 1:1 1:6 946 601 8	*CCND1*	11	69 46 60 18	69466018	A	G	p. T2 86 A		Co ntr ol		P M C
P27A -ATG CGA	CC ND 1:1 1:6	*CCN*	11	69 46 60 22	6946	C	T	p. P2 87 L	COSM2043470,COSM226265,COS M931397	Co ntr ol		P M C

TTCAGGTTCA-1	946602 2	*D1*			6022							
P27A-CAAGATCTCGCACTCT-1	PTEN:10:89720853	*PTEN*	10	89720853	89720853	G	C	p.R335P	CD1212290,CM004524	Control		Enteroendo
P27A-CACAGGCCAATGAAAC-1	CCND1:11:69466019	*CCND1*	11	69466019	69466019	C	T	p.T286I	COSM931395	Control		PMC
P27A-CCGTACTGTTACGGAG-1	CCND1:11:69466018	*CCND1*	11	69466018	69466018	A	G	p.T286A		Control		PMC
P27A-CCTAGCTGTTGCCTCT-1	CREBBP:16:3786715	*CREBBP*	16	3786715	3786715	A	T	p.L1499Q	COSM220497,COSM88752	Control		PMC

P27A-CCTCAGTCAGCGTCCA-1	CCND1:11:69466022	*CCND1*	11	69466022	69466022	C	G	p.P287R	COSM2043470,COSM226265,COSM931397	Control		PMC
P27A-CGACTTCCAAGTCTAC-1	CCND1:11:69466022	*CCND1*	11	69466022	69466022	C	G	p.P287R	COSM2043470,COSM226265,COSM931397	Control		Fibroblast
P27A-CGAGAAGAGTGGTAGC-1	RAC1:7:6441974	*RAC1*	7	6441974	6441974	C	T	p.A178V	COSM1154840,COSM389868	Control		PMC
P27A-CGCCAAGCACAGCCCA-1	RAC1:7:6441974	*RAC1*	7	6441974	6441974	C	T	p.A178V	COSM1154840,COSM389868	Control		B
P27A-CTAGAGTCAGGA	PDGFRA:4:55144147	*PDGFRA*	4	55144147	551441	A	G	p.N659S		Control		PMC

ATGC-1					47							
P27A-CTGCTGTTCGGTTCGG-1	SMARCA4:19:1106926	*SMARCA4*	19	11106926	11106928	AGA	-	p.K546del	COSM5576272,COSM5576273	Control		Fibroblast
P27A-GACACGCTCACAACGT-1	RAC1:7:6441974	*RAC1*	7	6441974	6441974	C	T	p.A178V	COSM1154840,COSM389868	Control		B
P27A-GACGCGTCACCACGTG-1	CCND1:11:69466021	*CCND1*	11	69466021	69466021	C	G	p.P287A	COSM4855094,COSM4855095,COSM931396	Control		PMC
P27A-GACGCGTCACCACGTG-1	CCND1:11:69466022	*CCND1*	11	69466022	69466022	C	T	p.P287L	COSM2043470,COSM226265,COSM931397	Control		PMC
P27A-GACGCG	NFE2L2:2:178	*NFE2*	2	178098	1780	C	T	p.G81D	COSM132957,COSM132961	Control		PMC

TTCGAGAACG-1	098803	*L2*		803	98803							
P27A-GCAATCACAATGGATA-1	RAC1:7:6441974	*RAC1*	7	6441974	6441974	C	T	p.A178V	COSM1154840,COSM389868	Control		Fibroblast
P27A-GCATACATCGTCACGG-1	RAC1:7:6441974	*RAC1*	7	6441974	6441974	C	T	p.A178V	COSM1154840,COSM389868	Control		EC
P27A-GCGCCAACACGAGAGT-1	KRAS:12:25380275	*KRAS*	12	25380275	25380276	TT	CA	p.Q61L	COSM1168052	Control		B
P27A-GCGCCAACACGAGAGT-1	KRAS:12:25380281	*KRAS*	12	25380281	25380281	T	C	p.A59=	COSM1162236,COSM1162237,COSM5507485,COSM5507486	Control		B

P27A-GGCAATTCACTTCTGC-1	CCND1:11:69466022	*CCND1*	11	69466022	69466022	C	T	p.P287L	COSM2043470,COSM226265,COSM931397	Control		PMC
P27A-GGCAATTCAGTAAGCG-1	SMARCA4:19:11106926	*SMARCA4*	19	11106926	11106928	AGA	-	p.K546del	COSM5576272,COSM5576273	Control		PMC
P27A-GTCACGGAGATCGGGT-1	FBXW7:4:153244185	*FBXW7*	4	153244185	153244185	G	T	p.R658=	COSM1427626,COSM167197,COSM167198,COSM167199,COSM22967,COSM4837611,COSM4837612,COSM4837613,COSM4837614,COSM4837615	Control		B
P27A-GTCTCGTAGCTAGGCA-1	CCND1:11:69466022	*CCND1*	11	69466022	69466022	C	G	p.P287R	COSM2043470,COSM226265,COSM931397	Control		Fibroblast
P27A-TCAATCTCAAGG	CDKN2A:9:2197	*CDKN2A*	9	21970971	21970971	G	T	p.Y129*	COSM126614,COSM13221,COSM28562,COSM3788241	Control		B

ACAC-1	0971				71							
P27A-TCCACACAGACCTAGG-1	KRAS:12:25378647	*KRAS*	12	25378647	25378647	T	A	p.K117N	COSM1256061,COSM1562192,COSM19940,COSM28519	Control		PMC
P27A-TTAGGCAGTACCCAAT-1	RAC1:7:6441974	*RAC1*	7	6441974	6441974	C	T	p.A178V	COSM1154840,COSM389868	Control		PMC
P27A-TTTATGCGTACCGAGA-1	CCND1:11:69466018	*CCND1*	11	69466018	69466018	A	G	p.T286A		Control		PMC
P29A-AAAGCAAAGATGGCGT-1	RAC1:7:6441974	*RAC1*	7	6441974	6441974	C	T	p.A178V	COSM1154840,COSM389868	Control		B
P29A-AGGCCG	NFE2L2:2:178	*NFE2*	2	178098	1780	G	T	p.Q26K	COSM1009931,COSM132986	Control		PMC

TTCTGATTCT-1	098969	*L2*		969	989969							
P29A-ATCGAGTTCAGGCCCA-1	PTEN:10:89725055	*PTEN*	10	89725055	89725055	C	G	p.Y346*	COSM5310,COSM685100	Control		B
P29A-ATTGGACGTCCATCCT-1	RAC1:7:6441974	*RAC1*	7	6441974	6441974	C	T	p.A178V	COSM1154840,COSM389868	Control		Chief
P29A-CAACTAGTCTCGGACG-1	RAC1:7:6441974	*RAC1*	7	6441974	6441974	C	T	p.A178V	COSM1154840,COSM389868	Control		PMC
P29A-CACACCTAGGTGATAT-1	RAC1:7:6441974	*RAC1*	7	6441974	6441974	C	T	p.A178V	COSM1154840,COSM389868	Control		B

P29A-CACACCTCAGATTGCT-1	EIF1AX:X:20156734	*EIF1AX*	X	20156734	20156734	C	T	p.G8E	COSM3036419,COSM4829462,COSM5625587	Control		Enteroendo
P29A-CCATTCGTCTACCAGA-1	RAC1:7:6441974	*RAC1*	7	6441974	6441974	C	T	p.A178V	COSM1154840,COSM389868	Control		PMC
P29A-CTACATTGTCTCGTTC-1	FBXW7:4:153244185	*FBXW7*	4	153244185	153244185	G	T	p.R658=	COSM1427626,COSM167197,COSM167198,COSM167199,COSM22967,COSM4837611,COSM4837612,COSM4837613,COSM4837614,COSM4837615	Control		B
P29A-CTCTAATCATAGAAAC-1	EIF1AX:X:20156720	*EIF1AX*	X	20156720	20156720	G	C	p.R13G	COSM5899335	Control		B
P29A-GACCTGGAGGTG	EIF1AX:X:20156731	*EIF1AX*	X	20156731	201567	C	T	p.G9D	COSM3372213	Control		EC

CAA C-1					31							
P29A-GCATGTAGTATCAGTC-1	RAC1:7:6441974	*RAC1*	7	6441974	6441974	C	T	p.A178V	COSM1154840,COSM389868	Control		B
P29A-GGAGCAACAGGGCATA-1	RAC1:7:6441974	*RAC1*	7	6441974	6441974	C	T	p.A178V	COSM1154840,COSM389868	Control		Enteroendo
P29A-TACGGTAGTCTTCTCG-1	JUN:1:59248409	*JUN*	1	59248409	59248409	C	T	p.E112K	COSM681630,COSM681631	Control		B
P29A-TCAATCTAGAACAATC-1	RAC1:7:6441974	*RAC1*	7	6441974	6441974	C	T	p.A178V	COSM1154840,COSM389868	Control		PMC

Supplementary Table 7. Pathways and cell counts related to mutated genes

**Cell count**	Adjacent non-cancer	Cancer	
**Cell cycle**	69	201	(CCND1,CDK4,CDKN2A,RB1)
**Chromatin histone modifiers**	10	7	(CREBBP, EP300)
**Chromatin SWI/SNF complex**	0	9	(SMARC4, SMARCB1)
**Genome integrity**	1	1	
**Histone modification**	0	2	
**MAPK signaling**	6	25	(KRAS)
**Other signaling**	91	96	(GNAS,PTPN11,RAC1,RHOA)
**PI3K signaling**	5	6	
**Protein homeostasis/ubiquitination**	10	8	
**RTK signaling**	2	0	
**TGFB signaling**	0	3	
**Transcription factor**	4	1	
**Wnt/B-catenin signaling**	0	8	

Supplementary Table 8. DEGs with CAF subtypes

State	Markers	State	Markers	State	Markers	State	Markers	State	Markers	State	Markers
iCAF	*CXCL1*	iCAF	*COL6A3*	iCAF	*SULF1*	myCAF	*TPM1*	myCAF	*CES1*	inCAF	*PDGFRA*
iCAF	*IL8*	iCAF	*SPON2*	iCAF	*PDPN*	myCAF	*PLN*	myCAF	*SCPEP1*	inCAF	*CAV1*
iCAF	*MMP1*	iCAF	*TNFAIP6*	iCAF	*IER3*	myCAF	*ENPP2*	myCAF	*PROM1*	inCAF	*TBXAS1*
iCAF	*MMP3*	iCAF	*COL1A2*	iCAF	*STC1*	myCAF	*NPNT*	myCAF	*FHL1*	inCAF	*IL1RL1*
iCAF	*HBB*	iCAF	*SPARC*	iCAF	*FTH1*	myCAF	*TPM2*	myCAF	*COL4A1*	inCAF	*CXCL14*
iCAF	*CHI3L1*	iCAF	*CXCL13*	iCAF	*COL3A1*	myCAF	*DES*	myCAF	*LMOD1*	inCAF	*ID3*
iCAF	*CXCL6*	iCAF	*CXCL10*	iCAF	*ISLR*	myCAF	*WFDC2*	myCAF	*PDLIM7*	inCAF	*DIO2*
iCAF	*CST1*	iCAF	*WNT5A*	iCAF	*FDCSP*	myCAF	*LTBP1*	inCAF	*GHRL*	inCAF	*CARD16*
iCAF	*CTHRC1*	iCAF	*CTSK*	iCAF	*CSF3*	myCAF	*CKB*	inCAF	*TM4SF1*	inCAF	*FAM105A*
iCAF	*CXCL5*	iCAF	*STEAP1*	iCAF	*CXCL2*	myCAF	*LPP*	inCAF	*IGFBP3*	inCAF	*NSG1*
iCAF	*MT2A*	iCAF	*INHBA*	iCAF	*COL10A1*	myCAF	*CNN1*	inCAF	*POSTN*	inCAF	*CAV2*
iCAF	*FN1*	iCAF	*COL12A1*	iCAF	*TNFRSF12A*	myCAF	*WFDC1*	inCAF	*F3*	inCAF	*DMKN*
iCAF	*IGFBP5*	iCAF	*TIMP1*	myCAF	*MYH11*	myCAF	*KCNMB1*	inCAF	*TPSAB1*	inCAF	*RGS10*
iCAF	*BGN*	iCAF	*PLAU*	myCAF	*TAGLN*	myCAF	*DSTN*	inCAF	*PLAT*	inCAF	*FENDRR*
iCAF	*COL1A1*	iCAF	*IGFBP7*	myCAF	*HHIP*	myCAF	*SYNPO2*	inCAF	*MFGE8*	inCAF	*EDIL3*
iCAF	*MMP13*	iCAF	*HBA2*	myCAF	*NPY*	myCAF	*MYL6*	inCAF	*PTGS1*	inCAF	*C8orf4*
iCAF	*ASPN*	iCAF	*RARRES2*	myCAF	*SOSTDC1*	myCAF	*NDUFA4*	inCAF	*AGT*	inCAF	*GMFG*
iCAF	*THY1*	iCAF	*IL7R*	myCAF	*PTN*	myCAF	*FLNA*	inCAF	*HSD17B2*	inCAF	*ENHO*
iCAF	*CXCL3*	iCAF	*MMP10*	myCAF	*MYL9*	myCAF	*MAP1B*	inCAF	*ID1*		
iCAF	*THBS2*	iCAF	*SAA1*	myCAF	*MYLK*	myCAF	*CD9*	inCAF	*APOC1*		

Supplementary Table 9. percentage of cell types in Adjacent non-cancer and GC

Cell type	Adjacent non-cancer tissue (%)	Gastric Cancer (%)
Cancer	2.1	14.4
Chief	4.3	2.8
EC	7.3	9.3
Enteroendo	7.5	5.5
Fibroblast	9.2	8.6
GMC	40	19.1
Goblet	0.8	0.7
MSC	3	20.3
PC1	4.8	3.6
PC2	0.5	5
PMC	20.5	10.9
