# Supplementary material for: Single-cell analysis of gastric pre-cancerous and cancer lesions reveals cell lineage diversity and intratumoral heterogeneity
Source: NPJ Precis Oncol. 2022 Jan 27;6:9. doi: 10.1038/s41698-022-00251-1 (PMC8795238; doi:10.1038/s41698-022-00251-1)
Supplement: Supplementary file 2 — REPORTING SUMMARY [file 41698_2022_251_MOESM2_ESM.pdf]

nature portfolio

Corresponding author(s): NPJPRECISIONONCOLOGY-01454R1

Last updated by author(s): Nov 25, 2021

Reporting Summary

Nature Portfolio wishes to improve the reproducibility of the work that we publish. This form provides structure for consistency and transparency in reporting. For further information on Nature Portfolio policies, see our Editorial Policies and the Editorial Policy Checklist.

Statistics

For all statistical analyses, confirm that the following items are present in the figure legend, table legend, main text, or Methods section.

n/a	Confirmed	
☐	☒	The exact sample size (n) for each experimental group/condition, given as a discrete number and unit of measurement
☐	☒	A statement on whether measurements were taken from distinct samples or whether the same sample was measured repeatedly
☐	☒	The statistical test(s) used AND whether they are one- or two-sided<br>*Only common tests should be described solely by name; describe more complex techniques in the Methods section.*
☒	☐	A description of all covariates tested
☐	☒	A description of any assumptions or corrections, such as tests of normality and adjustment for multiple comparisons
☐	☒	A full description of the statistical parameters including central tendency (e.g. means) or other basic estimates (e.g. regression coefficient) AND variation (e.g. standard deviation) or associated estimates of uncertainty (e.g. confidence intervals)
☐	☒	For null hypothesis testing, the test statistic (e.g. F, t, r) with confidence intervals, effect sizes, degrees of freedom and P value noted<br>*Give P values as exact values whenever suitable.*
☒	☐	For Bayesian analysis, information on the choice of priors and Markov chain Monte Carlo settings
☒	☐	For hierarchical and complex designs, identification of the appropriate level for tests and full reporting of outcomes
☐	☒	Estimates of effect sizes (e.g. Cohen's d, Pearson's r), indicating how they were calculated

Our web collection on statistics for biologists contains articles on many of the points above.

Software and code

Policy information about availability of computer code

Data collection	No software was used.
Data analysis	Cell Ranger (2.1.1), R (3.4.1,3.5.0,3.5.1,3.6.1), Seurat (2.4), SingleR (1.7.1), MuSic (0.2.0), VarTrix (1.0), inferCNV (1.10.1), Monocle (2.22.0), e1071 (1.7), Cytoscape (3.7.0), ReactomeFI (2.7.0), sva (3.38.0), survival (3.2)

For manuscripts utilizing custom algorithms or software that are central to the research but not yet described in published literature, software must be made available to editors and reviewers. We strongly encourage code deposition in a community repository (e.g. GitHub). See the Nature Portfolio guidelines for submitting code & software for further information.

Data

Policy information about availability of data

All manuscripts must include a data availability statement. This statement should provide the following information, where applicable:

- Accession codes, unique identifiers, or web links for publicly available datasets
- A description of any restrictions on data availability
- For clinical datasets or third party data, please ensure that the statement adheres to our policy

GSE150290

Field-specific reporting

Please select the one below that is the best fit for your research. If you are not sure, read the appropriate sections before making your selection.

☒ Life sciences ☐ Behavioural & social sciences ☐ Ecological, evolutionary & environmental sciences

For a reference copy of the document with all sections, see nature.com/documents/nr-reporting-summary-flat.pdf

Life sciences study design

All studies must disclose on these points even when the disclosure is negative.

Sample size	The sample size was 24 patients. This was not pre-calculated. we enrolled as participants as possible in the study timeframe.
Data exclusions	No data exclusions
Replication	No data replication
Randomization	In this study, there were no experimental groups since the objective was to identify landscape of gastric cancer microenvironment.
Blinding	No blinding was necessary since participants were not allocated into experimental groups. All participants performed the same data collection protocol.

Reporting for specific materials, systems and methods

We require information from authors about some types of materials, experimental systems and methods used in many studies. Here, indicate whether each material, system or method listed is relevant to your study. If you are not sure if a list item applies to your research, read the appropriate section before selecting a response.

Materials & experimental systems

n/a	Involved in the study	
☐	☒	Antibodies
☒	☐	Eukaryotic cell lines
☒	☐	Palaeontology and archaeology
☒	☐	Animals and other organisms
☐	☒	Human research participants
☒	☐	Clinical data
☒	☐	Dual use research of concern

Methods

n/a	Involved in the study	
☒	☐	ChIP-seq
☒	☐	Flow cytometry
☒	☐	MRI-based neuroimaging

Antibodies

Antibodies used	anti-SRF; anti-IGFBP5; anti-MRTFA
Validation	Cell signaling Tech, Cat# D71A9; R&D systems, Cat# AF875; Abcam, Cat# ab49311

Human research participants

Policy information about studies involving human research participants

Population characteristics	For the 24 patients, average age was 59.4 years, Epstein-Barr virus infection was 4.2%, and Helicobacter pylori infection was 79.2%. There were 8 females and 16 males.
Recruitment	Participants were recruited from a sample of gastric cancer patients from the Severance Hospital setting. Participants had no history of gastric cancer.
Ethics oversight	Severance Hospital (IRB 4-2017-1131)

Note that full information on the approval of the study protocol must also be provided in the manuscript.
